# Eradication of Therapy-Resistant Cancer Stem Cells by Novel Telmisartan Derivatives

**DOI:** 10.1021/acs.jmedchem.4c01865

**Published:** 2024-12-18

**Authors:** Anna M. Schoepf, Maximilian Gebhart, Martin Federspiel, Isabel Heidegger, Martin Puhr, Madlen Hotze, Marcel Kwiatkowski, Andreas Pircher, Dominik Wolf, Sieghart Sopper, Ronald Gust, Stefan Salcher

**Affiliations:** † CCB - Centrum for Chemistry and Biomedicine, Department of Pharmaceutical Chemistry, Institute of Pharmacy, CMBI - Center for Molecular Biosciences Innsbruck, University of Innsbruck, Innsbruck 6020, Austria; ‡ Department of Urology, Medical University of Innsbruck, Innsbruck 6020, Austria; § Department of Biochemistry and Center for Molecular Biosciences Innsbruck, University of Innsbruck, Innsbruck 6020, Austria; ∥ Department of Internal Medicine V, Hematology and Oncology, Tyrolean Cancer Research Institute (TKFI), Comprehensive Cancer Center Innsbruck (CCCI), Medical University of Innsbruck, Innsbruck 6020, Austria

## Abstract

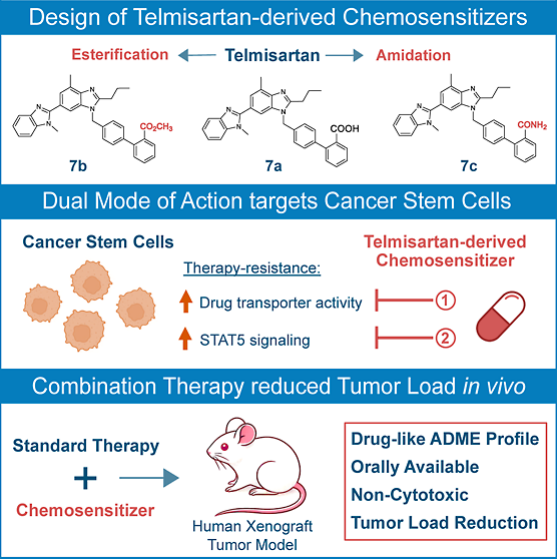

The present structure–activity relationship study investigates the development of novel chemosensitizers targeting therapy-resistant cancer stem cells (CSCs). We used 4′-((2-propyl-1*H*-benzo[*d*]imidazole-1-yl)methyl)-[1,1′-biphenyl]-2-carboxylic acid, derived from the angiotensin II type 1 receptor blocker telmisartan, as a lead structure, demonstrating that the biphenyl moiety is essential for chemosensitizing activity. Introducing a methyl carboxylate or carboxamide instead of the COOH-group significantly enhanced this effect, leading to the development of highly potent compounds. These novel, noncytotoxic chemosensitizers effectively target CSCs and overcome drug resistance by interfering with CSC persistence mechanisms—hyperactivated STAT5 signaling and increased drug transporter activity—with demonstrated efficacy in leukemia, ovarian, and prostate cancers. The carboxamide of telmisartan (telmi-amide, **7c**) significantly reduced tumor growth in an imatinib-resistant leukemia xenograft model, both as monotherapy and combined with imatinib, showing promising oral bioavailability and tolerability. In summary, telmisartan derivatives act as effective chemosensitizers and offer an innovative strategy for targeting CSCs in various malignant diseases.

## 1 Introduction

Chronic myeloid leukemia (CML) therapy represents a paradigm of target-oriented cancer treatment, as tyrosine kinase inhibitors (TKIs) specifically inactivate the disease-driving BCR-ABL1 kinase. The introduction of TKIs led to a remarkable improvement in therapy and transformed CML from a fatal to a chronic disease with survival rates of over 90%.^1^ However, a complete cure is hindered by the emerging pool of quiescent and TKI-insensitive cancer stem cells (CSCs), also known as leukemia-initiating cells (LICs), which are already detectable at the stage of diagnosis.^2^ CSCs have tumor-promoting and self-renewing capabilities and are resistant to conventional TKI therapies, leading to disease perpetuation and recurrence.^3^ Notably, CSC persistence is not dependent on BCR-ABL1 activity,^4^ indicating the need for a kinase-independent approach to target CSCs with innovative therapeutics^5^ to ultimately cure CML.

Inherent and acquired resistance to clinically applied anticancer agents constitutes a major hurdle in cancer therapy nowadays.^6^ Thus, elucidating the mechanisms that promote the persistence of therapy-resistant CSCs will provide valuable information for the management of various cancers and may significantly improve clinical outcomes, particularly for malignancies treated with kinase inhibitors.^7^ Multidrug resistance (MDR) is a phenomenon of cross-resistance that arises from exposure to a multiplicity of structurally unrelated chemotherapeutic drugs. Among others, transmembrane ATP-binding cassette (ABC) transporter proteins are frequently involved in this process.^8^ Consistently, the resistance of leukemic CSCs to conventional therapies can be partially explained by the high expression of these drug transporters.^7^ Physiologically, the ABC transporter P-glycoprotein (also known as P-gp, ABCB1, or MDR1) and MDR-related proteins protect cells against diverse toxins by regulating the expulsion of xenobiotics across cell membranes in an energy-dependent manner. Drug transporter expression is prevalently elevated in CML, acute myeloid leukemia (AML), and different solid cancers.^9,10^ Since frequently applied anticancer agents are substrates of transporter proteins, such as ABCB1, their efflux may drastically decrease intracellular drug concentrations and result in a poor response to therapy.^11^

Recent strategies have focused on the prevention of drug transporter formation or on the design of specific inhibitors of drug transporter activity to overcome MDR.^8^ These inhibitors are defined as chemosensitizers and are thought to restore the efficacy of antineoplastic drugs in resistant cancer cells.^12,13^ However, although three generations of drug transporter inhibitors have been developed and some compounds have even entered clinical trials, no significant clinical advantage has been observed in therapy-resistant patients to date. Neither verapamil, valspodar, nor elacridar (first, second, and third generation ABC transporter inhibitors, respectively) achieved a substantial survival benefit. Notably, occurring side effects impeded their successful applicability and widespread clinical use.^12^ Therefore, discovering new and efficient chemosensitizers that are applicable in noncytotoxic doses is of high importance. Such agents could enhance the effectiveness of anticancer drugs in combination therapy while minimizing toxic side effects, even after failure of first-line therapy.

In previous structure–activity relationship (SAR) studies, we determined that derivatives of the angiotensin II type 1 receptor blocker (ARB) telmisartan sensitize multidrug-resistant CML cells to TKI therapy.^14−17^ Our findings indicate that coadministration of these compounds enhances the sensitivity of TKI-resistant leukemia K562 cells to imatinib therapy and promotes cell death in a dose-dependent manner. So far, the detailed molecular mechanisms are not completely understood, however, we could demonstrate that these newly developed chemosensitizers repress STAT5 (signal transducer and activator of transcription 5) signaling when combined with the TKI.^14−17^ The transcription factor STAT5 is a crucial signaling node in the maintenance and relapse of CML.^18^ Its excessive activation is a negative prognostic factor in myeloid malignancies,^19^ since STAT5 represents a guardian protein of quiescence and stemness and is therefore closely linked to the emergence of resistance to TKIs.^20^ STAT5 is a direct transcriptional regulator of the ABCB1 drug transporter. Both, STAT5 and ABCB1 are activated in primary leukemic cells derived from patients in blast crisis, underlining the clinical relevance of these stemness-related proteins.^21^

In the present work, we aimed to optimize telmisartan and the 4′-((2-propyl-1*H*-benzo[*d*]imidazole-1-yl)methyl)-[1,1′-biphenyl]-2-carboxylic acid by transformation of the 2-COOH group to a methyl ester (2-CO_2_CH_3_) or carboxamide (2-CONH_2_). In the latter case, also the influence of the substitution pattern (ortho, meta, or para position of the substituents) on the biological effects was of interest. Furthermore, the biphenyl moiety was exchanged for a phenyl ring (→ 2-((2-propyl-1*H*-benzo[*d*]imidazole-1-yl)methyl)benzoic acid), which was then modified in the same way.

This study provides a deeper insight into the structural requirements for the optimization of telmisartan and its derivatives as nontoxic chemosensitizers. In addition, the results suggest the applicability of these derivatives to target and eradicate the CSC pool in CML as well as the therapy-resistant CSCs in solid tumors such as ovarian, prostate, lung, and breast cancer. To uncover the functional mechanism of the compounds, their effects on critical stem cell markers such as the ABC transporter (increased drug transporter activity) and STAT5 (excessive signaling) were analyzed.

## 2 Results

### 2.1 Synthesis and General Cytotoxicity of the Developed Chemosensitizers

**Figure 1:**
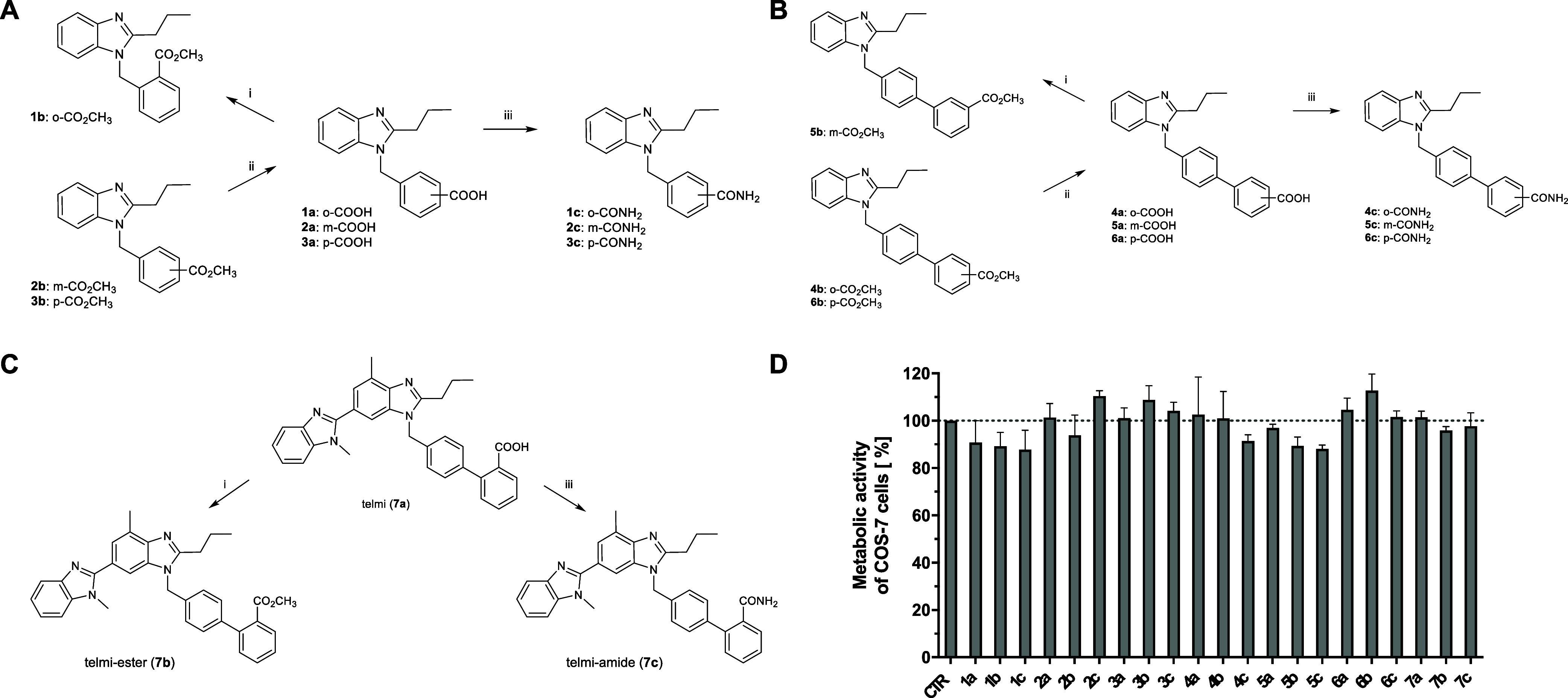
Synthesis and general cytotoxicity of the developed chemosensitizers. Final steps within the synthesis route of (A) the phenyl derivatives **1a**–**c**, **2a**–**c**, and **3a**–**c** or (B) the biphenyl derivatives **4a**–**c**, **5a**–**c**, and **6a**–**c**. (C) Synthesis of the methyl ester (**7b**) and the carboxamide (**7c**) of telmisartan. Reagents and conditions: (i) SOCl_2_, anhyd. MeOH, 0 °C to rt; (ii) KOH, EG, H_2_O, 160 °C; (iii) PyBOP, NH_4_Cl, DIPEA, DMF, 0 °C to rt. (D) Metabolic activity of COS-7 cells treated with vehicle (CTR, DMSO) or 10 μM of **1a**–**c**, **2a**–**c**, **3a**–**c**, **4a**–**c**, **5a**–**c**, **6a**–**c**, and **7a**–**c** for 72 h, respectively. Data determined by a colorimetric MTT assay represent the mean + SEM of ≥3 independent experiments with three replicates each.

The target compounds were prepared in accordance with previously described procedures.^17,22^ The final steps within the synthesis route of the phenyl series (**1a**–**c** to **3a**–**c**; Figure 1A), the biphenyl series (**4a**–**c** to **6a**–**c**; Figure 1B), and the telmisartan analogues (telmi-ester (**7b**), telmi-amide (**7c**); Figure 1C) are depicted in Figure 1. The entire synthesis path is shown in Scheme S1, submitted as Supporting Information.

In a first step, 1,2-phenylenediamine was reacted with butyric anhydride in concentrated (conc.) hydrochloric acid (HCl) to the *N*,*N’*-(1,2-phenylene)dibutyramide (**I**), which was then heated in 4 N HCl to obtain the synthon 2-propyl-1*H*-benzo[*d*]imidazole (**II**) (Scheme S1).

Treatment of **II** with 2-(bromomethyl)benzonitrile, methyl 3-(bromomethyl)benzoate, or methyl 4-(bromomethyl)benzoate in the presence of sodium hydride (NaH) as a base yielded the 1-benzyl-2-propyl-1*H*-benzo[*d*]imidazoles **III** (*o*-CN), **2b** (*m*-CO_2_CH_3_), and **3b** (*p*-CO_2_CH_3_). Hydrolysis of these compounds with potassium hydroxide (KOH) in ethylene glycol (EG) and water gave the benzoic acids **1a** (*o*-COOH), **2a** (*m*-COOH), and **3a** (*p*-COOH, Figure 1A). Stirring of **1a** in anhydrous (anhyd.) methanol (MeOH) with thionyl chloride (SOCl_2_) resulted in the methyl ester **1b** (*o*-CO_2_CH_3_^23^). The carboxamides (**1c** (*o*-CONH_2_), **2c** (*m*-CONH_2_), and **3c** (*p*-CONH_2_)) were formed in anhyd. dimethylformamide (DMF) from **1a**, **2a**, **3a**, and ammonium chloride (NH_4_Cl), respectively, by a coupling reaction with the phosphonium based reagent benzotriazol-1-yloxytris(pyrrolidino)phosphonium hexafluorophosphate (PyBOP) and the Hünig’s base *N,N*-diisopropylethylamine (DIPEA).

1-([1,1′-Biphenyl]-4-ylmethyl)-2-propyl-1*H*-benzo[*d*]imidazoles were synthesized by *N*-alkylation of **II** with correspondingly substituted 4-(bromomethyl)-1,1′-biphenyl derivatives. Since the starting materials methyl 4′-(bromomethyl)-[1,1′-biphenyl]-4-carboxylate (**V**) and 4′-(bromomethyl)-[1,1′-biphenyl]-3-carbonitrile (**VII**) were commercially not available, their synthesis was performed in-house (Scheme S1). A Suzuki coupling of methyl 4-bromobenzoate or 3-bromobenzonitrile with *p*-tolylboronic acid using the catalyst tetrakis(triphenylphosphine)palladium(0) (Pd(PPh_3_)_4_) and sodium carbonate (Na_2_CO_3_, 2 N) in dioxane, yielded the intermediates **IV** and **VI**, respectively.^24^ Bromination of their methyl groups was carried out with *N*-bromosuccinimide (NBS) and the radical initiator benzoyl peroxide in anhyd. carbon tetrachloride (CCl_4_).^25^

*N*-Alkylation of **II** with the biphenyl moieties **VII** (*m*-CN), methyl (4′-bromomethyl)-[1,1′-biphenyl]-2-carboxylate (*o*-CO_2_CH_3_), or **V** (*p*-CO_2_CH_3_) as described above, gave the meta-nitrile intermediate **VIII**, the ortho- (**4b**) or the para-ester (**6b**). Alkaline hydrolysis of **VIII**, **4b**, or **6b** to **4a** (*o*-COOH), **5a** (*m*-COOH), and **6a** (*p*-COOH), esterification of **5a** to **5b** (*m*-CO_2_CH_3_), and synthesis of the carboxamides **4c** (*o*-CONH_2_), **5c** (*m*-CONH_2_), and **6c** (*p*-CONH_2_) from **4a**–**c** were performed as described above (Figure 1B).

Finally, telmisartan (telmi, **7a**) was transformed to the methyl ester (telmi-ester, **7b**) and the carboxamide (telmi-amide, **7c**) applying the same reaction pathways (Figure 1C).

The target compounds **1a**–**c** to **6a**–**c**, and **7b**–**c** were characterized by ^1^H and ^13^C NMR spectroscopy (Supporting Information Figures S1–S20). High-performance liquid chromatography (HPLC, Supporting Information Figures S21–S40) was performed to ensure a purity of >95%. Moreover, high-resolution mass spectroscopy (HRMS) was carried out to confirm the correct mass of the final products (Supporting Information Figures S41–S60). The synthesis protocols of intermediates as well as the analytical data of target compounds (all targets are depicted in Scheme 1) are provided as Supporting Information.

**Scheme 1:**
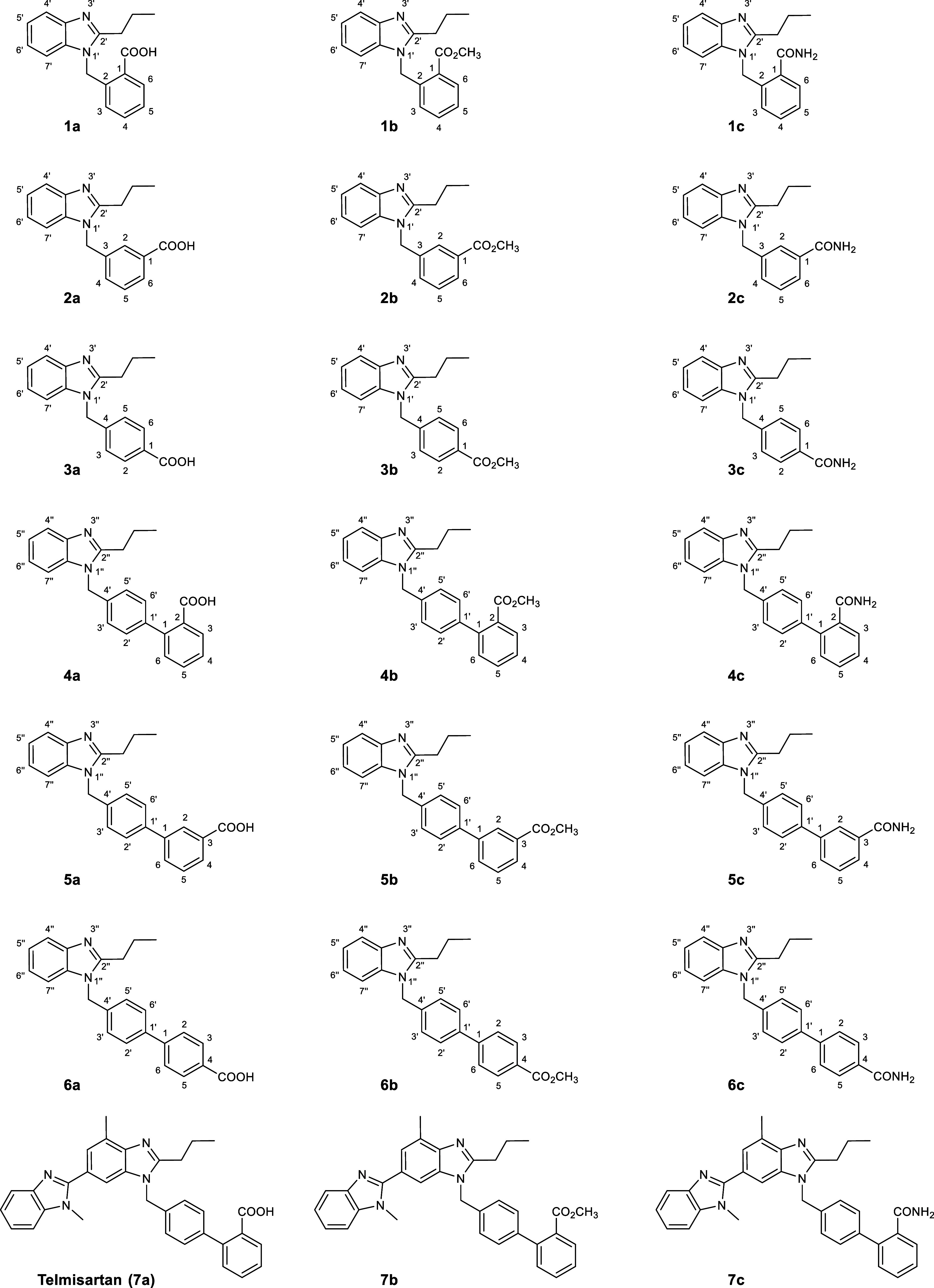
Synthesized Target Compounds that Have Been Biologically Evaluated in This Work The phenyl derivatives are designated **1**–**3** and their biphenyl analogs **4**–**6**. The derivatives of telmisartan have the number **7**. The letters correspond to the carboxylic acids (**a**), the methyl esters (**b**), and the carboxamides (**c**), respectively. The numbering of the atoms refers to the assignment of the protons in the ^1^H NMR spectra.

To assess the potential cytotoxicity, the nonmalignant green monkey kidney fibroblast-like cell line COS-7 was incubated with 10 μM of each derivative for 72 h. The change in cell viability was determined by a modified MTT (3-(4,5-dimethyl-2-thiazolyl)-2,5-diphenyl-2*H*-tetrazolium bromide) colorimetric assay. As none of the compounds markedly repressed the metabolic activity of COS-7 cells (Figure 1D), the novel telmisartan derivatives can be stated as noncytotoxic in nonmalignant cells.

### 2.2 Selective Targeting of TKI-Resistant CML Cells Enhances Therapy Response

In order to investigate whether the compounds are able to resensitize CML cells for TKI treatment, therapy-resistant human K562-R CML cells were incubated for 72 h with imatinib at the inactive concentration of 1 μM together with each chemosensitizer (10 μM). The potential reduction in cell viability was determined using the modified MTT assay.

**Figure 2:**
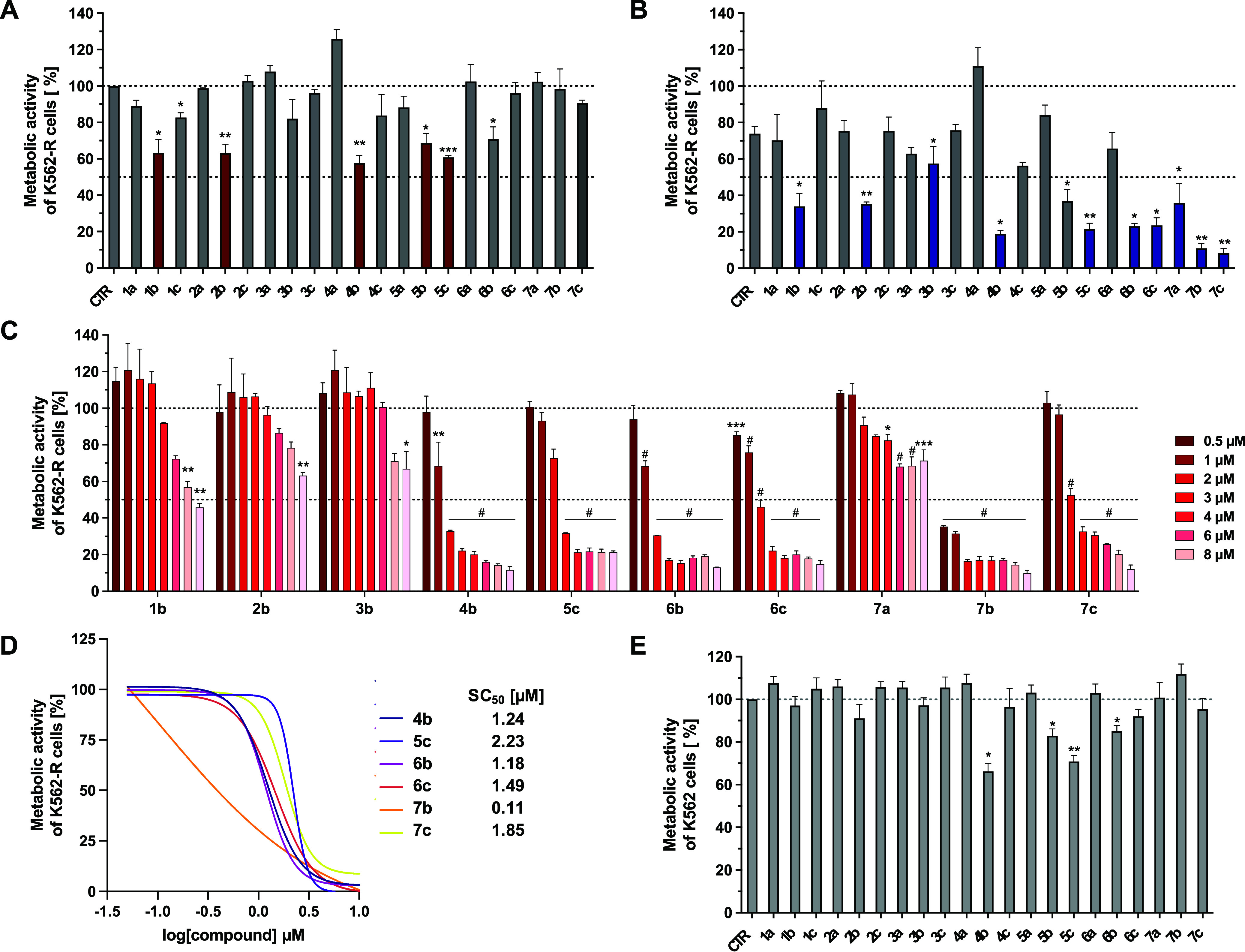
Selective targeting of TKI-resistant CML cells enhances therapy response. K562-R cells were treated with (A) **1a–c**, **2a**–**c**, **3a**–**c**, **4a**–**c**, **5a**–**c**, **6a**–**c**, and **7a–c,** alone (10 μM each, **1A**), or (B) in combination with imatinib (1 μM, **1B**). CTR represents the vehicle-treated control (DMSO) either without (Figure 1A) or with (Figure 1B) imatinib. After 72 h, the metabolic activity of the cells was determined by a modified MTT assay. Significant reductions of metabolic activity relative to the vehicle-treated control (CTR) are indicated. Statistical analysis was done with the Student’s paired *t*-test; *p < 0.05, **p < 0.01, ***p < 0.001. Data represent the mean + SEM of ≥3 independent experiments with three replicates each. (C) Concentration-dependent impact of **1b**, **2b**, **3b**, **4b**, **5c**, **6b**, **6c**, **7a**–**7c** on the metabolic activity of K562-R cells, cotreated with imatinib (1 μM) for 72 h. Statistical analysis was done by one-way ANOVA; *p < 0.05, **p < 0.01, ***p < 0.001, ^#^*p* < 0.0001, compared to the vehicle-treated control (CTR). Data represent the mean + SEM of ≥3 independent experiments with three replicates each. (D) SC_50_ values of **4b**, **5c**, **6b**, **6c**, **7b,** and **7c** were calculated by curve fitting to normalized response. Data represent ≥3 independent experiments with three replicates each. (E) MTT assay of K562 cells treated with **1a**–**c**, **2a**–**c**, **3a**–**c**, **4a**–**c**, **5a**–**c**, **6a**–**c**, **7a** (telmi), **7b** (telmi-ester), and **7c** (telmi-amide) at 10 μm for 72 h. Significant reductions of metabolic activity relative to the vehicle-treated control (CTR) are indicated. Statistical analysis was done with the Student’s paired *t*-test; *p < 0.05, **p < 0.01, ***p < 0.001. Data represent the mean + SEM of ≥3 independent experiments with three replicates each.

First, the compounds (10 μM) were applied alone to evaluate their effects per se. In this setting, only the phenyl derivatives **1b** (*o*-CO_2_CH_3_) and **2b** (*m*-CO_2_CH_3_) as well as the biphenyl derivatives **4b** (*o*-CO_2_CH_3_), **5b** (*m*-CO_2_CH_3_), and **5c** (*o*-CONH_2_) repressed the metabolic activity of K562-R cells below 70% (marked in red, Figure 2A). Additionally, **1c** (*p*-CONH_2_) and **6b** (*o*-CO_2_CH_3_) reduced the viability to about 75–80% and can also be considered as slightly active. Telmisartan (**7a**) and its methyl ester (**7b**) and carboxamide (**7c**) derivatives were inactive.

Imatinib (1 μM) reduced the metabolic activity of K562-R cells to 73% (control (CTR), Figure 2B) compared to the 100% solvent control. This effect could not be increased upon coapplication of the acids **1a** to **6a** as well as the benzamides **1c**, **2c**, and **3c**. The methyl esters **1b**, **2b**, **3b**, **5b**, and telmisartan (**7a**), however, significantly enhanced the efficacy of imatinib, reducing the viability of K562-R cells to 34–58%. The most effective compounds were those bearing the biphenyl moiety. The methyl esters **4b**, **6b**, and **7b** (telmi-ester) and the carboxamide derivatives **5c**, **6c**, and **7c** (telmi-amide) strongly sensitized the cells to imatinib so that the metabolic cell activity could be reduced to 8–24% (Figure 2B). It is noteworthy that telmisartan (**7a**) was the only COOH derivative that marginally enhanced imatinib treatment (Figure 2B,C).

The high relevance of the biphenyl moiety (compared to the phenyl residue) was confirmed by the concentration-dependent analysis (0.5–8 μM) of the most potent chemosensitizers (Figure 2B, marked in blue). The benzoic esters (**1b**, **2b**, and **3b**) at a concentration of 8 μM reduced the metabolic activity to 44–57% when combined with 1 μM of imatinib. In contrast, the biphenyl derivatives achieved this level of metabolic repression at significantly lower concentrations of 2–3 μM (Figure 2C).

Interestingly, telmisartan (**7a**) acted as a chemosensitizer, but was unable to enhance the antimetabolic effect of imatinib to more than 50%. At 8 μM, 55% of the K562-R cells remained unchanged. In contrast, telmi-ester (**7b**) and telmi-amide (**7c**) strongly sensitized the cells to imatinib therapy.

From the concentration-dependent evaluation of the chemosensitizing effects, SC_50_ values (drug concentration eliciting 50% of the maximum sensitizing effect) were calculated, which amounted to 1.2–2.2 μM (Figure 2D) for **4b**, **5c**, **6b**, **6c**, and telmi-amide (**7c**). Notably, the most potent cell death modulator, telmi-ester (**7b**), exhibited the lowest SC_50_ value of approximately 110 nM.

Selectivity for therapy-resistant K562-R cells can be assessed by comparing the results with TKI-sensitive K562 cells. Antimetabolic effects in K562 cells were only observed for **4b**, **5b**, **5c**, and **6b** at the highest applied concentration of 10 μM (Figure 2E). However, they did not reduce the metabolic activity below 65%. It can therefore be stated that the compounds possess a high selectivity toward TKI-resistant K562-R CML cells.

Based on these results, **1b**, **2b**, **3b**, **4b**, **5c**, **6b**, **6c**, as well as **7a**–**c** were selected for comprehensive studies on the mode of action.

### 2.3 Designed Chemosensitizers Repress the CSC-Defining Side Population in TKI-Resistant CML Cells

In previous studies, we demonstrated that the side population (SP) phenotype in cancer cell lines correlates with the persistence of a therapy-resistant CSC pool.^26,27^ These CSCs overexpress drug transporters (*e.g.,* ABCB1, ABCG2, and ABCC1), resulting in high drug resistance. Furthermore, elevated STAT5 signaling triggers CSC persistence and drives not only the relapse of CML, but also that of various solid tumors.^18^

**Figure 3:**
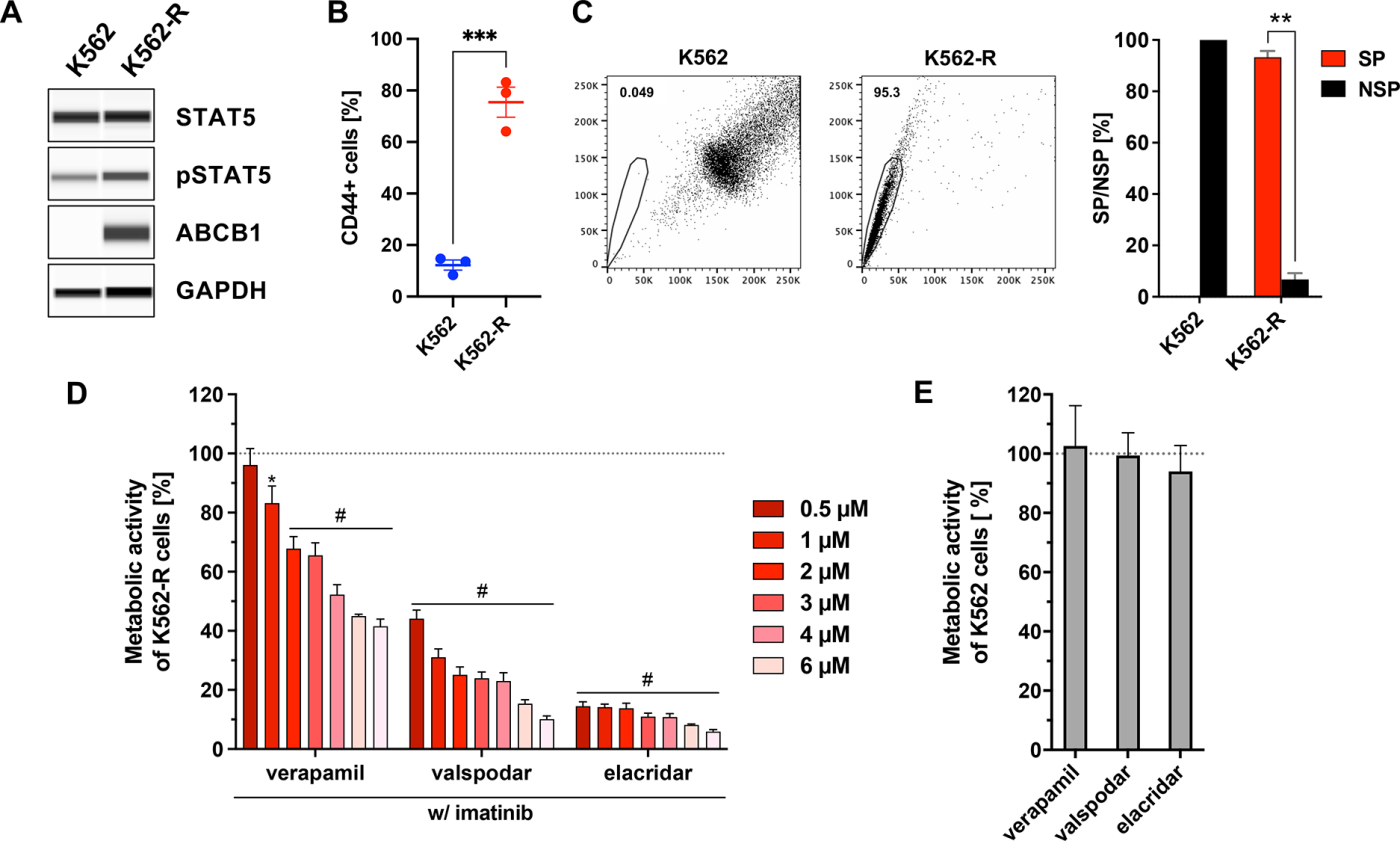
TKI resistance correlates with CSC features in K562-R cells. (A) Jess Simple Western immunoassays to determine the protein expression of STAT5 (∼90 kDa), pSTAT5-tyr694/699 (∼90 kDa), and ABCB1 (∼180 kDa) in K562 and K562-R cells. GAPDH (∼40 kDa) served as loading control. (B) Flow cytometry analysis of CD44 expression in K562 and K562-R cells. Shown is the proportion of CD44^+^ cells [%]. Data represent the mean ∓ SEM of 3 independent experiments. Statistical analysis was done with the Student’s unpaired *t*-test; ***p < 0.001. (C) K562 and K562-R cells were stained using DyeCycle Violet (DCV) and analyzed by flow cytometry. SP subsets are indicated by polygonal gates and the percentage of cells within these gates is given. Quantification of SP and non-side population (NSP) subsets in K562 and K562-R cells. Data represent the mean + SEM of ≥3 independent experiments. Statistical analysis was done with the Student’s paired *t*-test; **p < 0.01 (D) Concentration-dependent impact of verapamil, valspodar, and elacridar on the metabolic activity of K562-R cells, cotreated with imatinib (1 μM) for 72 h. Statistical analysis was done by one-way ANOVA; *p < 0.05, ^#^*p* < 0.0001, compared to the vehicle-treated control (DMSO + imatinib (1 μM)). Data represent the mean + SEM of ≥3 independent MTT experiments with three replicates each. (E) Metabolic activity of K562 cells after incubation with verapamil, valspodar, and elacridar at 10 μM for 72 h. The vehicle-treated control (DMSO) served as a reference (100%). Data represent the mean + SEM of ≥3 independent MTT experiments with three replicates each.

In particular, activated pSTAT5 binds directly to the promoter region of the ABCB1 gene and induces its expression. Accordingly, increased phosphorylation of STAT5 at Tyr694/699 (indicating activation) and upregulation of ABCB1 protein expression was observed in K562-R cells compared to the corresponding K562 parental cell line (Figure 3A). Notably, K562-R cells exhibited a significantly increased expression of the CSC marker CD44,^28^ further reinforcing the notion that the side population (SP) phenotype is indicative of stem cell-like properties (Figure 3B). The higher density of drug transporters increases the capacity for drug efflux, which is reflected in a significantly expanded SP compared to the non-side population (NSP) in K562-R cells (Figure 3C).

The involvement of the ABCB1 transporter in the resistance of K562-R cells to imatinib was investigated in a cytotoxicity test (MTT assay) by simultaneous application of the drug (1 μM) with selected commercially available ABC transporter inhibitors (verapamil, valspodar, and elacridar; conc. 10 to 0.5 μM) over 72 h (Figure 3D).

Verapamil (first generation ABCB1 inhibitor) enabled an imatinib-induced reduction of the metabolic activity in K562-R cells comparable to the phenyl derivatives **1b**, **2b**, **3b**. Valspodar (second generation ABCB1 inhibitor), on the other hand, sensitized the cells similarly to the derivatives **4b**, **5c**, **6b**, **6c** from the biphenyl series. Finally, the chemosensitizing efficacy of the third generation ABCB1 inhibitor elacridar was comparable to telmi-ester (**7b**) (Figure 3D). It is noteworthy that verapamil, valspodar, and elacridar applied alone did not affect the viability of the parental cell line K562 (Figure 3E) as well as the K562-R subline (data not shown) at the investigated concentration of 10 μM.

The new chemosensitizers may directly inhibit the ABCB1 transporter as part of their mode of action. Yet, the efficiency of the transporter can also be impaired indirectly via the STAT5 pathway. Previous studies revealed that suppression of STAT5 signaling plays a central role in the molecular mechanism by which related telmisartan-derived chemosensitizers increase the sensitivity of therapy-resistant CML cells to imatinib.^14−17^

**Figure 4:**
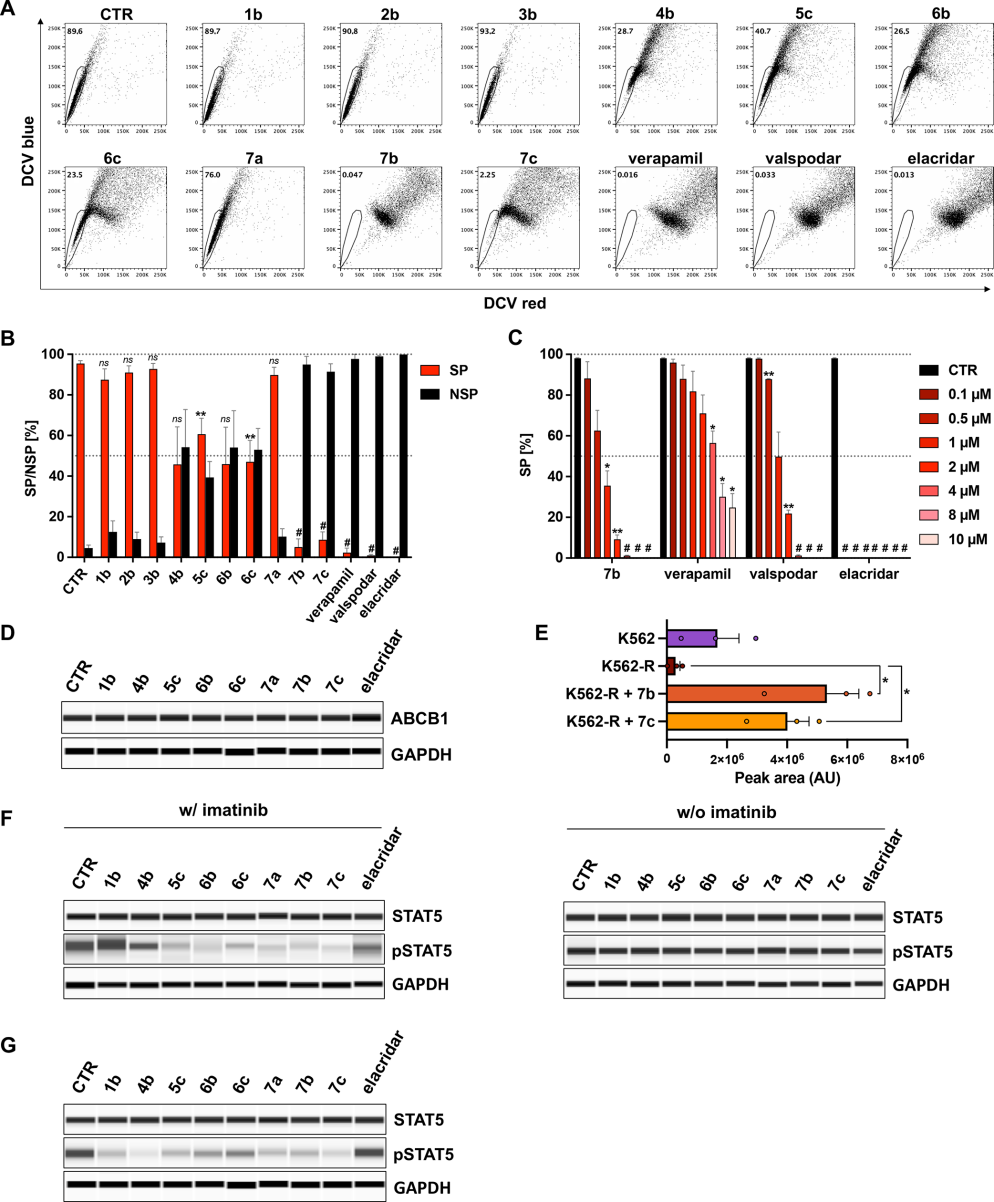
Telmisartan derivatives inhibit the SP phenotype and repress the phosphorylation status of STAT5 in K562-R cells. (A) K562-R cells were stained with DCV at 10 μM and concurrently incubated with 10 μM of **1b**, **2b**, **3b**, **4b**, **5c**, **6b**, **6c**, **7a**–**c**, valspodar, elacridar, or 160 μM of verapamil for 90 min. The subsequent analysis was carried out by flow cytometry. SP subsets are indicated by polygonal gates, and the percentage of cells within these gates is given. (B) Quantification of SP and NSP subsets of ≥3 independent experiments, shown as the mean + SEM. Statistical analysis was done with the Student’s paired *t*-test; **p < 0.01, ^#^*p* < 0.001, compared to the proportion of the SP in vehicle-treated cells (CTR, DMSO) (C) K562-R cells were stained with DCV and concurrently incubated with indicated concentrations of **7b** (telmi-ester), verapamil, valspodar, and elacridar. SP subsets were subsequently analyzed by flow cytometry. Shown is the mean + SEM of ≥3 independent experiments. Statistical analysis was done by one-way ANOVA; *p < 0.05, **p < 0.01, ^#^*p* < 0.0001, compared to the proportion of the SP in vehicle-treated cells (CTR) (D) Jess Simple Western immunoassays determining the expression of ABCB1 (∼180 kDa). K562-R cells were incubated with vehicle (CTR, DMSO) or the compounds **1b**, **4b**, **5c**, **6b**, **6c**, **7a**–**c**, and elacridar at 4 μM for 72 h, respectively. GAPDH (∼40 kDa) served as loading control. (E) K562 and K562-R cells were preincubated for 1 h with 10 μM **7a** or **7b** and subsequently treated with 1 μM of imatinib for 2 h. Cell pellets were harvested, washed, and analyzed by U(H)PLC-SIM-MS to detect intracellular imatinib accumulation. Shown is the mean + SEM of 3 independent experiments. Statistical analysis was done with the Student’s paired *t*-test; *p < 0.05. (F) Jess Simple Western immunoassays determining the expression of STAT5 (∼90 kDa) and pSTAT5 (∼90 kDa). K562-R cells were treated with vehicle (CTR, DMSO) or 4 μM of the compounds **1b**, **4b**, **5c**, **6b**, **6c**, **7a**–**c**, and elacridar, either alone (right blot) or in combination with 1 μM of imatinib (left blot) for 6 h, respectively. GAPDH (∼40 kDa) served as loading control. (G) Jess Simple Western immunoassays determining the expression of STAT5 (∼90 kDa) and pSTAT5 (∼90 kDa). K562-R cells were treated with vehicle (CTR, DMSO) or 4 μM of the compounds **1b**, **4b**, **5c**, **6b**, **6c**, **7a**–**c**, and elacridar for 72 h. GAPDH (∼40 kDa) served as loading control.

Therefore, the effect of **1b**, **2b**, **3b**, **4b**, **5c**, **6b**, **6c**, **7a** (telmi), **7b** (telmi-ester), and **7c** (telmi-amide) on the ABCB1 transporter in K562-R cells was evaluated in more detail (Figure 4). The benzoic esters **1b**, **2b**, and **3b** as well as **7a** had no significant impact on the SP at a concentration of 10 μM. The amount was comparable to that of the control (CTR, Figure 4B). The biphenyl bearing compounds **4b**, **5c**, **6b**, and **6c** efficiently reduced the ABCB1 activity, as reflected by the repressed SP phenotype. The relation of SP and NSP was about 50% (Figure 4B). Finally, telmi-ester (**7b**) and telmi-amide (**7c**) completely repressed the SP phenotype in K562-R cells, comparable to existing transporter inhibitors (Figure 4A,B).

Concentration-dependent analysis showed that the efficacy of **7b** in inhibiting the drug transporter was similar to valspodar (Figures 4C and S61). Worth mentioning is the high activity of elacridar that efficiently repressed the SP at a concentration as low as 0.1 μM (Figures 4C and S61).

To evaluate a possible influence on the transporter expression, K562-R cells were incubated with vehicle (CTR, DMSO) or the compounds **1b**, **4b**, **5c**, **6b**, **6c**, **7a**–**c**, and elacridar at 4 μM for 72 h, respectively. As proven by immunoassays, none of the compounds abrogated the ABCB1 protein expression per se (Figure 4D). These results support the hypothesis that the developed derivatives inhibit the increased activity of the drug transporter in TKI-resistant CML cells by direct functional inhibition of the efflux pump and thus enable the targeting of the CSC-defining SP. Consistently, ultrahigh-performance liquid chromatography-selected ion monitoring-mass spectrometry (U(H)PLC-SIM-MS) analysis revealed that the intracellular accumulation of imatinib—significantly reduced in K562-R cells compared to K562 cells—was notably restored in K562-R cells treated with telmi-ester (**7b**) and telmi-amide (**7c**) (Figure 4E).

In the next step, the effects of the compounds (4 μM) on STAT5 in K562-R cells were examined. After 6 h of incubation, the phosphorylation status of STAT5 remained nearly unchanged (Figure 4F, right panel) compared to the control. However, incubation for 72 h showed another picture. Elacridar, which suppressed TKI resistance (Figure 3D) and efficiently abolished the SP phenotype (Figure 4A,B), did not influence the phosphorylation of STAT5 per se, even after 72 h (Figure 4G). In contrast, **1b, 4b, 5c, 7a–c** and, to a lesser extent, **6b** and **6c** prevented the activation of STAT5.

Interestingly, coapplication of imatinib (1 μM) improved the effects of **5c**, **6b**, **6c**, **7a**–**c**. Already after 6 h, a marked repression of the STAT5 phosphorylation was observed (Figure 4F, left panel). In contrast, elacridar as well as **1b** and **4b** hardly triggered any change in the expression of pSTAT5.

In summary, these results suggest that the developed chemosensitizers target critical stemness factors, since they inhibit drug transporter activity and impair STAT5 signaling via independent mechanisms.

### 2.4 Telmisartan-Derived Chemosensitizers Suppress Colony Formation of Primary CML Cells and Inhibit Tumor Growth In Vivo

**Figure 5:**
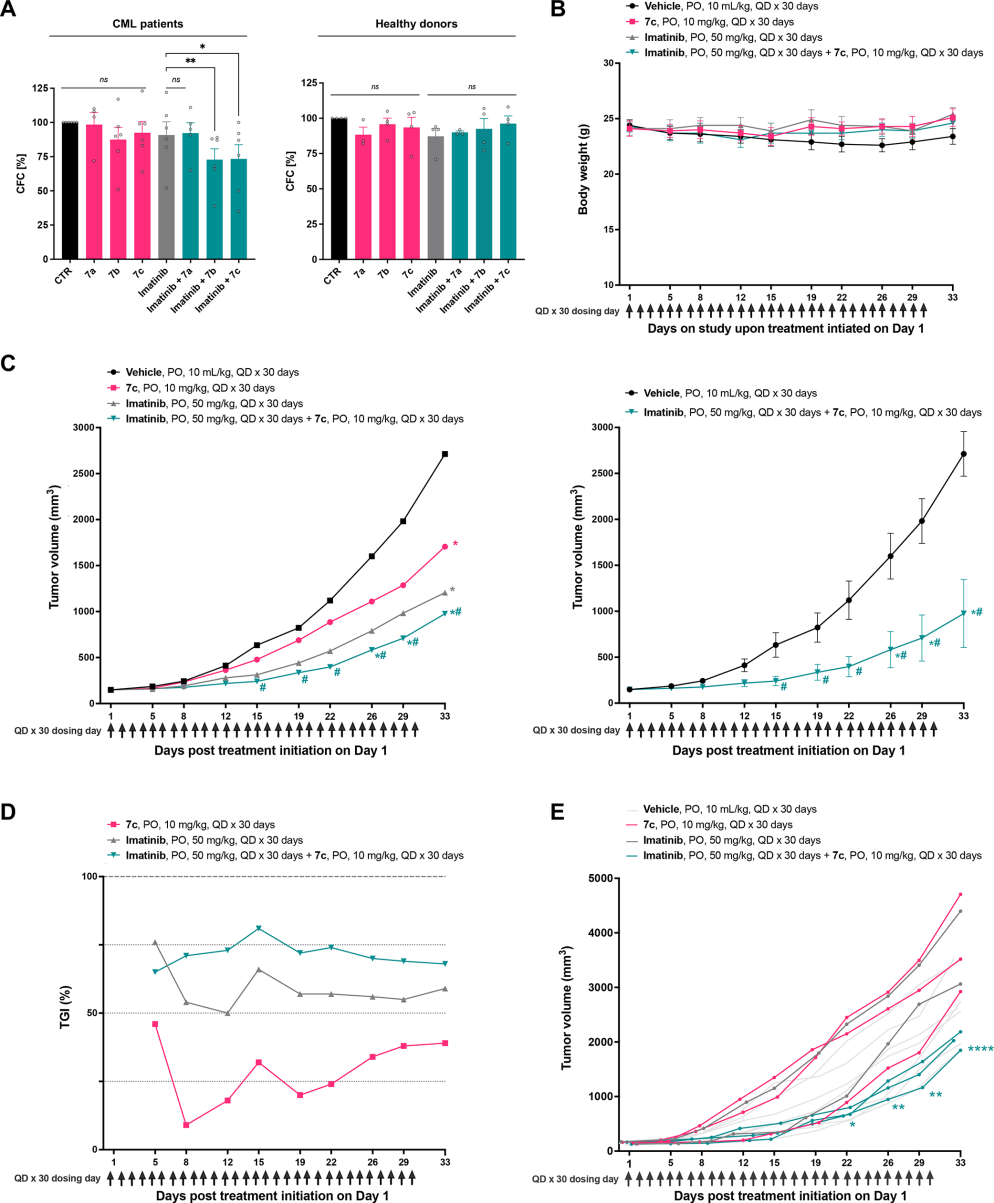
Telmisartan-derived chemosensitizers suppress colony formation in primary CML cells and inhibit tumor growth in vivo. (A) Numbers of CFCs of human primary CML patient samples (left panel) or healthy donors (right panel) treated with either 0.1 μM imatinib or 10 μM **7a**, **7b**, **7c**, or in combination. Values were normalized to untreated control cultures and are shown as mean + SEM. Statistical analysis was done with Student’s paired *t*-test; *P < 0.05, **P < 0.01. (B–E) Efficacy evaluation of **7c** and imatinib in a human CML K562-R xenograft model in female NOD/SCID mice subcutaneously (SC) implanted with viable human CML K562-R cells at 1 × 10^7^ cells/mouse (0.2 mL/animal with 50% high concentration Matrigel) to the left flank. Treatment was initiated on day 1, when the mean tumor volume reached 148–149 mm^3^, which was 14 days post tumor cell implantation. The tested compounds **7c** (at 10 mg/kg) and imatinib (at 50 mg/kg) were orally (PO) administered once daily for 30 consecutive days (QD × 30 days) with a total of 30 dose administrations as monotherapy and combination therapy. (B) Body weight of animals (g) over time (day). Error bars are SEM values. Two-way ANOVA followed by Tukey’s multiple comparison test was conducted for statistical analysis. No significant differences in body weight were observed. (C) Tumor volume (mm^3^) over time (day). Tumor growth, related to tumor volume by length × (width)^2^ × 0.5, was measured twice weekly from day 1 (treatment initiation) until the study end date (day 33). Two-way ANOVA followed by Tukey’s multiple comparison test was conducted for statistical analysis; *p < 0.05 (reduction in mean tumor volume compared to vehicle control). # signifies the achievement of significant antitumor activity in a group, as defined by a %T/C (Treatment/Control) value of ≤42%. According to NCI (National Cancer Institute) criteria, a %T/C value ≤42% relative to the vehicle control group denotes substantial antitumor efficacy.^34^ The left panel comprises all treatment groups for comparison without error bars to enhance clarity. The right panel focuses on the groups demonstrating significant antitumor activity (%T/C value ≤42%), specifically highlighting the combination therapy of imatinib and **7c**, with error bars representing + SEM values. (D) Tumor growth inhibition (TGI) of **7c**, imatinib, and the combination therapy in the human CML K562-R xenograft model. Shown is percent of tumor growth inhibition (%TGI) over time (day). (E) Tumor growth, related to tumor volume by length × (width)^2^ × 0.5, in a selected cohort of mice exhibiting tumor progression (*n* = 15; see Figure S62). Two-way ANOVA followed by Tukey’s multiple comparison test was conducted for statistical analysis; *p < 0.05, **p < 0.01, ****p < 0.0001 (reduction in mean tumor volume compared to imatinib monotherapy).

Next, we investigated the effects of telmisartan (**7a**), telmi-ester (**7b**), and telmi-amide (**7c**) on hematopoietic progenitor cell and cellular stem cell accumulation by colony forming cell (CFC) analysis using samples from treatment-naïve primary human CML patients (*n* = 6). As mentioned above, **7b** and **7c** inhibited both critical stemness factors STAT5 and ABCB1. These compounds also significantly reduced the number of colonies when administered together with 1 μM of imatinib once at the beginning of the 14 day assay. Telmisartan **7a** was virtually ineffective in this setting (Figure 5A, left panel).

Importantly, none of the tested molecules affected the growth of colonies derived from healthy donors when administered alone or in combination with imatinib (Figure 5A, right panel). These results highlight the potential of the telmisartan derivatives to selectively target leukemic CSCs.

With the aim of subjecting the compounds to an in vivo efficacy study, the selected derivatives **5c**, **7b**, and **7c** were submitted to absorption, distribution, metabolism, and excretion (ADME) assays. Evaluation of the physicochemical properties and pharmacokinetics is crucial to assess their in vivo efficacy and safety and to identify the most suitable compound. The results of the ADME studies are summarized in Table 1.

**Table 1:** Results of ADME and Physicochemical Tests for Compounds **5c**, **7b**, and **7c**^a^

	solubility [μM]	Caco2 permeability P_app_			metabolic stability Human		metabolic stability Mouse		hepatocytic stability human		hepatocytic stability mouse	
		A–B [10 ^–6^ cms ^–1^]	B–A [10 ^–6^ cms ^–1^]	efflux ratio	[%]	*t*_1/2_ [min]	[%]	*t*_1/2_ [min]	[μL/min/10^6^ cells]	*t*_1/2_ [min]	[μL/min/10^6^ cells]	*t*_1/2_ [min]
**5c**	<20.0	24.8	30.6	1.23	15.7	11.9	0.3	2.8	49.0	28.3	280	5.0
**7b**	<6.5	0.1	0.2	1.24	26.3	16.2	3.0	6.0	75.2	18.4	101	13.8
**7c**	<6.5	24.3	30.8	1.27	58.1	45.1	13.3	8.8	13.8	100	30.9	44.9

a Solubility: Turbidimetric aqueous solubility estimated precipitation range in μM; Caco2 Permeability: Apparent permeability coefficient P_app_ apical to basolateral (A–B) and basolateral to apical (B–A) at pH 7.4 in 10^–6^ cms^–1^, and efflux ratio calculated as P_app_ B–A/P_app_ A–B; Metabolic Stability (Human and Mouse): % of compound remaining after 30 min in human liver microsomes (HLM) and mouse liver microsomes (MLM). Half-life (*t*_1/2_) [min] of compounds in HLM and MLM. Hepatocytic Stability (Human and Mouse): Intrinsic metabolic clearance (CL_int_) rate after 60 min in human hepatocytes (HH) and mouse hepatocytes (MH), expressed in microliters per min per 10^6^ cells. Half-life (*t*_1/2_) [min] of compounds in HH and MH.

The estimated solubility, determined by turbidimetry, was <20 μM for **5c** and <6.5 μM for **7b** and **7c**, which is consistent with published solubility data for the FDA-approved drug telmisartan (reported aqueous solubility of **7a**: 9.9 μg/mL = 19.2 μM^29^).

The Caco2 permeability assay showed a more differentiated outcome among the compounds. Notably, **5c** (24.8 and 30.6 × 10^–6^ cms^–1^) and **7c** (24.3 and 30.8 × 10^–6^ cms^–1^) exhibited an apparent permeability coefficient (P_app_) comparable to that of telmisartan (**7a**: 29 × 10^–6^ cms^–1^),^30^ while the one of **7b** (0.1 and 0.2 × 10^–6^ cms^–1^) was much lower, indicating less in vivo absorption. The efflux ratio, calculated as the ratio of basolateral to apical permeability (B–A) over apical to basolateral permeability (A–B) for each compound, was approximately 1.2. This suggests that none of the compounds is significantly affected by active efflux mechanisms.

The metabolic stability of the compounds was evaluated using human and mouse liver microsomes (HLM and MLM). Telmi-amide (**7c**) exhibited the highest stability in both HLM and MLM, with 58.1% of the compound remaining unmetabolized in HLM and 13.3% in MLM after 30 min (HLM: **5c** 15.7%, **7b** 26.3%; MLM: **5c** 0.3%, **7b** 3%). In accordance, **7c** also showed the longest half-life in both HLM (*t*_1/2_ = 45.1 min) and MLM (*t*_1/2_ = 8.8 min). For **5c** a half-live of *t*_1/2_ = 11.9 and 2.8 min, and for **7b** a half-live of *t*_1/2_ = 16.2 and 6.0 min was measured.

The stability was further investigated in human and mouse hepatocytes (HH and MH). Telmi-amide (**7c**) showed the lowest intrinsic metabolic clearance (CL_int_) in HH with 13.8 μL/min/10^6^ cells (*t*_1/2_ = 100 min), and in MH with 30.9 μL/min/10^6^ cells (*t*_1/2_ = 44.9 min), indicating medium clearance in HH and high clearance in MH.

For comparison: telmisartan has a CL_int_ of about 23 μL/min/10^6^ cells in rat hepatocytes, which is in the medium clearance range for this species.^30^ The values for **5c** are 49.0 μL/min/10^6^ cells (*t*_1/2_ = 28.3 min) in HH, and 280 μL/min/10^6^ cells (*t*_1/2_ = 5.0 min) in MH. Those of **7b** are 75.2 μL/min/10^6^ cells (*t*_1/2_ = 18.4 min) in HH, and 101 μL/min/10^6^ cells (*t*_1/2_ = 13.8 min) in MH.

Due to the higher predicted in vivo absorption and stability as well as the excellent in vitro results, telmi-amide (**7c**) was selected for in vivo efficacy experiments using the human K562-R xenograft model in female NOD/SCID mice (*n* = 7 animals per group, *n* = 28 animals in total; refer to Study Design in Table S1).

The in vivo dose for imatinib was chosen based on published combination studies reporting lower doses such as 50 mg/kg daily in mouse models,^31^ as opposed to the 100 mg/kg twice daily, which closely mimic the human pharmacokinetic profile of a 400 mg daily dose.^32^ For compound **7c**, the in vivo dose was selected according to preclinical data of the related telmisartan, which has a similar ADME/PK profile. Varying doses have been reported in studies depending on the context and we decided on the similar starting dose of 10 mg/kg daily, with the flexibility to adjust it according to the experimental results.^33^ Telmi-amide (**7c**; 10 mg/kg) and imatinib (50 mg/kg) were administered orally (PO) once daily for 30 consecutive days as monotherapy or combination therapy (once a day (QD) × 30 days). Both compounds were well tolerated. No significant toxic effects appeared in the treatment groups, which would be reflected in weight loss compared to the vehicle control (Figures 5B, S62A, and Table S1).

Imatinib and **7c** significantly reduced tumor volume by day 33 (Figures 5C, S62B, and Table S1) with %T/C (treatment/control) values of 45% and 63%, respectively. However, solely the combination therapy reduced tumor volume from day 26 and achieved a significant antitumor activity from day 15 on, reaching a T/C = 36% at the end of the experiment. According to NCI (National Cancer Institute) criteria, a %T/C value ≤ 42% relative to the vehicle control group denotes substantial antitumor efficacy.^34^

At the same time, the combination therapy group caused the strongest tumor growth inhibition (TGI) (Figure 5D). The administration of imatinib, **7c**, or their combination resulted in complete inhibition of tumor growth in a subset of mice (Figure S62C and Table S1). Within the cohort of mice exhibiting tumor progression, the combination therapy of **7c** with imatinib significantly (*p* < 0.001) decreased tumor volumes compared to the imatinib monotherapy (Figures 5E and S62D).

In summary, the results presented here provide compelling evidence that the developed telmisartan-derived chemosensitizers effectively inhibit colony formation in primary CML cells and reduce tumor growth in the K562-R xenograft model in vivo, while showing no detectable toxic side effects. This is particularly remarkable given that the dose used is only one-fifth of that of imatinib. Thus, higher dosage could potentially enhance the efficacy of compound **7c** further. Notably, the antitumor efficacy observed in vivo was achieved by oral administration, a route of administration that is of significant clinical importance due to its potential for improved patient compliance and convenience.

### 2.5 Telmisartan-Derived Chemosensitizers Efficiently Target the CSC Pool of Solid Cancers

In recent studies, we described the side population (SP) phenotype as a marker that defines a heterogeneous cellular compartment with stem cell-characteristics in various cancer cell lines derived from solid tumors, such as ovarian cancer, breast cancer, and lung cancer.^26,27^ Following our excellent results with leukemic cells, we investigated whether the synthesized compounds also target the CSC pool in solid tumors. Accordingly, the CSC-enriched, paclitaxel-resistant ovarian cancer cell lines A2780 V SP and IGROV-1 SP (both ABCB1+^35^), the docetaxel-resistant prostate cancer cell lines PC3-DR and DU145-DR (both ABCB1+; Figure S63A), the breast cancer cell line MCF7 (ABCG2+; Figure S63B), and the lung cancer cell line A549 (ABCC1+; Figure S63B) were used to analyze the effect of the most promising compounds **5c**, **6c**, and **7a**–**c** on the SP phenotype at a concentration of 10 μM.

**Figure 6:**
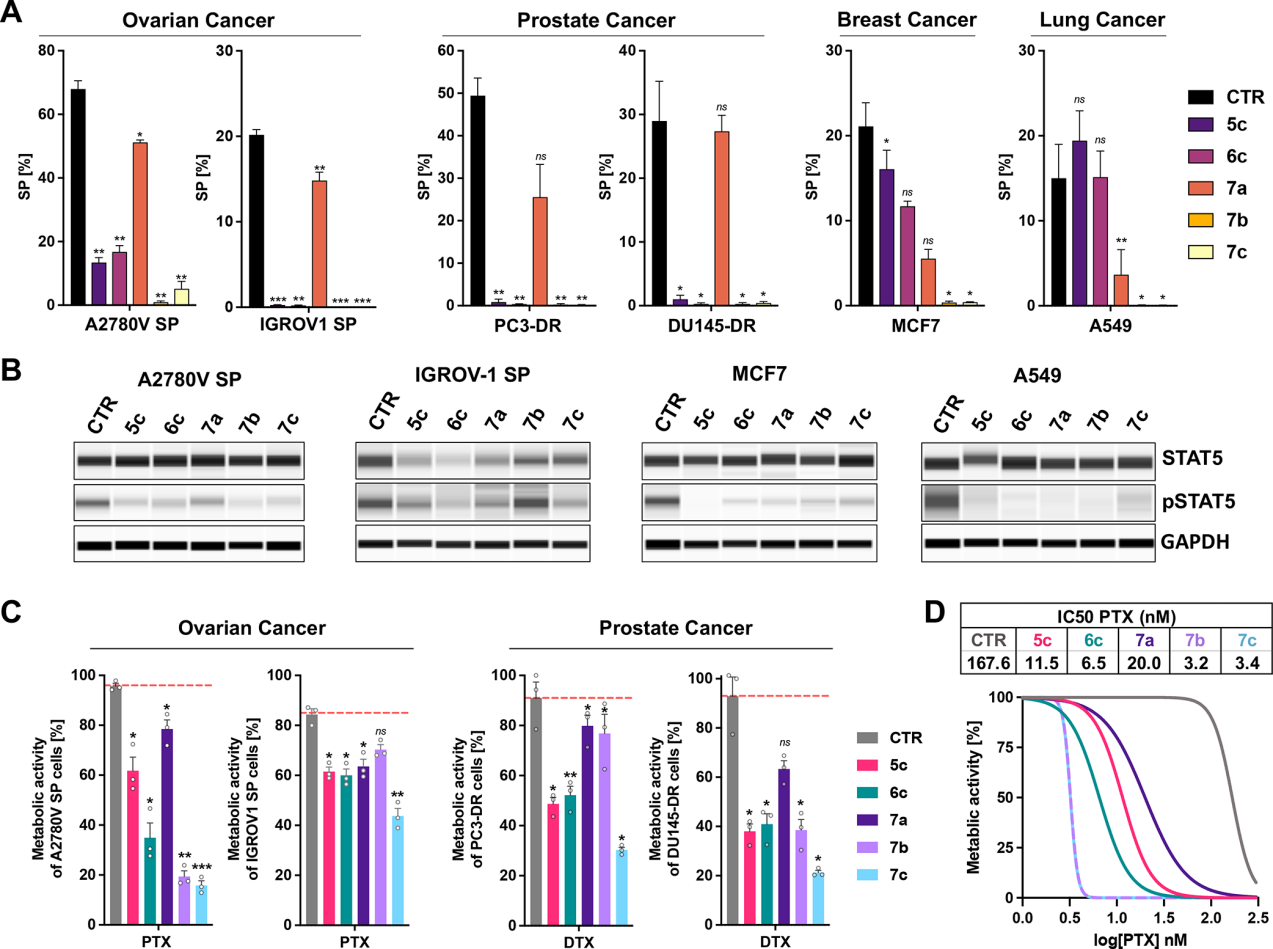
The developed telmisartan derivatives target the CSC pool of solid cancers. (A) Quantification of the SP subsets detected in indicated ovarian cancer, prostate cancer, breast cancer, and lung cancer cell lines. Shown is the mean + SEM of ≥3 independent experiments. Statistical analysis was done with the Student’s paired *t*-test; **p < 0.01, ^#^*p* < 0.0001, compared to the proportion of the SP in vehicle-treated (CTR) cells. (B) Jess Simple Western immunoassays determining the expression of STAT5 (∼90 kDa) and pSTAT5 (∼90 kDa). A2780 V SP, IGROV-1 SP, MCF7, and A549 cells were treated with vehicle (CTR, DMSO) or 10 μM of the compounds **5c**, **6c**, **7a**, **7b**, and **7c** for 72 h, respectively. GAPDH (∼40 kDa) served as loading control. (C) A2780 V SP and IGROV-1 SP cells were treated with 10 μM of **5c, 6c, 7a**, **7b**, or **7c** in combination with 10 nM of paclitaxel (PTX). PC3-DR and DU145-DR cells were treated with 10 μM of **5c, 6c, 7a**, **7b**, or **7c** in combination with 12.5 nM of docetaxel (DTX). CTR represents the vehicle-treated (DMSO) control. After 72 h, the metabolic activity of the cells was determined by a modified MTT assay. Data represent the mean + SEM of *n* = 3 independent experiments with three replicates each. Statistical analysis was done with the Student’s paired *t*-test; *p < 0.05, **p < 0.01, ***p < 0.001, compared to cells treated with PTX or DTX alone (CTR). (D) A2780 V SP cells were treated with 10 μM of **5c, 6c, 7a**, **7b**, or **7c** in combination with indicated concentrations of paclitaxel (PTX; 0–300 nM). IC_50_ values of PTX were calculated by curve fitting to normalized response. Data represent *n* = 3 independent experiments with three replicates each.

Telmisartan (**7a**) significantly inhibited the SP phenotype of ABCG2+ MCF-7 breast cancer and ABCC1+ A549 lung cancer cells, but only modestly affected ABCB1+ A2780 V SP and IGROV-1 SP ovarian and PC3-DR and DU145-DR prostate cancer cells (Figures 6A and S64). The activity of **7a** on ABCB1+ K562-R cells was similarly limited (Figure 4A,B). In contrast, **5c** and **6c** were nearly inactive in MCF7 and A549 cells, but effectively targeted the SP phenotype in A2780 V SP and IGROV-1 SP as well as PC3-DR and DU145-DR cells (Figures 6A and S64). This suggests the specific inhibition of ABCG2/ABCC1 by **7a** and ABCB1 by **5c** and **6c**.

Derivatization of telmisartan strongly increased the effects. Telmi-ester (**7b**) and telmi-amide (**7c**) eliminated the SP phenotype in all examined cell lines, demonstrating their broad-spectrum efficacy against ABCB1, ABCG2, and ABCC1 transporters.

The impact of STAT5 activation on the mode of action was investigated in selected solid tumor cell lines as well. After incubation for 72 h at a concentration of 10 μM, **5c**, **6c**, **7a**–**c** efficiently repressed the phosphorylation status of STAT5 in A2780 V SP, IGROV-1 SP, MCF7, and A549 cells. The STAT5 protein expression itself was not altered in A2780 V SP, MCF7, and A549 cells. However, in IGROV1 SP cells **5c**, **6c**, and **7a** significantly suppressed STAT5 (Figure 6B).

The chemosensitizing efficacy was investigated in the ovarian and prostate cancer cell lines using the above-described metabolic activity assay. Paclitaxel (PTX) served as a reference (CTR) in A2780 V SP and IGROV-1 SP cells and docetaxel (DTX) in PC3-DR and D145-DR cells. At the used concentrations, PTX (10 nM) and DTX (12.5 nM) were inactive (metabolic activity of treated cells >80% compared to solvent control).

Incubation with **5c**, **6c**, **7a**–**c** particularly sensitized the therapy-resistant A2780 V SP ovarian cancer cells, which possess a high proportion of CSCs (SP > 60%), to PTX therapy (Figure 6C). Telmisartan (**7a**), which repressed the SP phenotype of A2780 V SP cells to a lesser extent than the other compounds (Figure 6A,B), was also the least active chemosensitizer (Figure 6C,D).

For a better comparison, the IC_50_ value (drug concentration causing 50% of the maximum inhibition) of PTX alone and in the presence of 10 μM of the add-ons was determined in A2780 V SP cells. The antimetabolic activity of PTX (IC_50_ (PTX) = 167.6 nM) strongly increased upon coapplication of **7a** (IC_50_ (PTX) = 20.0 nM), **5c** (IC_50_ (PTX) = 11.5 nM), and **6c** (IC_50_ (PTX) = 6.5 nM). The most active compounds were again **7b** (IC_50_ (PTX) = 3.2 nM) and **7c** (IC_50_ (PTX) = 3.4 nM).

The combination therapy was not as effective in IGROV-1 SP cells. **5c**, **6c**, and **7a** increased the antimetabolic effect of PTX, while **7b** was inactive, although it efficiently inhibited the ABCB1 activity in this cell line (Figure 6A). It practically did not suppress pSTAT5 (Figure 6B), which could explain the limited chemosensitizing effect in these cells. Telmi-amide (**7c**) enhanced the PTX activity and reduced the viability of the cells to about 45%.

The resistance of the PC3-DR (SP ∼ 50%) and D145-DR (SP ∼ 30%) prostate cancer cell lines could also be circumvented. The most active chemosensitizer was **7c**, followed by **5c** and **6c**. Telmisartan (**7a**) and its ester **7b**, only marginally increased the antimetabolic activity of DTX in PC3-DR cells. In D145-DR cells, **7b** produced the same effects as **5c** and **6c**, whereas **7a** did not significantly reduce the metabolic activity in combination with DTX.

These results confirm that the agents developed can serve as tumor-independent add-on therapeutics to tackle therapy-resistant CSCs.

## 3 Discussion

In the present study, we developed new derivatives of telmisartan to create potent, nontoxic chemosensitizers that specifically target and eradicate therapy-resistant CSCs. The compounds were intensively investigated to clarify whether they inhibit the critical factors that contribute to CSC persistence: enhanced activity of drug transporters^10,36^ and hyperactivation of STAT5 signaling.^18^

To deepen our previous SAR studies^14−17^ about 4′-((2-propyl-1*H*-benzo[*d*]imidazole-1-yl)methyl)-[1,1′-biphenyl]-2-carboxylic acid and derivatives as chemosensitizers, the carboxylic group was exchanged for CONH_2_ and CO_2_CH_3_ and shifted from the ortho to the meta or para position. In addition, the importance of the biphenyl core in these new compounds was evaluated by exchange for a phenyl ring (→ 2-((2-propyl-1*H*-benzo[*d*]imidazole-1-yl)methyl)benzoic acid and –CO_2_CH_3_/-CONH_2_ derivatives). Furthermore, the 2-CO_2_CH_3_ and 2-CONH_2_ derivatives of telmisartan were included in this study.

Telmisartan (**7a**) displayed modest efficacy in sensitizing K562-R cells to imatinib therapy. This effect can be enhanced by esterification (**7b**) or amidation (**7c**) of the *o*-COOH group. Compounds **4a**–**c**, which represent the substructure of **7a**–**c**, were considerably less active chemosensitizer. Yet, modification of the biphenyl moiety’s substitution pattern, especially shifting the carboxamide from the ortho (**4c**) (see also^16^) to the meta (**5c**) or para position (**6c**), considerably increased the efficacy. In contrast, a comparable shift of the *o*-CO_2_CH_3_ group (**4b**) (see also^14^) resulted in a different order, as **4b** and **6b** (*p*-CO_2_CH_3_) were equally potent, whereas **5b** (*m*-CO_2_CH_3_) sensitized the cells to a lesser extent. Exchange of the biphenyl for a phenyl residue strongly reduced the efficacy. The related 2-((2-propyl-1*H*-benzo[*d*]imidazole-1-yl)methyl)benzoic acid derivatives sensitized the cells for imatinib treatment only comparably to telmisartan. Of all compounds tested, the derivatives telmi-amide (*o*-CONH_2_) and telmi-ester (*o*-CO_2_CH_3_) were the most efficient chemosensitizers, mediating high rates of imatinib-triggered cell death (>85% at 10 μM).

Most of the compounds are noncytotoxic in nonmalignant cells and specifically sensitize therapy-resistant cancer cells to treatment with commonly applied drugs. This finding represents a major advantage, as the clinical use of currently developed chemosensitizers is mostly limited by intolerable side effects observed in preclinical and clinical studies.^37^

The SP phenotype, which is defined by increased drug transporter activity (*e.g.,* ABCB1, ABCG2, ABCC1), correlates with the persistence of the therapy-resistant CSC pool in diverse therapy-resistant cancer cell lines.^26,27^ Along with this, we observed an increased ABCB1 drug transport capacity, which is reflected in a significantly expanded detection of the SP phenotype in TKI-resistant CML cell lines.

Weiss et al.^38^ investigated the interaction between ARBs and ABC transporters and found that telmisartan, to a lesser extent candesartan cilexetil, and only slightly irbesartan impeded ABCB1, whereas the other tested sartans were inactive. This is in accordance with our previous findings that telmisartan, candesartan cilexetil, and irbesartan function as chemosensitizers, while other ARBs do not exhibit sensitizing properties.^15^ The presence of a biphenyl-tetrazole or biphenyl-carboxylic acid moiety was found essential for AT1 inhibition and is conserved among the sartans that act as AT1 blockers, as opposed to the nonacidic functional groups we introduced, which facilitated a stronger chemosensitization in the first place.

While the esters of the phenyl series (**1b**, **2b**, **3b**) and telmisartan (**7a**) have limited influence on the capacity of the ABCB1 drug transporter, the methyl esters (**4b**, **6b**) and carboxamides (**5c**, **6c**) of the biphenyl series and telmisartan (**7b**, **7c**) efficiently inhibit ABCB1 efflux activity. Notably, telmi-ester (**7b**) showed an inhibitory effect similar to that of the second-generation drug transporter inhibitor valspodar. Concordantly with the inhibition of the drug transporter activity, treatment with telmi-ester (**7b**) and telmi-amide (**7c**) significantly increased the cellular accumulation of imatinib in K562-R cells.

This reduction in drug transporter activity by the new compounds also applies to a number of therapy-resistant solid tumor cell lines, including ovarian, prostate, breast, and lung cancers, underscoring their potentially broad clinical applicability. It is noteworthy that it is possible to achieve a particular selectivity toward certain drug transporters, which could significantly mitigate side effects in therapeutic applications to target drug resistance.

In addition to a direct interaction with the transporters, the effect of telmisartan derivatives may also be based, at least in part, on the inhibition of STAT5.^14−17^ Aberrant constitutive activation of STAT5 signaling promotes CSC maintenance and leads to relapse and disease progression in hematopoietic cancers and in a variety of solid tumors.^18,39^

Remarkably, the developed compounds have been shown to suppress elevated STAT5 levels in TKI-resistant CML cells and in therapy-resistant cancer cell lines from solid tumors per se. Overall, the data provide convincing evidence that the investigated drugs target both critical stem cell factors, increased STAT5 signaling and drug transporter activity via an independent dual mechanism of action.

To investigate the effects of telmisartan (**7a**) and the promising derivatives telmi-ester (**7b**) and telmi-amide (**7c**) on the accumulation of hematopoietic progenitor cells and cellular stem cells, colony-forming unit experiments were performed using treatment-free primary human CML patient samples. While **7a** was virtually inactive, compounds **7b** and **7c** significantly reduced colony formation when coadministered with imatinib, indicating their ability to selectively resensitize and eradicate leukemic CSCs.

Considering the results of the ADME and physicochemical assays, telmi-amide (**7c**) was selected for in vivo efficacy studies. Its permeability, metabolic stability, and hepatocytic stability all fell within desirable drug-like ranges and although the solubility was low, it was comparable to that of telmisartan.^29^ The ADME profile of **7c**, which overall is comparable to that of the FDA-approved drug telmisartan, underscores its potential to achieve therapeutic effects at clinically applicable concentrations. The observed IC_50_ values in vitro are comparable to the plasma concentrations of telmisartan achieved in clinical settings. For example, an oral dose of 120 mg telmisartan yields plasma levels of >3 μM in hypertensive patients.^40^ The in vivo studies using a human K562-R xenograft model in NOD/SCID mice confirm the efficacy of **7c** and imatinib both in monotherapy and in combination. Notably, the combination therapy significantly reduced tumor volume more than imatinib alone, demonstrating improved antitumor activity without toxic side effects. Oral availability offers further advantages, such as better treatment compliance and convenience.

In summary, the telmisartan derivatives exhibit specificity against therapy-resistant cancer cells by effectively and independently targeting two key CSC features: overactivation of STAT5 signaling and increased drug transporter activity. This dual mode of action distinguishes the compounds from therapeutically used drugs. The compounds increased the sensitivity of hematopoietic and solid tumor cells to chemotherapy at concentrations corresponding to the therapeutic range used in patients treated with telmisartan for hypertension as standard of care.^40^ In addition, the observed in vivo efficacy when administered orally offers therapeutic advantages for use in clinical practice. In view of these promising results, our compounds may have broad clinical applicability and offer new perspectives for overcoming therapy resistance through a cancer agnostic approach.

## 4 Conclusion

The promising concept of collateral sensitivity^41^ aims to simultaneously eradicate both sensitive and resistant cancer cells through combination therapy with different drugs. The applied chemotherapeutic agent is intended to eliminate native tumor cells. The addition of a noncytotoxic cell death modulator further enables the specific targeting of therapy-resistant CSCs, while sparing sensitive nonmalignant cells. This promising strategy could significantly improve the treatment of malignant diseases by overcoming the lack of efficacy of standard cancer treatment in refractory CSCs. Thus, the combination therapy with potent telmisartan derivatives could be the starting point for the pharmacological erosion of CSC pools and for ultimately cancer eradication in a broad variety of human malignancies.

## 5 Experimental Section

### 5.1 Chemistry

#### 5.1.1 General Materials and Methods

Anhydrous solvents and all chemicals were purchased from Alfa Aesar, Sigma-Aldrich, or TCI with a purity of over 95%. Other solvents were of high quality or freshly distilled prior to use. Anhydrous reactions were conducted under an inert argon atmosphere using oven-dried glassware. Column chromatography was carried out using an Isolera One 3.0 flash purification system (Biotage) or with classic standard procedures. In both cases, silica gel 60 (particle size 40–63 mm, 230–240 mesh) was used. Reactions were monitored by thin layer chromatography (TLC), using Polygram SIL G/UV_254_ polyester foils covered with a 0.2 mm layer of silica gel and a fluorescence indicator (Macherey-Nagel). TLC plates were visualized under UV-light at 254 and 366 nm. NMR spectra were recorded with a 400 MHz Avance 4 Neo (Bruker) spectrometer, using deuterated dimethyl sulfoxide (DMSO-*d*_6_), acetone (acetone-*d*_6_), or methanol (CD_3_OD) as solvents (Eurisotop). Chemical shifts are given in parts per million (ppm) and were referenced to tetramethylsilane (TMS) or the solvent peak. The coupling constants (*J*) are given in Hertz (Hz) and the signals are described as follows: s = singlet, br s = broad singlet, d = doublet, dd = doublet of doublets, t = triplet, m = multiplet. HRMS was performed using an Orbitrap Elite mass spectrometer (Thermo Fisher Scientific) in the positive mode. HPLC was carried out to ensure a purity of >95% for all target compounds. A Shimadzu Nexera-i LC-2040C-3D equipped with an autosampler SIL-20A HT, column oven CTO-10AS VP, degasser DGU-20A, detector SPD-M20A, and pumps LC-20AD, was employed. A C18 column (dimensions 250 × 4 mm, 5 μm particle size, Knauer) was used and the chromatograms were analyzed using the program LabSolutions 5.86 (Shimadzu).

#### 5.1.2 Synthesis of Target Compounds

##### 5.1.2.1 General Procedure for the *N*-Alkylation

The solution of **II** (1 equiv (eq)) in anhyd. DMF (≈1–2 mL/mmol) was cooled in an ice bath, treated with NaH (1.2 equiv) in small portions, and stirred until the formation of hydrogen was completed. The respective (bromomethyl)aryl (1.1 equiv) was added and the reaction mixture was stirred on ice for 30 min and then at room temperature for 12 h. Ice water was added to double the volume and the mixture was neutralized with 1 N HCl. Then, it was extracted with ethyl acetate (EtOAc) (3×), the organic layers were combined, washed with brine, dried over anhyd. Na_2_SO_4_, and filtered. The solvent was removed under reduced pressure and the crude product was purified by flash column chromatography using stepwise gradient elution (petrol ether (PE):EtOAc = 7:3 to 3:7).

Methyl 3-((2-propyl-1H-benzo[*d*]imidazole-1-yl)methyl)benzoate (**2b**): From **II** (0.5 g, 3.1 mmol), NaH (0.1 g, 3.8 mmol) and methyl 3-(bromomethyl)benzoate (0.8 g, 3.4 mmol) in anhyd. DMF (3 mL). Beige solid, yield: 93%. Purity: 97%. ^1^H NMR (400 MHz, acetone-*d*_6_, δ): 7.86 (d, ^3^*J* = 7.8 Hz, 1H, H6), 7.75–7.69 (m, 1H, H2), 7.63–7.55 (m, 1H, H4′), 7.53–7.40 (m, 2H, H5, H7′), 7.33 (d, ^3^*J* = 8.0 Hz, 1H, H4), 7.20–7.12 (m, 2H, H5′, H6′), 5.59 (s, 2H, NCH_2_), 3.81 (s, 3H, CO_2_*CH*_3_), 2.81 (t, ^3^*J* = 7.4 Hz, 2H, *CH*_2_CH_2_CH_3_), 1.80–1.68 (m, 2H, CH_2_*CH*_2_CH_3_), 0.93 (t, ^3^*J* = 7.3 Hz, 3H, CH_2_CH_2_*CH*_3_). ^13^C NMR (101 MHz, acetone-*d*_6_, δ): 166.9, 156.0, 144.2, 139.0, 136.5, 131.8, 131.7, 130.0, 129.3, 128.2, 122.7, 122.3, 119.8, 110.6, 52.4, 46.8, 21.5, 14.2. HRMS *m*/*z*: [M + H]^+^ calcd for C_19_H_20_N_2_O_2_, 309.1598; found, 309.1621.

Methyl 4-((2-propyl-1H-benzo[*d*]imidazole-1-yl)methyl)benzoate (**3b**): From **II** (0.5 g, 3.1 mmol), NaH (0.1 g, 3.8 mmol) and methyl 4-(bromomethyl)benzoate (0.8 g, 3.4 mmol) in anhyd. DMF (3 mL). Colorless solid, yield: 81%. Purity: 99%. ^1^H NMR (400 MHz, acetone-*d*_6_, δ): 7.92 (d, ^3^*J* = 8.4 Hz, 2H, H2, H6), 7.63–7.57 (m, 1H, H4′), 7.45–7.38 (m, 1H, H7′), 7.20 (d, ^3^*J* = 8.4 Hz, 2H, H5′, H6′), 7.18–7.13 (m, 2H, H3, H5), 5.59 (s, 2H, NCH_2_), 3.82 (s, 3H, CO_2_*CH*_3_), 2.79 (t, ^3^*J* = 7.5 Hz, 2H, *CH*_2_CH_2_CH_3_), 1.81–1.67 (m, 2H, CH_2_*CH*_2_CH_3_), 0.92 (t, ^3^*J* = 7.4 Hz, 3H, CH_2_CH_2_*CH*_3_). ^13^C NMR (101 MHz, acetone-*d*_6_, δ): 166.8, 156.0, 144.1, 143.5, 136.6, 130.7, 130.5, 127.4, 122.7, 122.3, 119.8, 110.5, 52.3, 46.9, 21.4, 14.2. HRMS *m*/*z*: [M + H]^+^ calcd for C_19_H_20_N_2_O_2_, 309.1598; found, 309.1621.

Methyl 4′-((2-propyl-1H-benzo[*d*]imidazole-1-yl)methyl)-[1,1′-biphenyl]-2-carboxylate (**4b**): From compound **II** (0.5 g, 3.1 mmol), NaH (0.2 g, 3.7 mmol) and methyl 4′-(bromomethyl)-[1,1′-biphenyl]-2-carboxylate (1.1 g, 3.4 mmol) in anhyd. DMF (6 mL). Colorless solid, yield: 58%. Purity: 99%. ^1^H NMR (400 MHz, DMSO-*d*_6_, δ): 7.72 (dd, ^3^*J* = 7.7 Hz, ^4^*J* = 1.4 Hz, 1H, H3), 7.64–7.55 (m, 2H, H5, H4″), 7.53–7.42 (m, 2H, H4, H7″), 7.38 (dd, ^3^*J* = 7.7 Hz, ^4^*J* = 1.3 Hz, 1H, H6), 7.24 (d, ^3^*J* = 8.3 Hz, 2H, H2′, H6′), 7.21–7.10 (m, 4H, H3′, H5′, H5′’, H6″), 5.54 (s, 2H, NCH_2_), 3.54 (s, 3H, CO_2_*CH*_3_), 2.84 (t, ^3^*J* = 7.5 Hz, 2H, *CH*_2_CH_2_CH_3_), 1.84–1.71 (m, 2H, CH_2_*CH*_2_CH_3_), 0.96 (t, ^3^*J* = 7.4 Hz, 3H, CH_2_CH_2_*CH*_3_). ^13^C NMR (101 MHz, DMSO-*d*_6_, δ): 168.4, 155.1, 142.4, 139.7, 136.2, 135.4, 131.5, 130.7, 130.5, 129.3, 128.5, 127.5, 126.4, 121.7, 121.3, 118.5, 110.1, 51.8, 45.7, 28.6, 20.3, 13.8. HRMS *m*/*z*: [M + H]^+^ calculated for C_25_H_24_N_2_O_2_, 385.1911; found, 385.1937.

Methyl 4′-((2-propyl-1H-benzo[*d*]imidazole-1-yl)methyl)-[1,1′-biphenyl]-4-carboxylate (**6b**): From compound **II** (0.2 g, 1.2 mmol), NaH (0.04 g, 1.50 mmol) and methyl 4′-(bromomethyl)-[1,1′-biphenyl]-4-carboxylate (0.4 g, 1.4 mmol) in anhyd. DMF (4 mL). Colorless solid, yield: 59%. Purity: 99%. ^1^H NMR (400 MHz, acetone-*d*_6_, δ): 8.07 (d, ^3^*J* = 8.7 Hz, 2H, H2, H6), 7.78 (d, ^3^*J* = 8.8 Hz, 2H, H3, H5), 7.71 (d, ^3^*J* = 8.4 Hz, 2H, H2′, H6′), 7.64–7.59 (m, 1H, H4′′), 7.43–7.38 (m, 1H, H7′′), 7.25 (d, ^3^*J* = 8.7 Hz, 2H, H3′, H5′), 7.21–7.13 (m, 2H, H5′′, H6′′), 5.59 (s, 2H, NCH_2_), 3.89 (s, 3H, CO_2_*CH*_3_), 2.90 (t, ^3^*J* = 7.6 Hz, 2H, *CH*_2_CH_2_CH_3_), 1.94–1.82 (m, 2H, CH_2_*CH*_2_CH_3_), 1.01 (t, ^3^*J* = 7.4 Hz, 3H, CH_2_CH_2_*CH*_3_). ^13^C NMR (101 MHz, acetone-*d*_6_, δ): 167.0, 156.0, 145.6, 144.2, 139.8, 138.4, 136.7, 130.8, 130.1, 128.4, 128.0, 127.8, 122.6, 122.3, 119.8, 110.7, 52.4, 46.9, 21.5, 14.2. HRMS *m*/*z*: [M + H]^+^ calcd for C_25_H_24_N_2_O_2_, 385.1911; found, 385.1900.

##### 5.1.2.2 General Procedure for the Hydrolysis

Finely ground KOH (5–10 equiv) was dissolved in EG (≈ 0.5–1.5 mL/mmol starting material) and the solution was added to the respective carbonitrile or methyl ester (1 equiv). A few drops of water were added and the reaction mixture was heated at 160 °C for 12–72 h. After cooling to room temperature, water was added to double the volume and 6 N HCl was used to obtain a pH of 2. Unless a precipitate has formed, the solution was extracted with dichloromethane (DCM) or EtOAc (3×), and the organic layers were combined, washed with brine, dried over anhyd. Na_2_SO_4_, and filtered. The solvent was removed under reduced pressure. In case of precipitation, the product was sucked off, washed with cold water, and dried. The resulting crude product was purified using column chromatography (DCM/MeOH = 95:5) and recrystallized from MeOH.

2-((2-Propyl-1H-benzo[*d*]imidazole-1-yl)methyl)benzoic acid (**1a**): From **III** (1.1 g, 4.0 mmol) and KOH (1.1 g, 20.0 mmol) in EG (4 mL). Colorless solid, yield: 71%. Purity: 99%. ^1^H NMR (400 MHz, DMSO-*d*_6_, δ): 8.05–7.96 (m, 1H, H4′), 7.62 (d, ^3^*J* = 7.2 Hz, 1H, H6), 7.42–7.34 (m, 2H, H5′, H6′), 7.30 (d, ^3^*J* = 7.4 Hz, 1H, H3), 7.21–7.06 (m, 2H, H4, H5), 6.30–6.23 (m, 1H, H7′), 5.84 (s, 2H, NCH_2_), 2.72 (t, ^3^*J* = 7.5 Hz, 2H, *CH*_2_CH_2_CH_3_), 1.79–1.67 (m, 2H, CH_2_*CH*_2_CH_3_), 0.91 (t, ^3^*J* = 7.4 Hz, 3H, CH_2_CH_2_*CH*_3_). ^13^C NMR (101 MHz, DMSO-*d*_6_, δ): 168.2, 155.3, 142.4, 138.3, 135.4, 132.5, 131.0, 129.1, 127.4, 125.5, 121.8, 121.4, 118.5, 110.0, 44.7, 28.4, 20.2, 13.7. HRMS *m*/*z*: [M + H]^+^ calcd for C_18_H_18_N_2_O_2_, 295.1441; found, 295.1449.

3-((2-Propyl-1H-benzo[*d*]imidazole-1-yl)methyl)benzoic acid (**2a**): From **2b** (0.6 g, 2.1 mmol) and KOH (0.6 g, 10.3 mmol) in EG (2 mL). Off-white solid, yield: 71%. Purity: 99%. ^1^H NMR (400 MHz, DMSO-*d*_6_, δ): 7.84 (d, ^3^*J* = 7.8 Hz, 1H, H6), 7.69–7.65 (m, 1H, H2), 7.63–7.57 (m, 1H, H4′), 7.50–7.41 (m, 2H, H5, H7′), 7.33 (d, ^3^*J* = 7.4 Hz, 1H, H4), 7.19–7.13 (m, 2H, H5′, H6′), 5.58 (s, 2H, NCH_2_), 2.80 (t, ^3^*J* = 7.5 Hz, 2H, *CH*_2_CH_2_CH_3_), 1.80–1.69 (m, 2H, CH_2_*CH*_2_CH_3_), 0.93 (t, ^3^*J* = 7.4 Hz, 3H, CH_2_CH_2_*CH*_3_). ^13^C NMR (101 MHz, DMSO-*d*_6_, δ): 167.0, 155.0, 142.4, 137.8, 135.3, 131.3, 130.8, 129.1, 128.4, 127.1, 121.8, 121.4, 118.5, 110.1, 45.6, 28.5, 20.3, 13.7. HRMS *m*/*z*: [M + H]^+^ calcd for C_18_H_18_N_2_O_2_, 295.1441; found, 295.1464.

4-((2-Propyl-1H-benzo[*d*]imidazole-1-yl)methyl)benzoic acid (**3a**): From **3b** (0.7 g, 2.2 mmol) and KOH (0.6 g, 11.2 mmol) in EG (2 mL). Colorless solid, yield: 68%. Purity: 99%. ^1^H NMR (400 MHz, DMSO-*d*_6_, δ): 7.89 (d, ^3^*J* = 8.3 Hz, 2H, H2, H6), 7.64–7.55 (m, 1H, H4′), 7.46–7.39 (m, 1H, H7′), 7.21–7.12 (m, 4H, H3, H5, H5′, H6′), 5.59 (s, 2H, NCH_2_), 2.80 (t, ^3^*J* = 7.5 Hz, 2H, *CH*_2_CH_2_CH_3_), 1.80–1.68 (m, 2H, CH_2_*CH*_2_CH_3_), 0.93 (t, ^3^*J* = 7.4 Hz, 3H, CH_2_CH_2_*CH*_3_). ^13^C NMR (101 MHz, DMSO-*d*_6_, δ): 167.0, 155.1, 142.4, 142.2, 135.3, 130.0, 129.8, 126.5, 121.8, 121.4, 118.5, 110.0, 45.7, 28.5, 20.3, 13.8. HRMS *m*/*z*: [M + H]^+^ calcd for C_18_H_18_N_2_O_2_, 295.1441; found, 295.1469.

4′-((2-Propyl-1H-benzo[*d*]imidazole-1-yl)methyl)-[1,1′-biphenyl]-2-carboxylic acid (**4a**): From **4b** (0.5 g, 1.3 mmol) and KOH (0.2 g, 2.6 mmol) in EG (2 mL). Colorless solid, yield: 75%. Purity: 99%. ^1^H NMR (400 MHz, DMSO-*d*_6_, δ): 12.80 (br s, 1H, COOH), 7.67 (dd, ^3^*J* = 7.6 Hz, ^4^*J* = 1.5 Hz, 1H, H3), 7.62–7.56 (m, 1H, H5), 7.54–7.47 (m, 2H, H4″, H7″), 7.44–7.38 (m, 1H, H4), 7.34–7.27 (m, 3H, H6, H6′, H2′), 7.20–7.14 (m, 2H, H6″, H5′’), 7.11 (d, ^3^*J* = 8.2 Hz, 2H, H3′, H5′), 5.53 (s, 2H, NCH_2_), 2.84 (t, ^3^*J* = 7.5 Hz, 2H, *CH*_2_CH_2_CH_3_), 1.87–1.71 (m, 2H, CH_2_*CH*_2_CH_3_), 0.96 (t, ^3^*J* = 7.4 Hz, 3H, CH_2_CH_2_*CH*_3_). ^13^C NMR (101 MHz, DMSO-*d*_6_, δ): 169.9, 155.4, 140.9, 140.8, 135.8, 134.9, 132.7, 131.4, 130.9, 129.6, 129.2, 127.9, 126.9, 123.3, 123.2, 117.8, 111.5, 46.6, 28.5, 20.7, 14.2. HRMS *m*/*z*: [M + H]^+^ calcd for C_24_H_22_N_2_O_2_, 371.1754; found, 371.1773.

4′-((2-Propyl-1H-benzo[*d*]imidazole-1-yl)methyl)-[1,1′-biphenyl]-3-carboxylic acid (**5a**): From **VIII** (0.5 g, 1.3 mmol) and KOH (0.4 g, 6.7 mmol) in EG (3 mL). Colorless solid, yield: 82%. Purity: 99%. ^1^H NMR (400 MHz, DMSO-*d*_6_, δ): 8.17–8.12 (m, 1H, H2), 7.92 (d, ^3^*J* = 7.7 Hz, 1H, H4), 7.87 (d, ^3^*J* = 8.3 Hz, 1H, H6), 7.67 (d, ^3^*J* = 8.3 Hz, 2H, H2′, H6′), 7.63–7.55 (m, 2H, H5, H4″), 7.50–7.46 (m, 1H, H7″), 7.23–7.13 (m, 4H, H3′, H5′, H5′’, H6″), 5.56 (s, 2H, NCH_2_), 2.85 (t, ^3^*J* = 7.5 Hz, 2H, *CH*_2_CH_2_CH_3_), 1.84–1.73 (m, 2H, CH_2_*CH*_2_CH_3_), 0.96 (t, ^3^*J* = 7.4 Hz, 3H, CH_2_CH_2_*CH*_3_). ^13^C NMR (101 MHz, DMSO-*d*_6_, δ): 167.3, 155.1, 142.4, 139.9, 138.5, 136.9, 135.3, 131.7, 131.0, 129.3, 128.3, 127.2, 127.2, 121.7, 121.4, 118.5, 110.2, 45.7, 28.5, 20.4, 13.8. HRMS *m*/*z*: [M + H]^+^ calcd for C_24_H_22_N_2_O_2_, 371.1754; found, 371.1746.

4′-((2-Propyl-1H-benzo[*d*]imidazole-1-yl)methyl)-[1,1′-biphenyl]-4-carboxylic acid (**6a**): From **6b** (0.2 g, 0.4 mmol) and KOH (0.2 g, 3.9 mmol) in EG (2 mL). Colorless solid, yield: 83%. Purity: 99%. ^1^H NMR (400 MHz, DMSO-*d*_6_, δ): 12.96 (br s, 1H, COOH), 8.00 (d, ^3^*J* = 8.4 Hz, 2H, H2, H6), 7.77 (d, ^3^*J* = 8.3 Hz, 2H, H3, H5), 7.74–7.66 (m, 3H, H2′, H6′, H4″), 7.62–7.56 (m, 1H, H7″), 7.32–7.23 (m, 4H, H3′, H5′, H5′’, H6″), 5.65 (s, 2H, NCH_2_), 2.97 (t, ^3^*J* = 7.6 Hz, 2H, *CH*_2_CH_2_CH_3_), 1.88–1.74 (m, 2H, CH_2_*CH*_2_CH_3_), 0.97 (t, ^3^*J* = 7.4 Hz, 3H, CH_2_CH_2_*CH*_3_). ^13^C NMR (101 MHz, DMSO-*d*_6_, δ): 167.1, 154.9, 143.6, 138.3, 136.5, 134.3, 129.9, 129.7, 127.4, 127.3, 126.7, 122.9, 122.8, 117.2, 111.0, 62.8, 46.1, 28.0, 20.2, 13.7. HRMS *m*/*z*: [M + H]^+^ calcd for C_24_H_22_N_2_O_2_, 371.1754; found, 371.1745.

##### 5.1.2.3 General Procedure for the Esterification

The respective carboxylic acid (1 equiv) was dissolved in anhyd. MeOH (25 mL/mmol) under an argon atmosphere and it was cooled in an ice bath. Thionyl chloride (6 mL/mmol) was slowly added and the solution was stirred at room temperature for 12 h. The solvent was then removed under reduced pressure and ice water was added to the residue. NaOH (1 N) was used to attain a pH of 8 and it was extracted with DCM (3×). The organic layers were combined, washed with brine, dried over anhyd. Na_2_SO_4_, and filtered. DCM was removed under reduced pressure. The resulting product was purified by flash column chromatography using a stepwise gradient elution (PE/EtOAc = 9:1 to 3:7).

Methyl 2-((2-propyl-1H-benzo[*d*]imidazole-1-yl)methyl)benzoate (**1b**): From **1a** (0.1 g, 0.3 mmol) and thionyl chloride (2 mL) in anhyd. MeOH (8 mL). Beige solid, yield: 88%. Purity: 99%. ^1^H NMR (400 MHz, acetone-*d*_6_, δ): 8.11–8.05 (m, 1H, H4′), 7.64 (d, ^3^*J* = 7.5 Hz, 1H, H6), 7.44–7.38 (m, 2H, H5′, H6′), 7.25 (d, ^3^*J* = 7.4 Hz, 1H, H3), 7.22–7.09 (m, 2H, H4, H5), 6.41–6.33 (m, 1H, H7′), 5.88 (s, 2H, NCH_2_), 3.96 (s, 3H, CO_2_*CH*_3_), 2.77 (t, ^3^*J* = 7.6 Hz, 2H, *CH*_2_CH_2_CH_3_), 1.91–1.77 (m, 2H, CH_2_*CH*_2_CH_3_), 0.97 (t, ^3^*J* = 7.4 Hz, 3H, CH_2_CH_2_*CH*_3_). ^13^C NMR (101 MHz, acetone-*d*_6_, δ): 167.8, 156.2, 144.2, 139.8, 136.7, 133.7, 131.9, 129.2, 128.2, 126.7, 122.7, 122.3, 119.8, 110.5, 52.5, 45.8, 21.4, 14.2. HRMS *m*/*z*: [M + H]^+^ calcd for C_19_H_20_N_2_O_2_, 309.1598; found, 309.1617.

Methyl 4′-((2-propyl-1H-benzo[*d*]imidazole-1-yl)methyl)-[1,1′-biphenyl]-3-carboxylate (**5b**): From **5a** (0.1 g, 0.2 mmol) with thionyl chloride (2 mL) in anhyd. MeOH (8 mL). Colorless solid, yield: 82%. Purity: 99%. ^1^H NMR (400 MHz, acetone-*d*_6_, δ): 8.25–8.21 (m, 1H, H2), 7.98 (d, ^3^*J* = 7.8 Hz, 1H, H4), 7.88 (d, ^3^*J* = 7.8 Hz, 1H, H6), 7.67 (d, ^3^*J* = 8.3 Hz, 2H, H2′, H6′), 7.65–7.55 (m, 2H, H5, H4″), 7.44–7.38 (m, 1H, H7″), 7.25 (d, ^3^*J* = 8.4 Hz, 2H, H3′, H5′), 7.22–7.14 (m, 2H, H5′’, H6″), 5.59 (s, 2H, NCH_2_), 3.90 (s, 3H, CO_2_*CH*_3_), 2.90 (t, ^3^*J* = 7.4 Hz, 2H, *CH*_2_CH_2_CH_3_), 1.94–1.82 (m, 2H, CH_2_*CH*_2_CH_3_), 1.01 (t, ^3^*J* = 7.4 Hz, 3H, CH_2_CH_2_*CH*_3_). ^13^C NMR (101 MHz, DMSO-*d*_6_, δ): 167.1, 156.0, 143.9, 141.6, 140.1, 137.9, 136.6, 132.2, 131.9, 130.1, 129.1, 128.4, 128.3, 128.1, 122.7, 122.4, 119.7, 110.7, 52.5, 46.9, 21.5, 14.2. HRMS *m*/*z*: [M + H]^+^ calcd for C_25_H_24_N_2_O_2_, 385.1911; found, 385.1900.

Methyl 4′-((1,7′-dimethyl-2′-propyl-1H,3′H-[2,5′-bibenzo[*d*]imidazole]-3′-yl)methyl)-[1,1′-biphenyl]-2-carboxylate (**7b**): From **7a** (0.1 g, 0.1 mmol) with thionyl chloride (2 mL) in anhyd. MeOH (8 mL). Colorless solid, yield: 78%. Purity: 99%. ^1^H NMR (400 MHz, acetone-*d*_6_, δ): 7.75 (dd, ^3^*J* = 7.7 Hz, ^4^*J* = 1.0 Hz, 1H, Ar–H), 7.68–7.62 (m, 2H, Ar–H), 7.59–7.54 (m, 1H, Ar–H), 7.54–7.51 (m, 1H, Ar–H), 7.49–7.42 (m, 2H, Ar–H), 7.38 (dd, ^3^*J* = 7.7 Hz, ^4^*J* = 0.9 Hz, 1H, Ar–H), 7.31–7.19 (m, 6H, Ar–H), 5.64 (s, 2H, NCH_2_), 3.86 (s, 3H, CO_2_*CH*_3_), 3.51 (s, 3H, NCH_3_), 2.98 (t, ^3^*J* = 7.6 Hz, 2H, *CH*_2_CH_2_CH_3_), 2.69 (s, 3H, *CH*_3_), 2.00–1.86 (m, 2H, CH_2_*CH*_2_CH_3_), 1.06 (t, ^3^*J* = 7.4 Hz, 3H, CH_2_CH_2_*CH*_3_). ^13^C NMR (101 MHz, acetone-*d*_6_, δ): 169.3, 157.1, 155.4, 144.2, 142.4, 141.6, 138.0, 137.1, 136.1, 132.2, 132.1, 131.4, 130.4, 129.7, 128.2, 127.3, 125.0, 124.3, 122.8, 122.5, 119.9, 110.7, 110.0, 52.1, 47.4, 32.2, 21.8, 16.7, 14.3. HRMS *m*/*z*: [M + H]^+^ calcd for C_34_H_32_N_4_O_2_, 529.2598; found, 529.2606.

##### 5.1.2.4 General Procedure for the Amidation

The respective carboxylic acid (1 equiv) was dissolved in anhyd. DMF (≈3–4 mL/mmol) and cooled to 0 °C in an ice bath. PyBOP (2 equiv) in anhyd. DMF (≈2–3 mL/mmol) was added and the reaction mixture was stirred for 5 min. After adding DIPEA (4 equiv) and a solution of NH_4_Cl (2 equiv) in anhyd. DMF (≈2–4 mL/mmol), it was stirred for another 30 min at 0 °C. Then, the ice bath was removed and stirring was continued for 12–16 h at room temperature. The mixture was diluted with water to double the volume and it was alkalized to a pH of 9 with 1 N NaOH. The solution was extracted with EtOAc (3×) to remove the formed 1-hydroxybenzotriazole (HOBt) and unreacted carboxylic acid. The organic layers were combined, washed with brine, dried over anhyd. Na_2_SO_4_, and filtered. EtOAc was removed under reduced pressure. The resulting product was purified by column chromatography (DCM/MeOH = 10:0 to 9:1) and flash column chromatography (PE/EtOAc = 7:3 to 0:10) with stepwise gradient elution.

2-((2-Propyl-1H-benzo[*d*]imidazole-1-yl)methyl)benzamide (**1c**): From **1a** (0.2 g, 0.7 mmol) in anhyd. DMF (2 mL), PyBOP (0.7 g, 1.4 mmol) dissolved in anhyd. DMF (3 mL), DIPEA (0.5 mL), and NH_4_Cl (0.1 g, 1.4 mmol) suspended in anhyd. DMF (3 mL). Colorless solid, yield: 91%. Purity: 99%. ^1^H NMR (400 MHz, DMSO-*d*_6_, δ): 8.06 (br s, 1H, CONH), 7.66–7.55 (m, 3H, CONH, H6, H4′), 7.36–7.23 (m, 3H, H3, H5′, H6′), 7.20–7.08 (m, 2H, H4, H5), 6.41 (dd, ^3^*J* = 7.7 Hz, ^4^*J* = 1.3 Hz, 1H, H7′), 5.65 (s, 2H, NCH_2_), 2.75 (t, ^3^*J* = 7.5 Hz, 2H, *CH*_2_CH_2_CH_3_), 1.82–1.68 (m, 2H, CH_2_*CH*_2_CH_3_), 0.93 (t, ^3^*J* = 7.4 Hz, 3H, CH_2_CH_2_*CH*_3_). ^13^C NMR (101 MHz, DMSO-*d*_6_, δ): 170.2, 155.2, 142.4, 135.4, 135.2, 135.0, 130.2, 127.8, 127.2, 125.7, 121.7, 121.3, 118.5, 109.9, 43.8, 28.4, 20.3, 13.8. HRMS *m*/*z*: [M + H]^+^ calcd for C_18_H_19_N_3_O, 294.1601; found, 294.1616.

3-((2-Propyl-1H-benzo[*d*]imidazole-1-yl)methyl)benzamide (**2c**): From **2a** (0.1 g, 0.3 mmol) in anhyd. DMF (1 mL), PyBOP (0.4 g, 0.7 mmol) dissolved in anhyd. DMF (1.5 mL), DIPEA (0.2 mL), and NH_4_Cl (0.04 g, 0.7 mmol) suspended in anhyd. DMF (1.5 mL). Colorless solid, yield: 87%. Purity: 99%. ^1^H NMR (400 MHz, DMSO-*d*_6_, δ): 7.98 (br s, 1H, CONH), 7.76 (d, ^3^*J* = 7.9 Hz, 1H, H6), 7.70–7.65 (m, 1H, H2), 7.62–7.55 (m, 1H, H4′), 7.46–7.32 (m, 3H, CONH, H5, H7′), 7.21–7.10 (m, 3H, H4, H5′, H6′), 5.53 (s, 2H, NCH_2_), 2.81 (t, ^3^*J* = 7.5 Hz, 2H, *CH*_2_CH_2_CH_3_), 1.81–1.70 (m, 2H, CH_2_*CH*_2_CH_3_), 0.94 (t, ^3^*J* = 7.4 Hz, 3H, CH_2_CH_2_*CH*_3_). ^13^C NMR (101 MHz, DMSO-*d*_6_, δ): 167.5, 155.0, 142.4, 137.4, 135.3, 134.7, 129.2, 128.6, 126.3, 125.9, 121.7, 121.3, 118.5, 110.1, 45.9, 28.5, 20.3, 13.8. HRMS *m*/*z*: [M + H]^+^ calcd for C_18_H_19_N_3_O, 294.1601; found, 294.1621.

4-((2-Propyl-1H-benzo[*d*]imidazole-1-yl)methyl)benzamide (**3c**): From **3a** (0.1 g, 0.3 mmol) in anhyd. DMF (1 mL), PyBOP (0.4 g, 0.7 mmol) dissolved in anhyd. DMF (1.5 mL), DIPEA (0.2 mL), and NH_4_Cl (0.04 g, 0.7 mmol) suspended in anhyd. DMF (1.5 mL). Colorless solid, yield: 88%. Purity: 99%. ^1^H NMR (400 MHz, DMSO-*d*_6_, δ): 7.91 (br s, 1H, CONH), 7.80 (d, ^3^*J* = 8.4 Hz, 2H, H2, H6), 7.63–7.55 (m, 1H, H4′), 7.45–7.38 (m, 1H, H7′), 7.33 (br s, 1H, CONH), 7.19–7.09 (m, 4H, H3, H5, H5′, H6′), 5.54 (s, 2H, NCH_2_), 2.80 (t, ^3^*J* = 7.5 Hz, 2H, *CH*_2_CH_2_CH_3_), 1.81–1.69 (m, 2H, CH_2_*CH*_2_CH_3_), 0.94 (t, ^3^*J* = 7.4 Hz, 3H, CH_2_CH_2_*CH*_3_). ^13^C NMR (101 MHz, DMSO-*d*_6_, δ): 167.5, 155.0, 142.4, 140.4, 135.3, 133.5, 127.9, 126.2, 121.7, 121.4, 118.5, 110.1, 45.7, 28.5, 20.3, 13.8. HRMS *m*/*z*: [M + H]^+^ calcd for C_18_H_19_N_3_O, 294.1601; found, 294.1643.

4′-((2-Propyl-1H-benzo[*d*]imidazole-1-yl)methyl)-[1,1′-biphenyl]-2-carboxamide (**4c**): From **4a** (0.1 g, 0.3 mmol) in anhyd. DMF (1 mL), PyBOP (0.3 g, 0.5 mmol) dissolved in anhyd. DMF (1 mL), DIPEA (0.2 mL), and NH_4_Cl (0.03 g, 0.5 mmol) suspended in anhyd. DMF (3 mL). Colorless solid, yield: 59%. Purity: 99%. ^1^H NMR (400 MHz, CD_3_OD, δ): 7.66–7.60 (m, 1H, H4″), 7.54–7.45 (m, 2H, H3, H5), 7.44–7.34 (m, 5H, H4, H6, H2′, H6′, H7″), 7.28–7.20 (m, 2H, H5″, H6″), 7.12 (d, ^3^*J* = 8.3 Hz, 2H, H3′, H5′), 5.53 (s, 2H, NCH_2_), 2.92 (t, ^3^*J* = 7.8 Hz, 2H, *CH*_2_CH_2_CH_3_), 1.85–1.74 (m, 2H, CH_2_*CH*_2_CH_3_), 0.89 (t, ^3^*J* = 7.1 Hz, 3H, CH_2_CH_2_*CH*_3_). ^13^C NMR (101 MHz, CD_3_OD, δ): 175.6, 157.2, 143.0, 141.5, 140.6, 137.3, 137.2, 136.5, 131.4, 131.1, 130.3, 128.8, 128.4, 127.4, 123.8, 123.4, 119.2, 111.3, 47.5, 32.6, 28.5, 28.2, 23.4, 14.3. HRMS *m*/*z*: [M + H]^+^ calcd for C_24_H_23_N_3_O, 370.1914; found, 370.1954.

4′-((2-Propyl-1H-benzo[*d*]imidazole-1-yl)methyl)-[1,1′-biphenyl]-3-carboxamide (**5c**): From **5a** (0.1 g, 0.3 mmol) in anhyd. DMF (1 mL), PyBOP (0.3 g, 0.5 mmol) dissolved in anhyd. DMF (1 mL), DIPEA (0.2 mL), and NH_4_Cl (0.03 g, 0.54 mmol) suspended in anhyd. DMF (3 mL). Colorless solid, yield: 69%. Purity: 99%. ^1^H NMR (400 MHz, DMSO-*d*_6_): δ 8.14–8.09 (m, 1H, H2), 8.06 (br s, 1H, CONH), 7.84 (d, ^3^*J* = 7.8 Hz, 1H, H4), 7.77 (d, ^3^*J* = 8.1 Hz, 1H, H6), 7.68 (d, ^3^*J* = 8.3 Hz, 2H, H2′, H6′), 7.63–7.57 (m, 1H, H4″), 7.55–7.44 (m, 2H, H5, H7″), 7.41 (br s, 1H, CONH), 7.23–7.13 (m, 4H, H3′, H5′, H5′’, H6″), 5.55 (s, 2H, NCH_2_), 2.85 (t, ^3^*J* = 7.5 Hz, 2H, *CH*_2_CH_2_CH_3_), 1.85–1.73 (m, 2H, CH_2_*CH*_2_CH_3_), 0.97 (t, ^3^*J* = 7.4 Hz, 3H, CH_2_CH_2_*CH*_3_). ^13^C NMR (101 MHz, DMSO-*d*_6_, δ): 167.7, 155.1, 142.4, 139.6, 138.8, 136.7, 135.3, 134.9, 129.3, 128.9, 127.2, 127.1, 126.6, 125.6, 121.7, 121.4, 118.5, 110.2, 45.7, 28.5, 20.4, 13.8. HRMS *m*/*z*: [M + H]^+^ calcd for C_24_H_23_N_3_O, 370.1914; found, 370.1904.

4′-((2-Propyl-1H-benzo[*d*]imidazole-1-yl)methyl)-[1,1′-biphenyl]-4-carboxamide (**6c**): From **6a** (0.1 g, 0.3 mmol) in anhyd. DMF (1 mL), PyBOP (0.3 g, 0.5 mmol) dissolved in anhyd. DMF (1 mL), DIPEA (0.2 mL), and NH_4_Cl (0.03 g, 0.54 mmol) suspended in anhyd. DMF (3 mL). Colorless solid, yield: 73%. Purity: 99%. ^1^H NMR (400 MHz, acetone-*d*_6_, δ): 8.01 (d, ^3^*J* = 8.7 Hz, 2H, H2, H6), 7.72 (d, ^3^*J* = 8.6 Hz, 2H, H3, H5), 7.69 (d, ^3^*J* = 8.4 Hz, 2H, H2′, H6′), 7.64–7.59 (m, 1H, H4″), 7.54–7.38 (m, 2H, CONH, H7″), 7.24 (d, ^3^*J* = 8.7 Hz, 2H, H3′, H5′), 7.21–7.13 (m, 2H, H5′’, H6″), 6.62 (br s, 1H, CONH), 5.58 (s, 2H, NCH_2_), 2.89 (t, ^3^*J* = 7.4 Hz, 2H, *CH*_2_CH_2_CH_3_), 1.92–1.81 (m, 2H, CH_2_*CH*_2_CH_3_), 1.01 (t, ^3^*J* = 7.4 Hz, 3H, CH_2_CH_2_*CH*_3_). ^13^C NMR (101 MHz, DMSO-*d*_6_, δ): 168.5, 156.0, 144.1, 144.0, 140.2, 138.0, 136.6, 134.3, 129.1, 128.3, 128.0, 127.5, 122.6, 122.3, 119.8, 110.7, 46.9, 21.5, 14.2. HRMS *m*/*z*: [M + H]^+^ calcd for C_24_H_23_N_3_O, 370.1914; found, 370.1905.

4′-((1,7′-Dimethyl-2′-propyl-1H,3′H-[2,5′-bibenzo[*d*]imidazole]-3′-yl)methyl)-[1,1′-biphenyl]-2-carboxamide (**7c**): From **7a** (0.1 g, 0.2 mmol) in anhyd. DMF (1 mL), PyBOP (0.1 g, 0.3 mmol) dissolved in anhyd. DMF (1 mL), DIPEA (0.2 mL), and NH_4_Cl (0.02 g, 0.28 mmol) suspended in anhyd. DMF (1 mL). Colorless solid, yield: 17%. Purity: 99%. ^1^H NMR (400 MHz, DMSO-*d*_6_, δ): 7.77–7.73 (m, 1H, Ar–H), 7.66–7.56 (m, 3H, Ar–H, CONH), 7.50–7.34 (m, 6H, Ar–H, CONH), 7.32 (dd, ^3^*J* = 7.6 Hz, ^4^*J* = 1.3 Hz, 1H, Ar–H), 7.30–7.19 (m, 3H, Ar–H), 7.17 (d, ^3^*J* = 8.3 Hz, 2H, Ar–H), 5.61 (s, 2H, NCH_2_), 3.82 (s, 3H, NCH_3_), 2.93 (t, ^3^*J* = 7.6 Hz, 2H, *CH*_2_CH_2_CH_3_), 2.63 (s, 3H, *CH*_3_), 1.89–1.77 (m, 2H, CH_2_*CH*_2_CH_3_), 1.01 (t, ^3^*J* = 7.4 Hz, 3H, CH_2_CH_2_*CH*_3_). ^13^C NMR (101 MHz, DMSO-*d*_6_, δ): 171.0, 156.2, 154.0, 142.7, 142.5, 139.7, 138.3, 137.4, 136.7, 135.95, 134.7, 129.8, 129.2, 128.7, 128.2, 127.5, 127.1, 126.4, 123.3, 123.2, 122.0, 121.8, 118.7, 110.4, 109.3, 46.0, 31.8, 28.7, 20.7, 16.5, 13.9. HRMS *m*/*z*: [M + H]^+^ calcd for C_33_H_31_N_5_O, 514.2601; found, 514.2653.

### 5.2 Biology

#### 5.2.1 General Cell Culture Methods

The African green monkey kidney fibroblast-like cell line COS-7 (ATCC, Wesel, Germany; RRID/CVCL_0224) was cultured as monolayer culture in Dulbecco’s modified Eagle’s medium (DMEM) with 4.5 g/L glucose and 584 mg/L l-glutamine (GE Healthcare), supplemented with fetal calf serum (FCS 10%, Sigma-Aldrich), without phenol red or sodium pyruvate. The drug-resistant CML cell line, derived from K562 (RRID/CVCL_0004) cells, was a kind gift from Ernesto Yague and originally described as a doxorubicin-resistant subclone termed KD225 (termed K562-R in the present study).^42^ The breast cancer cell line MCF7 (RRID/CVCL_0031) and the lung cancer cell line A549 (RRID/CVCL_0023) were purchased from ATCC (Wesel, Germany). The CSC-defining side population (SP) of the ovarian cancer cell lines IGROV-1 (RRID/CVCL_1304) and A2780 V (“Vienna” variant of A2780; RRID/CVCL_0134) was enriched by FACS to generate the drug resistant subclones IGROV-1 SP and A2780 V SP.^26^ PC3 (RRID/CVCL_0035) and DU145 (RRID/CVCL_0105) cells were cultivated under increasing concentrations of docetaxel and subsequently cultured under the presence of 12.5  nM docetaxel to generate and maintain the docetaxel-resistant subclones PC3-DR and DU145-DR.^43^ K562, K562-R, IGROV-1 SP, PC3-R and DU145-DR cells were cultivated in Roswell Park Memorial Institute (RPMI) 1640 medium (Lonza) supplemented with 10% FCS, 2 mM l-glutamine, 100 U/mL penicillin, 100 μg/mL streptomycin (Sigma-Aldrich). A2780 V SP, MCF7, as well as A549 cells were cultured in DMEM supplemented with 10% FCS, 2 mM l-glutamine, 100 U/mL penicillin, 100 μg/mL streptomycin (Sigma-Aldrich). All cell lines were incubated in a humidified atmosphere (5% CO_2_/95% air) at 37 °C and were passaged twice per week.

#### 5.2.2 Determination of Metabolic Activity

COS-7 (2 × 10^3^ cells per well), IGROV-1 SP (2.5 × 10^3^ cells per well), A2780 V SP (1.5 × 10^3^ cells per well), PC3-DR (4 × 10^3^ cells per well), and DU145-DR (3 × 10^3^ cells per well) cells were seeded in 96-well plates in triplicates and after 24 h, the compounds were added at the respective concentrations. K562-R and K562 cells (2 × 10^4^ cells per well), were treated with the compounds 2 h after seeding. The cells were incubated at 37 °C in a humidified atmosphere (5% CO_2_/95% air) for 72 h. A modified MTT colorimetric assay (EZ4U kit, Biomedica) was used to determine the metabolic activity of the tested cells, according to the manufacturer’s instructions. Absorbance was measured and the optical density of the respective FCS-containing medium and of the substrate was subtracted to exclude nonspecific staining. Metabolic activity in the absence of the compounds (CTR, DMSO) was set to 100% as reference. The results of the compounds in all assays are represented by the mean values + SEM of ≥3 independent experiments with three replicates each.

#### 5.2.3 Analyses of Protein Expression

Protein extracts were prepared as previously described.^44^ Protein expression was determined using the capillary-based Jess Simple Western detection system (ProteinSimple, BioTechne) according to the manufacturer’s instructions at default settings. Antibodies specific to STAT5 (R&D Systems #AF2168; RRID/AB_355174), ABCB1 (Cell Signaling #12683; RRID/AB_2715689), ABCC1 (Cell Signaling #72202; RRID/AB_2799816), ABCG2 (Cell Signaling #42078; RRID/AB_2799211), or GAPDH (Cell Signaling #2118; RRID/AB_561053) each combined with 0.2 mg/mL protein lysate, and the pSTAT5 antibody (Millipore #05–886R; RRID/AB_1977514) in combination with 2 mg/mL protein lysate were used. Horseradish peroxidase (HRP)-conjugated secondary antirabbit antibody, which was included in the Jess Detection Module kit, was used. Data analysis was conducted using Compass for Simple Western 4.0 software (ProteinSimple, BioTechne).

#### 5.2.4 Flow Cytometry for SP Analyses and CD44 Protein Expression

SP analyses were performed as previously described^45^ on a LSRFortessa flow cytometer (BD Biosciences, Vienna, Austria). Briefly, 1 × 10^6^ cells/mL were stained with 10 μM Vybrant DyeCycleTM Violet (DCV; Molecular Probes, Eugene, OR) in the presence or absence of the investigated compounds for 90 min at 37 °C. The proportion of CD44 positive cells was determined by staining with a FITC-labeled antibody (clone DB105, Miltenyi, Bergisch Gladbach, BRD) at pretitrated concentrations for 30 min at 4 °C. Subsequently, the cells were washed and immediately analyzed using flow cytometry. The FlowJo 9.6 software was used for data analyses (Tree Star Software, Ashland, CA).

#### 5.2.5 Cellular Imatinib Accumulation Assay

##### 5.2.5.1 Imatinib Extraction from Cells

K562 and K562-R cells (4 × 10^6^) were treated with 1 μM imatinib for 2 h. Prior to this, K562-R cells were preincubated with 10 μM of telmi-ester (**7b**) and telmi-amide (**7c**) for 1 h. After treatment, cells were washed, and the resulting cell pellets were lysed in 200 μL of ice-cold methanol. Lysates were incubated on a tube shaker (Thermomixer C, Eppendorf) for 20 min at 4 °C and 1400 rpm, followed by centrifugation (MicroStar 17R, VWR) at 16000*g* for 5 min at 4 °C. 180 μL of supernatants were separated from the sediment and dried in a rotary vacuum evaporator (Concentrator plus, Eppendorf) and stored at −80 °C until analysis.

##### 5.2.5.2 U(H)PLC-SIM-MS

Dried cell extracts containing imatinib were resuspended in 50 μL of 0.1% formic acid, further diluted 1:10 in 0.1% formic acid, and analyzed on an U(H)PLC system (VanquishFlex, Thermo Fischer Scientific) using an ACQUITY UPLC HSS T3 column (2.1 × 50 mm, 1.8 μm, Waters, buffer A: H20, 0.05% difluoroacetic acid (DFA), buffer B: 80% acetonitrile, 0.05% DFA) at a flow rate of 800 μL/min, and a column temperature of 35 °C. Metabolites were separated using the following gradient: 1% B for 0.2 min, 1% B to 70% B within 0.6 min, 70% B to 90% B within 0.2 min, 90% B for 0.3 min. The UPLC system was coupled to a quadrupole orbitrap mass spectrometer (Exploris 480, Thermo Fischer Scientific) via a HESI electrospray source. The MS system was operated in positive ion mode and imatinib was detected by single ion monitoring (SIM) using a centered mass of *m*/*z* = 494.2663 (quadrupole isolation window 10 *m*/*z*), a resolution of 15,000 at *m*/*z* 200, RF lens of 50%, AGC target of 100%, and a maximum injection time of 100 ms. The spray voltage was set to 3500 V and the ion transfer tube and vaporizer temperatures were set at 350 °C. Sheath gas was used at a flow rate of 60 (arbitrary units (a.u)) and auxiliary gas at a flow rate of 15 (a.u)).

Raw data processing was performed using TraceFinder General Quan 5.0 (Thermo Scientific, San Jose, CA, USA). The ICIS algorithm was used for peak identification and integration with the following parameters Peak detection strategy: Highest peak; Peak threshold type: Area; Threshold: 1; Smoothing: 3; S/N threshold: 3; Tailing Factor: 8. Peaks were manually adjusted when necessary. Data were further processed using R (version 4.4.1) and RStudio (version 2024.09.0 + 375) with the R package rstatix (https://cran.r-project.org/web/packages/rstatix/index.html).

#### 5.2.6 Colony Forming Cell (CFC) Assay

Frozen peripheral blood mononuclear cells (PBMC) from TKI naïve CML patients and healthy controls were used for the CFC assay as described previously.^46^ Briefly, thawed cells were resuspended a semisolid medium (Enriched MethocCult H4435, Stem Cell Technologies) supplemented with the respective compounds and cultured in small culture dishes for 2 weeks. Colonies were counted using an inverted microscope.

#### 5.2.7 Physicochemical and ADME Assays

##### 5.2.7.1 Turbidimetric Aqueous Solubility

To assess the aqueous solubility, the compounds were diluted in buffer (0.01 M phosphate buffered saline pH 7.4) at concentrations ranging from 1 to 100 μM (final DMSO concentration 1%). After incubation at 37 °C for 2 h, the absorbance was measured at 620 nm and the solubility was estimated based on the concentration of the compound causing an absorbance increase compared to the vehicle control (1% DMSO in buffer). Nicardipine and pyrene were used as control compounds.

##### 5.2.7.2 Microsomal Metabolic Stability

The stability of the compounds was assessed in pooled human and mouse liver microsomes (final protein concentration 0.5 mg/mL). The compounds (1 μM in 0.1 M phosphate buffer pH 7.4, final DMSO concentration 0.25%) were preincubated with the microsomes at 37 °C prior to the addition of NADPH. NADPH was added (final concentration 1 mM) and it was incubated for 0, 5, 15, 30, and 45 min (control without NADPH only for 45 min). Two control compounds were included for each species (mouse: diazepam and diphenhydramine; human: dextromethorphan and verapamil). At the appropriate time points, the reactions were stopped by transferring into acetonitrile. After protein precipitation, by centrifugation at 3000 rpm for 20 min at 4 °C, the sample supernatants were analyzed using LC–MS/MS analysis (control compounds were included). The elimination rate constant was determined from the ratio of the ln peak area to time. Half-life (*t*_1/2_) and intrinsic clearance (CL_int_) were calculated using the appropriate formulas.

##### 5.2.7.3 Hepatocyte Stability

Cryopreserved hepatocytes (human and mouse) were used to analyze the stability of the compounds. Hepatocytes (0.5 × 10^6^ cells/mL in Williams E medium supplemented with 2 mM l-glutamine and 25 mM *N*-2-hydroxyethylpiperazin-*Ń*-ethanesulfonic acid (HEPES)) were preincubated at 37 °C. The compounds were added (1 μM, final DMSO concentration 0.25%) and it was incubated at 37 °C for 0, 5, 10, 20, 40, or 60 min. Two control compounds were included for each species (mouse and human: verapamil and raloxifene, respectively). At the appropriate time points, the reactions were stopped by transferring into acetonitrile. After protein precipitation by centrifugation at 3000 rpm for 20 min at 4 °C, the sample supernatants were analyzed by LC–MS/MS analysis (control compounds were included). The elimination rate constant was determined from the ratio of the ln peak area to time. Half-life (*t*_1/2_) and intrinsic clearance (CL_int_) were calculated using the appropriate formulas.

##### 5.2.7.4 Caco2 Permeability (Bidirectional)

To assess intestinal permeability, Caco2 cells (ATCC; RRID/CVCL_0025) were seeded onto transwell plates at 1 × 10^5^ cells/cm^2^ in DMEM and incubated at 37 °C (5% CO_2_, relative humidity 95%). The medium was changed regularly and on day 20 the permeability study was performed. The monolayers were prepared by rinsing the apical and basolateral surfaces with Hanks Balanced Salt Solution (HBSS) twice at the desired pH and by incubating for 40 min to stabilize the physiological parameters. Lucifer yellow integrity monitoring ensured monolayer quality. The compounds were diluted with buffer to a concentration of 10 μM (final DMSO concentration ≤1%) and added to the apical or basolateral sides of the cell monolayer. Compound permeability was assessed in duplicates and molecules of known permeability characteristics were used as controls (antipyrine, atenolol, talinolol, and estrone 3-sulfate). To calculate P_app_, compound appearance was measured using LC–MS/MS. The efflux ratio was determined for bidirectional assays.

#### 5.2.8 In Vivo Studies Using a Human CML Xenograft Model

Six-week-old female NOD/SCID mice (Charles River Laboratories) were housed in individually ventilated cages in a hygienic environment with controlled temperature (20–24 °C), humidity (30–70%), and 12 h light/dark cycles. Free access to sterilized standard lab diet and autoclaved tap water was granted for the duration of the study. All aspects of this work, including housing, experimentation, and disposal of animals were performed in general accordance with the Guide for the Care and Use of Laboratory Animals: Eighth Edition (National Academy Press, Washington, D. C., 2011). The study was performed in the AAALAC accredited laboratory under the supervision of the veterinarians and the animal care and use protocol was approved by the IACUC at PDS. K562-R cells were used to establish the human CML xenograft model. The NOD/SCID mice were subcutaneously (SC) implanted with viable tumor cells, 0.2 mL/1 × 10^7^ cells per animal with 50% high concentration Matrigel (354248, Corning, USA) into the left flank of each animal. 10% dimethyl sulfoxide (DMSO; Cat# 51779)/10% Solutol HS-15 (V00001, BASF, Germany)/80% water for injection (WFI) was used according to the solubility assessments that were carried out for formulation of **7c** (telmi-amide) in different vehicles (data not shown). Imatinib was formulated in 100% WFI. Fourteen days posttumor cell implantation, when the grouped mean tumor volume reached 148–149 mm^3^ (denoted as day 1), the animals were randomized into four efficacy treatment groups with seven animals per group followed by treatment initiation. The dosing solutions were orally (PO) administered and the dosing volume was 10 mL/kg for all treatment groups (for study design, see Table S1):

- Group 1: The vehicle, 10% DMSO/10% Solutol HS-15/80% WFI, was PO administered once daily for 30 consecutive days (QD × 30 days) with a total of 30 dose administrations, starting on day 1.
- Group 2: Imatinib at 50 mg/kg, was formulated in WFI and PO administered QD × 30 days, starting on day 1.
- Group 3: **7c** at 10 mg/kg, was formulated in 10% DMSO/10% Solutol HS-15/80% WFI and PO administered QD × 30 days, starting on day 1.
- Group 4: Combination therapy with imatinib at 50 mg/kg and **7c** at 10 mg/kg. Both compounds were PO administered for QD × 30 days, starting on day 1. On each dosing day, imatinib was PO administered followed by TA-1 PO administration within 1 h.

Palpable tumor volumes and body weights of the animals were measured and recorded twice a week during the study period. The length and width of the tumors were measured using a caliper. The length represents the largest tumor diameter, and the width represents the perpendicular tumor diameter. Tumor volume was calculated according to the prolate ellipsoid formula: Tumor volume = length × (width)^2^ × 0.5. The animals were checked daily for humane end points. Body weight was measured and recorded daily after dosing. Euthanasia was performed following the PDS IACUC approved SOP “Euthanasia Working Instruction” (document QWCN38), which follows the 2020 AVMA Guidelines on Euthanasia. Animals were euthanized using compressed CO_2_ gas in a CO_2_ gas chamber. On day 33 posttumor measurement, the tumor samples were harvested from all animals, weighted, and photographed.

The % T/C value, defined as the ratio of the mean tumor volume for the treated versus control group, was calculated as % T/C = (T*n*/C*n*) × 100%; whereas T*n*: denotes mean tumor volume of the treated group on day *n*, and C*n*: denotes mean tumor volume of the control group on day *n*. The optimal value is the minimal % T/C ratio, which reflects the maximal tumor growth inhibition achieved. A % T/C value ≤42% compared to that of the vehicle control group was considered significant antitumor activity following the guideline of NCI.^34^

TGI (Tumor growth inhibition) was calculated by the following formula
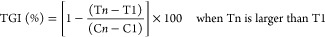

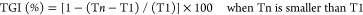


Whereas: T*n*: denotes the mean tumor volume of the treated group on day *n*; T1: denotes the mean tumor volume of the treated group on day 1; C*n*: denotes the mean tumor volume of the vehicle control group on day *n*; and C1: denotes the mean tumor volume of the vehicle control group at day 1. TGI % = 100% represents complete tumor growth inhibition.

## Supporting Information Available

Ancillary information: the below list indicates what is included in the Supporting Information. Synthesis of the intermediates. NMR spectra of target compounds. HPLC chromatograms of target compounds. HRMS spectra of target compounds. Biological data: on the repression of the CSC-defining side population in TKI-resistant CML cells; on the suppression of colony formation of primary CML cells and the inhibition of tumor growth; on the efficient targeting of the CSC pool of solid cancers (PDF)
Table S1_CML xenograft model.xlsx (XLSX)
Molecular Formula Strings (CSV)
