# Supplementary material for: Eradication of Therapy-Resistant Cancer Stem Cells by Novel Telmisartan Derivatives
Source: J Med Chem. 2024 Dec 18;68(1):287–306. doi: 10.1021/acs.jmedchem.4c01865 (PMC11726677; doi:10.1021/acs.jmedchem.4c01865)
Supplement: Supplementary file 1 — jm4c01865_si_001.pdf [file jm4c01865_si_001.pdf]

Supporting Information

Eradication of Therapy-Resistant Cancer Stem Cells by Novel Telmisartan Derivatives

Anna M. Schoepf[1], Maximilian Gebhart[1], Martin Federspiel[1], Isabel Heidegger[2], Martin Puhr[2], Madlen Hotze[3], Marcel Kwiatkowski[3], Andreas Pircher[4], Dominik Wolf[4], Sieghart Sopper[4], Ronald Gust[1,#], Stefan Salcher[4,#,*]

[1] Department of Pharmaceutical Chemistry, Institute of Pharmacy, CMBI - Center for Molecular Biosciences Innsbruck, University of Innsbruck, CCB - Centrum for Chemistry and Biomedicine, 6020 Innsbruck, Austria

[2] Department of Urology, Medical University of Innsbruck, 6020 Innsbruck, Austria

[3] Department of Biochemistry and Center for Molecular Biosciences Innsbruck, University of Innsbruck, 6020 Innsbruck, Austria

[4] Department of Internal Medicine V, Hematology and Oncology, Tyrolean Cancer Research Institute (TKFI), Comprehensive Cancer Center Innsbruck (CCCI), Medical University of Innsbruck, 6020 Innsbruck, Austria

# These authors contributed equally

* Corresponding author, E-mail: stefan.salcher@i-med.ac.at

Table of contents

1. Synthesis 3
2. NMR spectra 7
3. HPLC chromatograms 27
4. HRMS spectra 37
5. Biological data 44

1. Synthesis

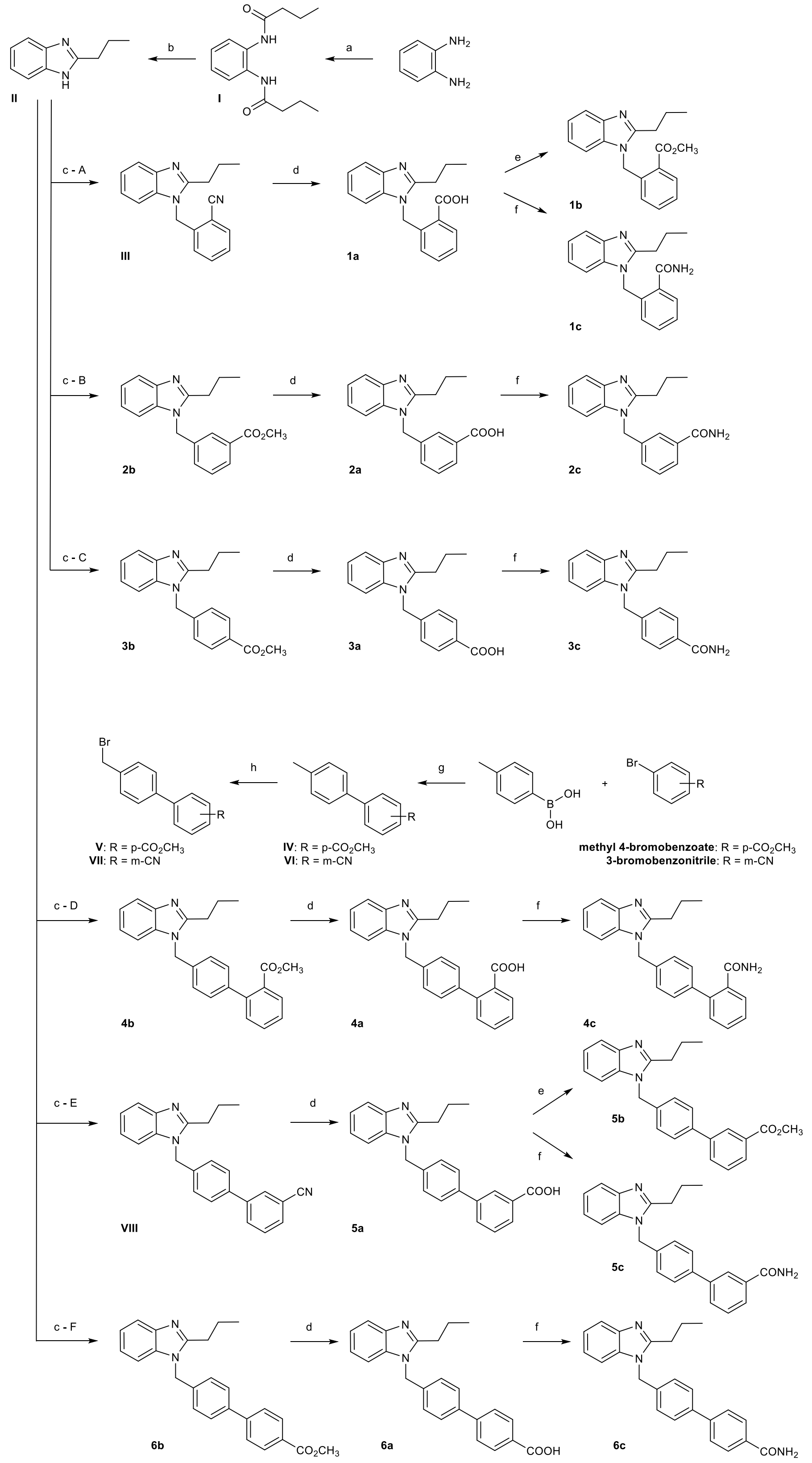

Scheme S1. Overview of the synthesis path. Reagents and conditions: (a) butyric anhydride, conc. HCl, 60 °C; (b) 4 N HCl, 100 °C; (c) A: 2-(bromomethyl)benzonitrile, B: methyl 3-(bromomethyl)benzoate, C: methyl 4-(bromomethyl)benzoate, D: methyl 4'-(bromomethyl)-[1,1'-biphenyl]-2-carboxylate, E: 4'-(Bromomethyl)-[1,1'-biphenyl]-3-carbonitrile, or F: methyl 4'-(bromomethyl)-[1,1'-biphenyl]-4-carboxylate, NaH, anhyd. DMF, 0 °C to rt; (d) KOH, EG, H_2O, 160 °C; (e) $SOCl_2$, anhyd. MeOH, 0 °C to rt; (f) PyBOP, NH_4Cl, DIPEA, DMF, 0 °C to rt; (g) $Pd(PPh_3)_4$, 2 N Na_2CO_3, dioxane 100 °C; (h) NBS, benzoyl peroxide, anhyd. CCl_4, 77 °C.

Synthesis of the intermediates

*N,N'-(1,2-Phenylene)dibutyramide (**I**)*

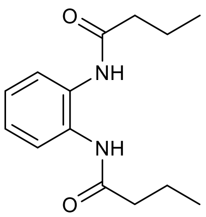

1,2-Phenylenediamine (10.0 g, 92.5 mmol) was suspended in butyric anhydride (43.7 g, 277.0 mmol) and treated with a few drops of conc. HCl. The reaction mixture was heated at 60 °C for 1.5 h. After adding ice water, the solution was basified to a pH of 9 with 6 N NaOH and extracted with DCM (3×). The combined organic phases were washed with brine, dried over anhyd. Na_2SO_4, filtered and the solvent was removed under reduced pressure. Off-white solid, yield: 83%. ^{1}H-NMR (400 MHz, DMSO-*d*$_6$): δ 9.32 (s, 2H, *NH*), 7.50 (dd, ^3J = 6.1 Hz, ^4J = 3.6 Hz, 2H, *H3, H6*), 7.13 (dd, ^3J = 6.1 Hz, ^4J = 3.5 Hz, 2H, *H4*, *H5*), 2.30 (t, ^3J = 7.4 Hz, 4H, ($CH_2CH_2CH_3)_2$), 1.66-1.57 (m, 4H, ($CH_2CH_2CH_3)_2$), 0.93 (t, ^3J = 7.4 Hz, 6H, ($CH_2CH_2CH_3)_2$).

*2-Propyl-1H-benzo[d]imidazole (**II**)*

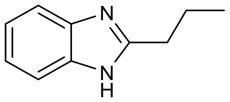

Intermediate **I** (20.0 g, 80.5 mmol) was heated in 4 N HCl (100 ml) at 100 °C for 12 h. The reaction mixture was cooled in an ice bath and basified to a pH of 9 with 6 N NaOH. DCM was added and it was extracted with 1 N NaOH (3×). The organic phases were collected, washed with brine, dried over anhyd. Na_2SO_4, and filtered. DCM was removed under reduced pressure. The crude product was purified by flash column chromatography (PE/EA, 3:7). Beige solid, yield: 58%. ^{1}H-NMR (400 MHz, DMSO-*d*$_6$): δ 12.15 (br s, 1H, *NH*), 7.52-7.39 (m, 2H, *H4*, *H7*), 7.13-7.06 (m, 2H, *H5*, *H6*), 2.78 (t, ^3J = 7.5 Hz, 2H, CH_2CH_2CH), 1.85-1.72 (m, 2H, $CH_2CH_2CH_3$), 0.94 (t, ^3J = 7.4 Hz, 3H, $CH_2CH_2CH_3$).

*Methyl 4'-methyl-[1,1'-biphenyl]-4-carboxylate (**IV**)*

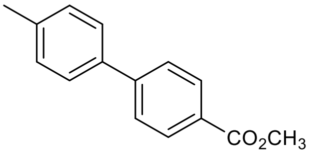

Methyl 4-bromobenzoate (4.0 g, 18.6 mmol) was dissolved in dioxane (40 ml) and 4-tolylboronic acid (3.8 g, 27.9 mmol), $Pd(PPh_3)_4$ (1.1 g, 0.9 mmol), and Na_2CO_3 (2.1 g, 20.0 mmol) were added. The reaction mixture was heated at 100 °C for 5 h. Then, dioxane was removed under reduced pressure and the residue was treated with ice water. It was neutralized with 1 N HCl and extracted with DCM (3×). The organic phases were collected, washed with brine, dried over anhyd. Na_2SO_4, and filtered. DCM was removed under reduced pressure. The crude product was purified by flash column chromatography with stepwise gradient elution (100% PE to PE/EA 8:2). Colorless solid, yield: 63%. ^{1}H-NMR (400 MHz, acetone-*d*$_6$): δ 8.07 (d, ^3J = 8.5 Hz, 2H, *Ar-H*), 7.78 (d, ^3J = 8.5 Hz, 2H, *Ar-H*), 7.63 (d, ^3J = 8.2 Hz, 2H, *Ar-H*), 7.32 (dd, ^3J = 8.6 Hz, ^4J = 0.7 Hz, 2H, *Ar-H*), 3.90 (s, 3H, CO_2CH_3), 2.38 (s, 3H, CH_3).

*Methyl 4'-(bromomethyl)-[1,1'-biphenyl]-4-carboxylate (**V**)*

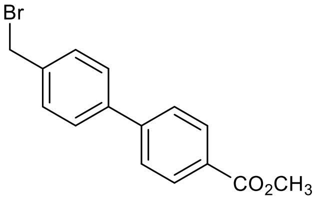

Intermediate **IV** (2.0 g, 8.8 mmol) was dissolved in anhyd. CCl_4 (50 ml) and NBS (1.7 g, 9.7 mmol) as well as benzoyl peroxide (0.1 g, 0.5 mmol) were added under an argon atmosphere. After heating at 77 °C for 7 h, the reaction mixture was cooled and extracted with saturated aqueous $Na_2S_2O_3$ solution (3×). The organic phases were collected, washed with brine, dried over anhyd. Na_2SO_4, and filtered. CCl_4 was removed under reduced pressure. The crude product was purified by flash column chromatography with stepwise gradient elution (PE/DE, 9:1 to 7:3). Colorless solid, yield: 44%. ^{1}H-NMR (400 MHz, acetone-*d$_6$*): δ 8.10 (d, ^3J = 8.5 Hz, 2H, *Ar-H*), 7.82 (d, ^3J = 8.5 Hz, 2H, *Ar-H*), 7.74 (d, ^3J = 8.3 Hz, 2H, *Ar-H*), 7.60 (d, ^3J = 8.3 Hz, 2H, *Ar-H*), 4.72 (s, 2H, *CH_2*), 3.91 (s, 3H, CO_2*CH_3*).

*4'-Methyl-[1,1'-biphenyl]-3-carbonitrile (**VI**)*

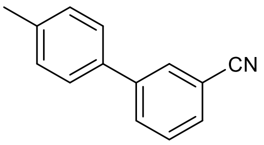

3-Bromobenzonitrile (3.0 g, 16.5 mmol) was dissolved in dioxane (30 ml) and 4-tolylboronic acid (3.4 g, 24.7 mmol), $Pd(PPh_3)_4$ (1.0 g, 0.8 mmol), and 2 N Na_2CO_3 (9 ml) were added. The reaction mixture was heated at 100 °C for 5 h. Then, dioxane was removed under reduced pressure and the residue was treated with ice water. It was neutralized with 1 N HCl and extracted with EA (3×). The organic phases were collected, washed with brine, dried over anhyd. Na_2SO_4, and filtered. EA was removed under reduced pressure. The crude product was purified by flash column chromatography with stepwise gradient elution (PE/EA, 9:1 to 7:3). Colorless solid, yield: 41%. ^{1}H-NMR (400 MHz, acetone-*d$_6$*): δ 8.05-8.00 (m, 1H, *Ar-H*), 8.00-7.94 (m, 1H, *Ar-H*), 7.77-7.70 (m, 1H, *Ar-H*), 7.70-7.59 (m, 3H, *Ar-H*), 7.32 (d, ^3J = 7.9 Hz, 2H, *Ar-H*), 2.38 (s, 3H, *CH_3*).

*4'-(Bromomethyl)-[1,1'-biphenyl]-3-carbonitrile (**VII**)*

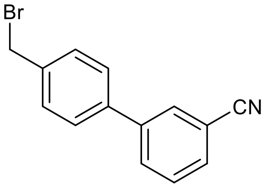

Intermediate **VI** (1.2 g, 6.2 mmol) was dissolved in anhyd. CCl_4 (40 ml) and NBS (1.2 g, 6.8 mmol) as well as benzoyl peroxide (0.1 g, 0.4 mmol) were added under an argon atmosphere. After heating at 77 °C for 5 h, the reaction mixture was cooled and extracted with saturated aqueous $Na_2S_2O_3$ solution (3×). The organic phases were collected, washed with brine, dried over anhyd. Na_2SO_4, and filtered. The solvent was removed under reduced pressure. The product was purified by flash column chromatography with stepwise gradient elution (PE/DE, 9:1 to 7:3). Colorless solid, yield: 61%. ^{1}H-NMR (400 MHz, acetone-*d$_6$*): δ 8.10-8.07 (m, 1H, *Ar-H*), 8.04-8.00 (m, 1H, *Ar-H*), 7.80-7.72 (m, 4H, *Ar-H*), 7.61 (d, ^3J = 8.4 Hz, 2H, *Ar-H*), 4.72 (s, 2H, *CH_2*).

General procedure for the *N*-alkylation

The solution of **II** (1 eq) in anhyd. DMF (≈1-2 ml/mmol) was cooled in an ice bath, treated with NaH (1.2 eq) in small portions, and stirred till the formation of hydrogen was completed. The respective (bromomethyl)aryl (1.1 eq) was added and the reaction mixture was stirred on ice for 30 min and then at rt for 12 h. Ice water was added to double the volume and the mixture was neutralized with 1 N HCl. Then, it was extracted with EA (3×), the organic layers were combined, washed with brine, dried over anhyd. Na_2SO_4, and filtered. The solvent was removed under reduced pressure and the crude product was purified by flash column chromatography with stepwise gradient elution (PE/EA, 7:3 to 3:7).

*2-((2-Propyl-1H-benzo[d]imidazol-1-yl)methyl)benzonitrile (**III**)*

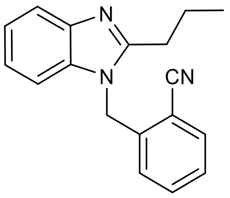

From **II** (1.0 g, 6.2 mmol) with NaH (0.2 g, 7.5 mmol) and 2-(bromomethyl)benzonitrile (1.4 g, 6.9 mmol) in anhyd. DMF (6 ml). Beige solid, yield: 87%. ^{1}H-NMR (400 MHz, DMSO-d_6): δ 7.93 (d, ^3J = 7.7 Hz, 1H, *H4'*), 7.65-7.55 (m, 2H, *H6, H6'*), 7.53-7.45 (m, 1H, *H5'*), 7.35 (d, ^3J = 7.1 Hz, 1H, *H3*), 7.22-7.10 (m, 2H, *H4, H5*), 6.69 (d, ^3J = 7.8 Hz, 1H, *H7'*), 5.71 (s, 2H, *NCH$_2$*), 2.79 (t, ^3J = 7.5 Hz, 2H, *CH$_2$*CH$_2$CH$_3$), 1.81-1.69 (m, 2H, CH$_2$*CH$_2$*CH$_3$), 0.94 (t, ^3J = 7.3 Hz, 3H, CH$_2$CH$_2$*CH$_3$*).

*4'-((2-Propyl-1H-benzo[d]imidazol-1-yl)methyl)-[1,1'-biphenyl]-3-carbonitrile (**VIII**)*

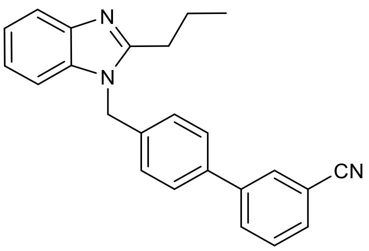

From **II** (0.6 g, 3.4 mmol) with NaH (0.1 g, 4.1 mmol) and 4'-(Bromomethyl)-[1,1'-biphenyl]-3-carbonitrile (1.0 g, 3.8 mmol) in anhyd. DMF (6 ml). Colorless solid, yield: 66%. ^{1}H-NMR (400 MHz, acetone-d_6): δ 8.06-8.02 (m, 1H, *Ar-H*), 8.00-7.95 (m, 1H, *Ar-H*), 7.78-7.58 (m, 5H, *Ar-H*), 7.43-7.36 (m, 1H, *Ar-H*), 7.26 (d, ^3J = 8.7 Hz, 2H, *Ar-H*), 7.22-7.13 (m, 2H, *Ar-H*), 5.59 (s, 2H, *NCH$_2$*), 2.89 (t, ^3J = 7.6 Hz, 2H, *CH$_2$*CH$_2$CH$_3$), 1.94-1.82 (m, 2H, CH$_2$*CH$_2$*CH$_3$), 1.01 (t, ^{3}J = 7.4 Hz, 3H, CH$_2$CH$_2$*CH$_3$*).

2. NMR spectra

Figure S1: ^{1}H- and ^{13}C-NMR spectra of compound **1a**.

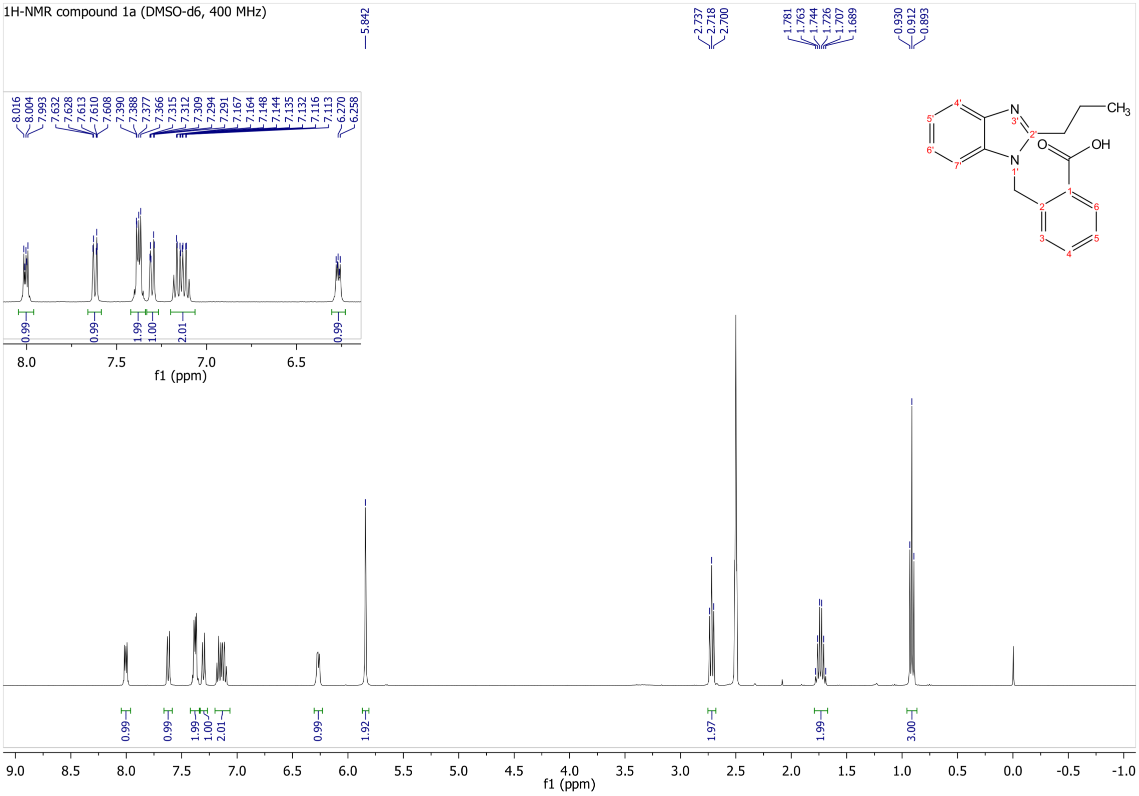

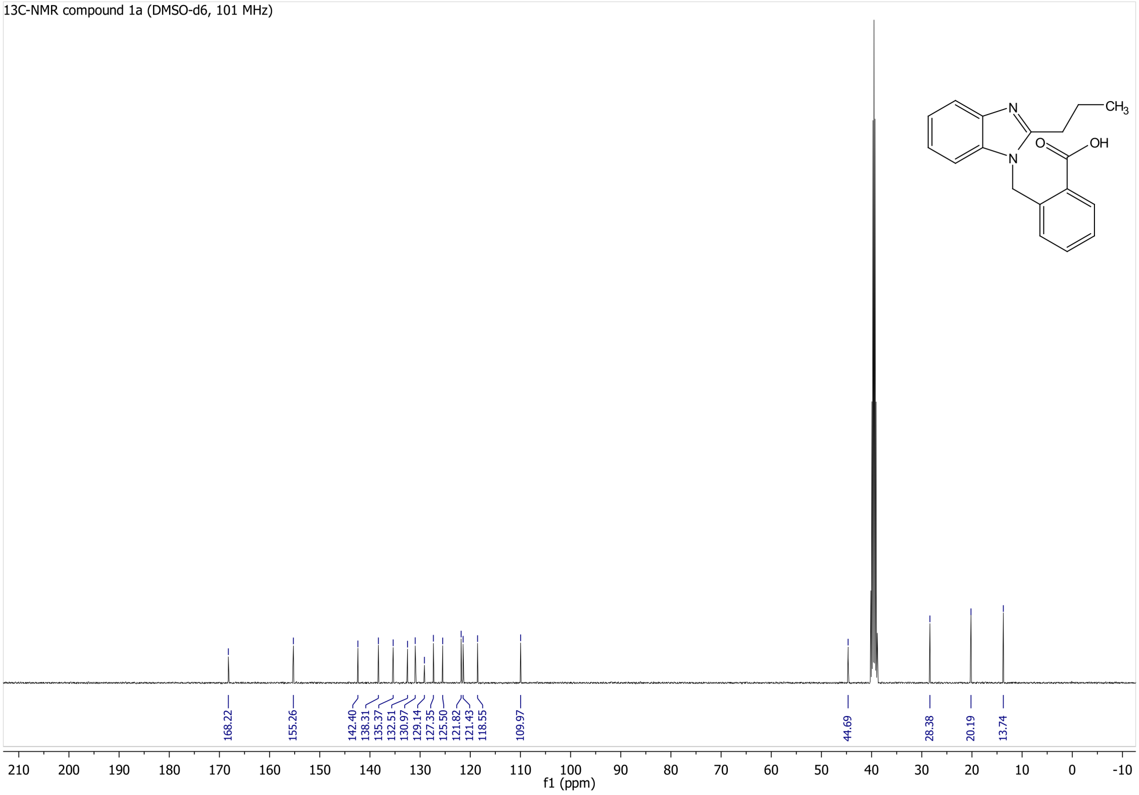

Figure S2: ^{1}H- and ^{13}C-NMR spectra of compound **1b**.

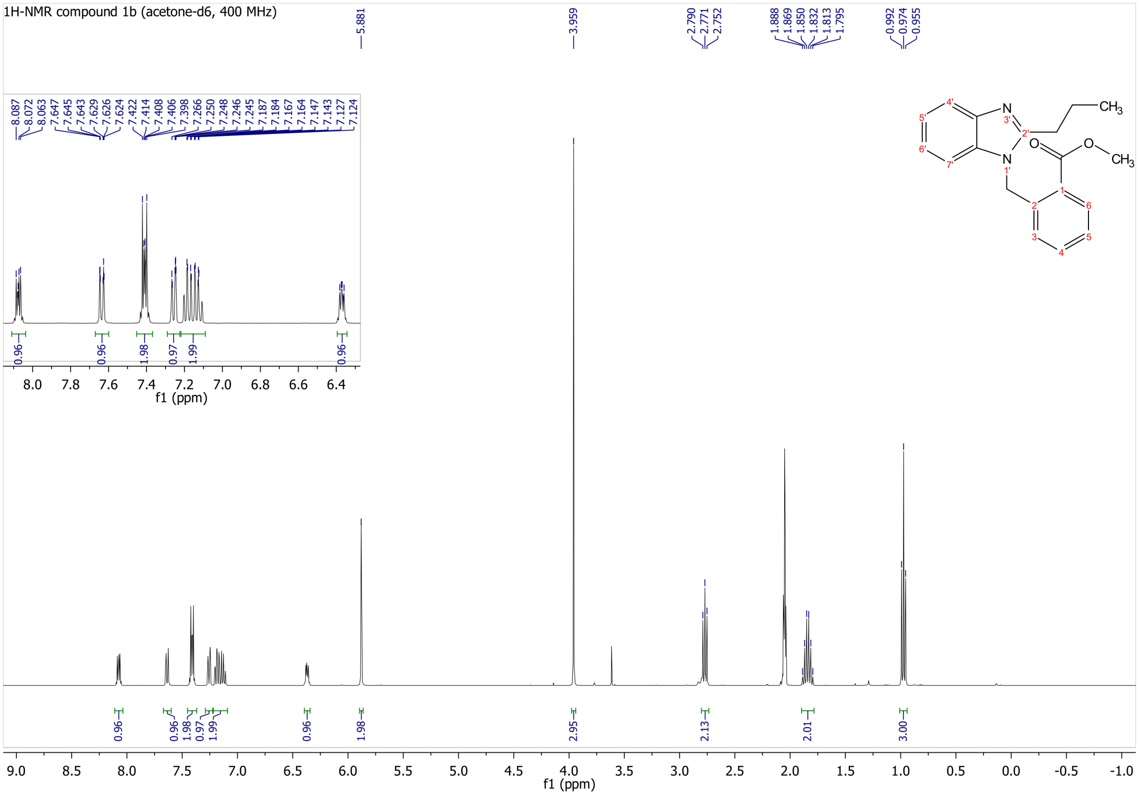

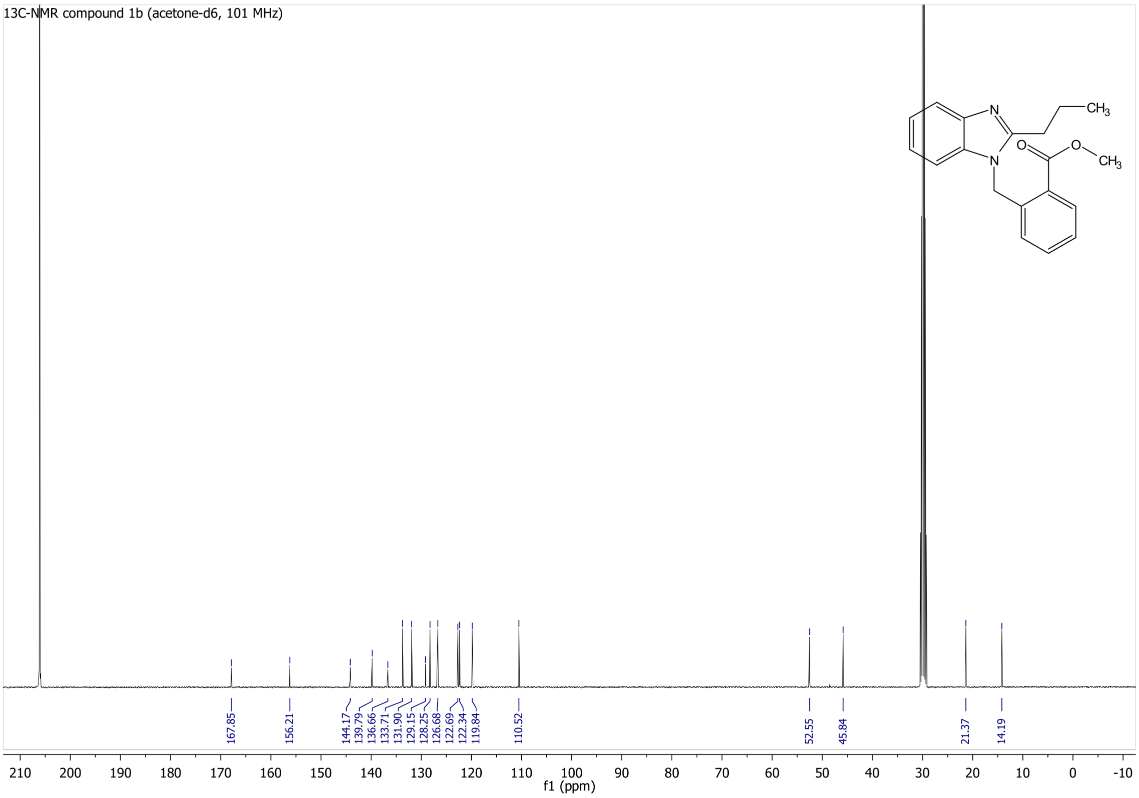

Figure S3: ^{1}H- and ^{13}C-NMR spectra of compound **1c**.

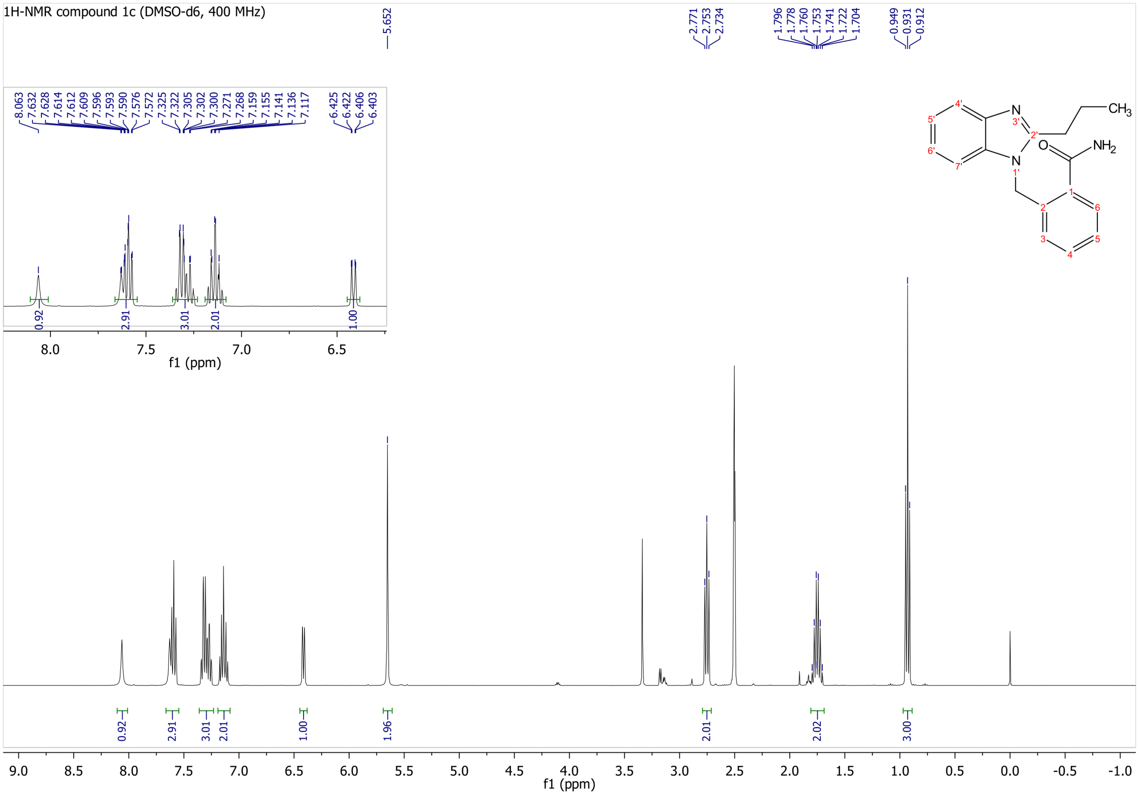

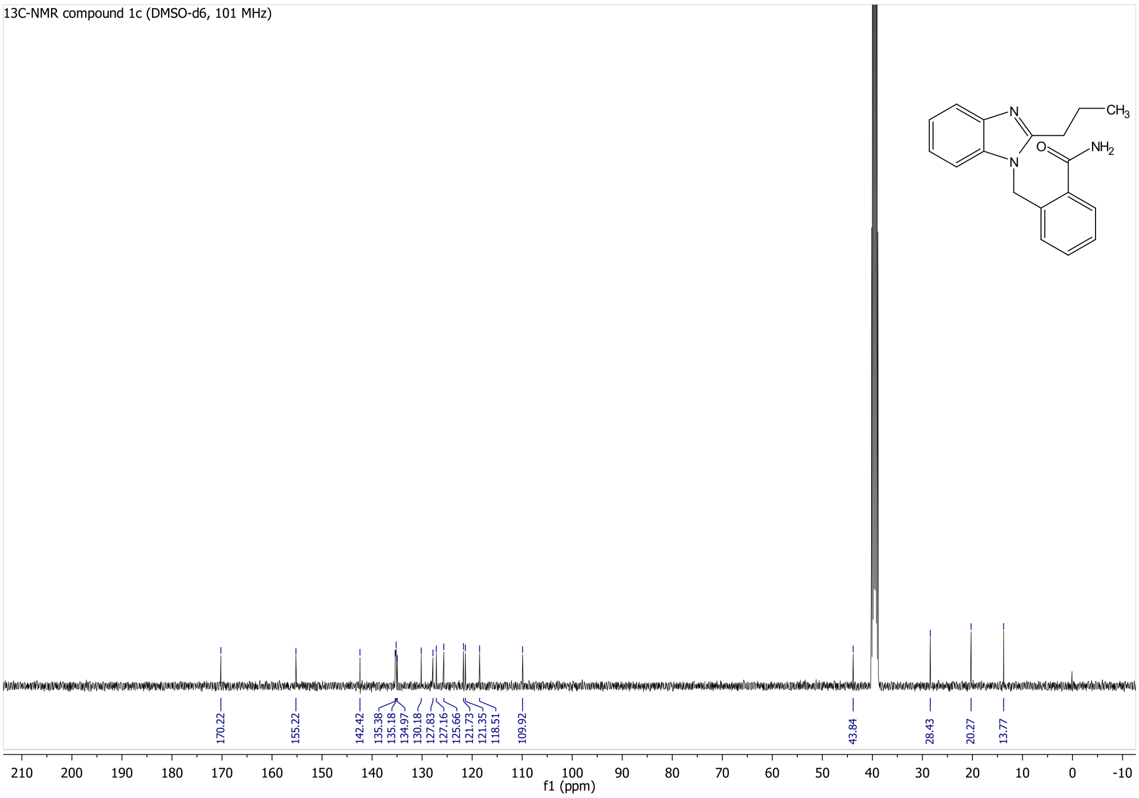

Figure S4: ^{1}H- and ^{13}C-NMR spectra of compound **2a**.

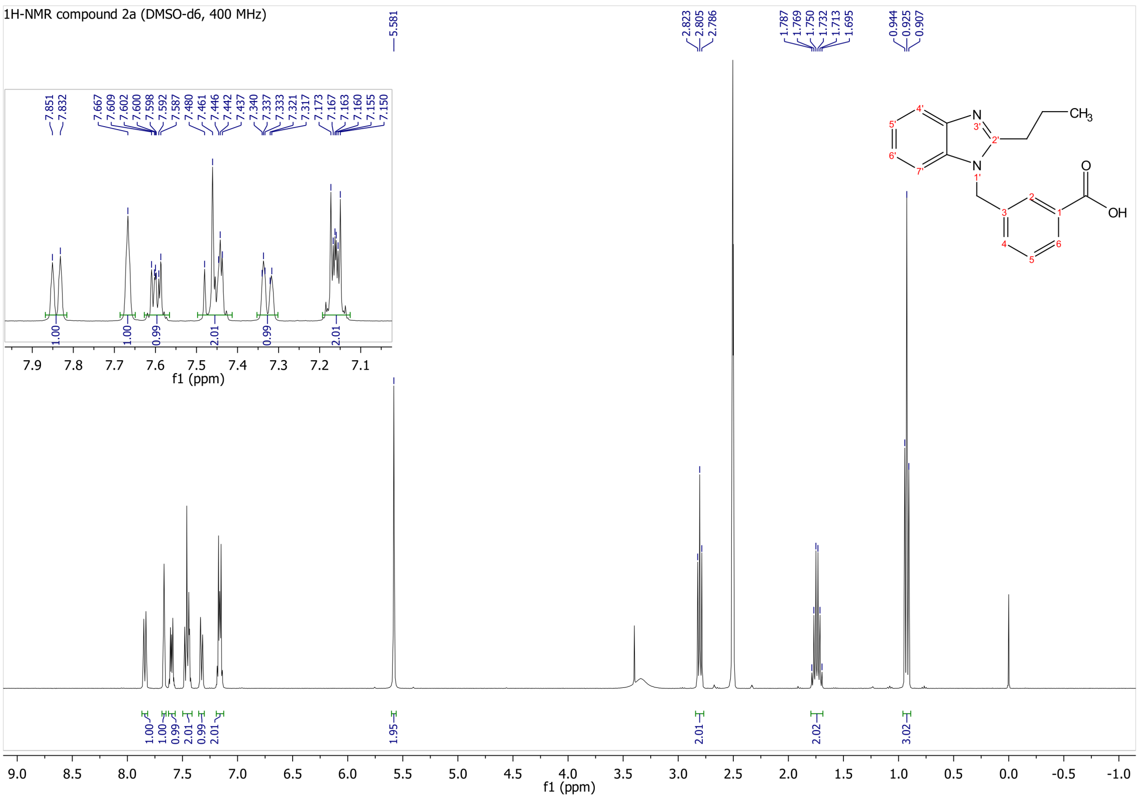

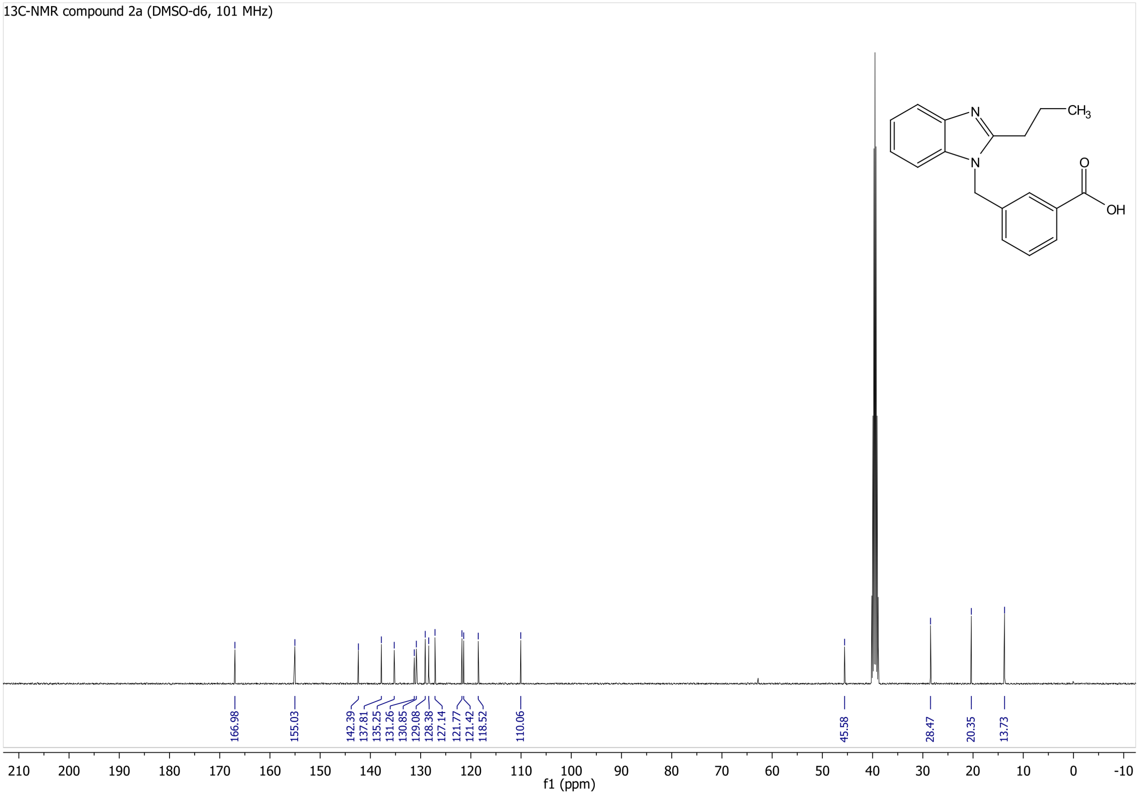

Figure S5: ^{1}H- and ^{13}C-NMR spectra of compound **2b**.

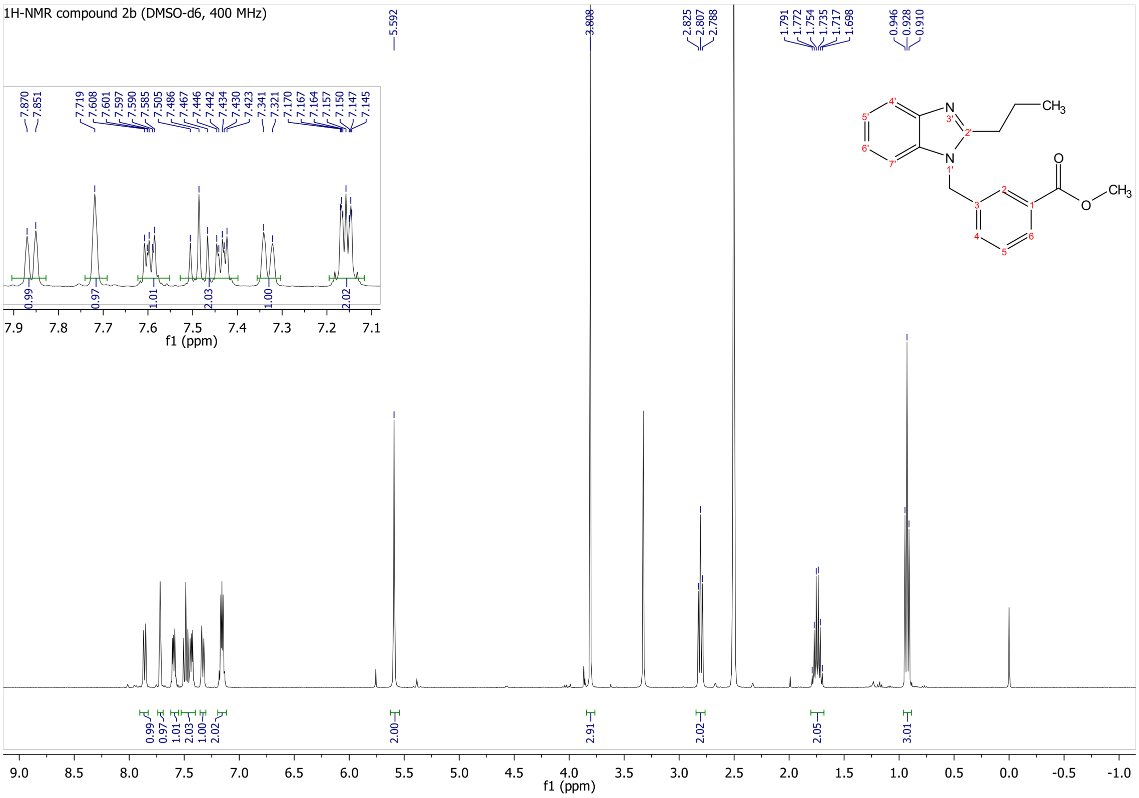

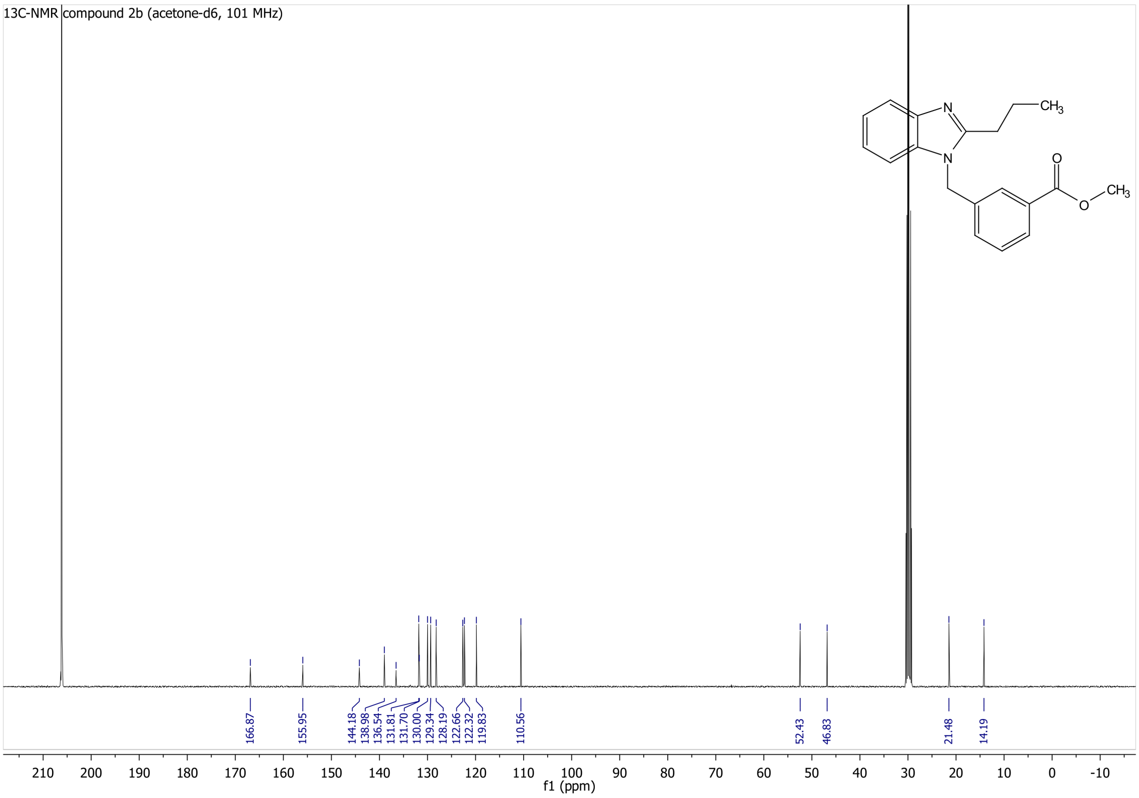

Figure S6: ^{1}H- and ^{13}C-NMR spectra of compound **2c**.

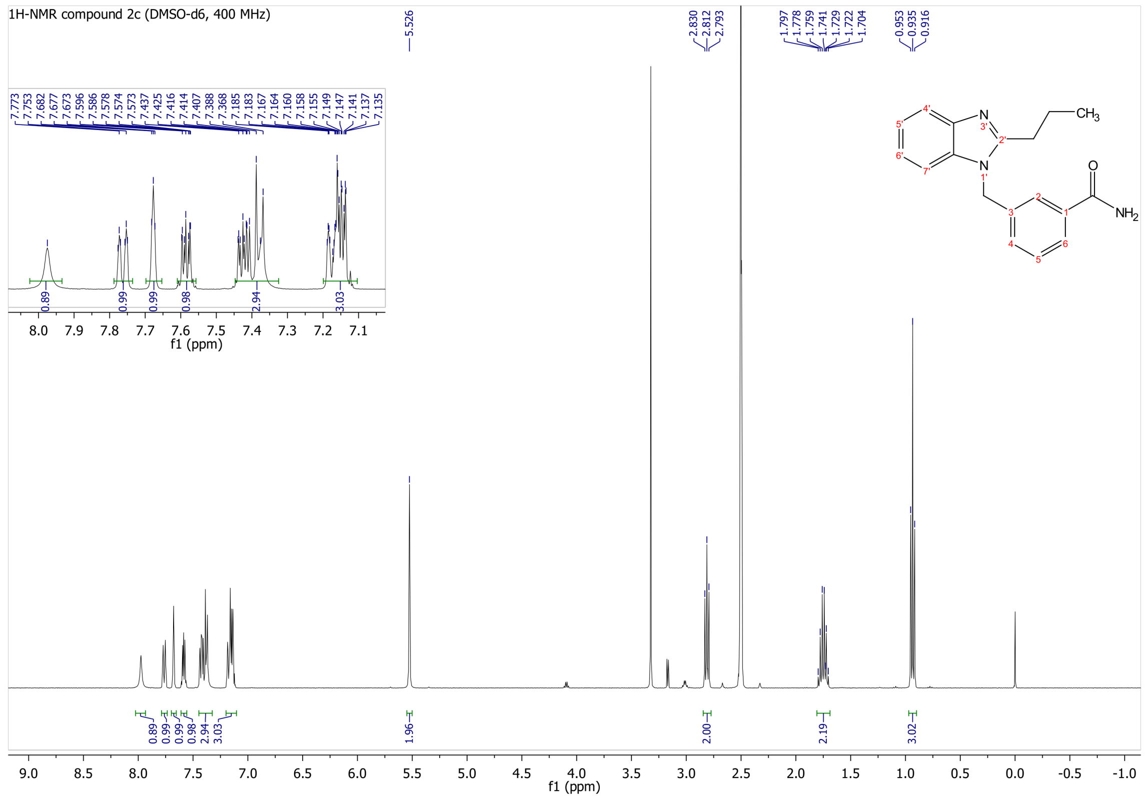

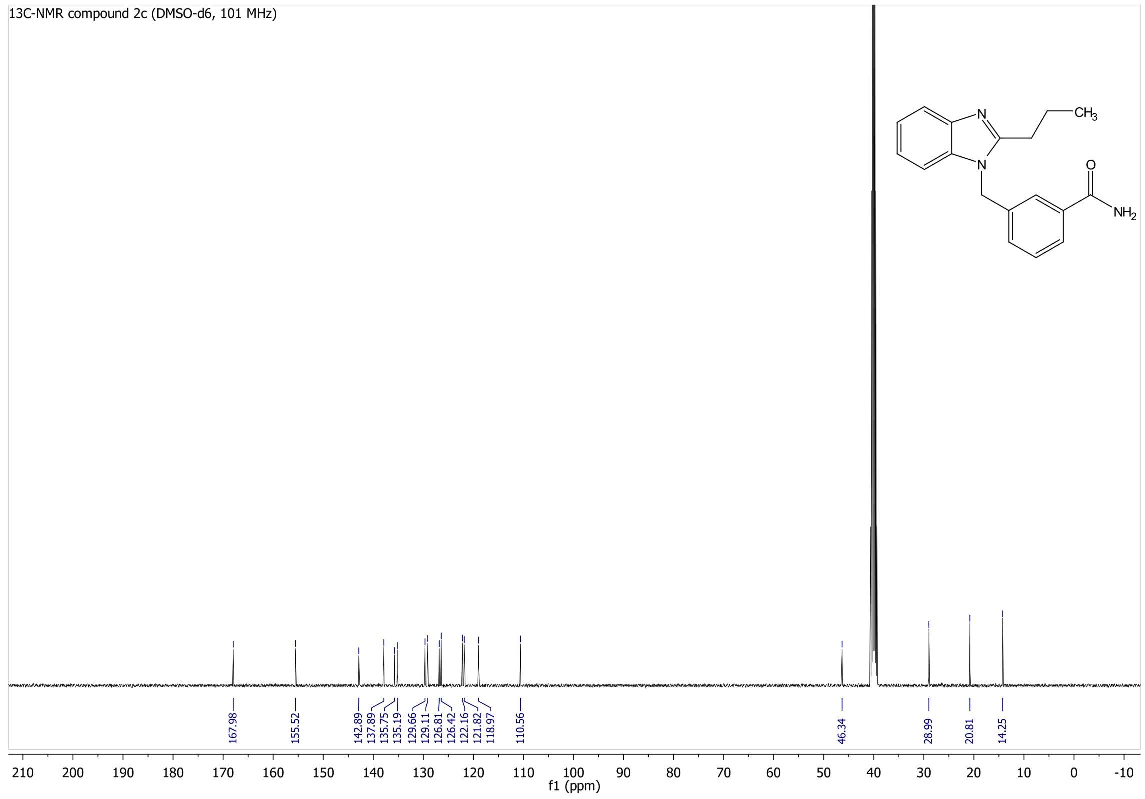

Figure S7: ^{1}H- and ^{13}C-NMR spectra of compound **3a**.

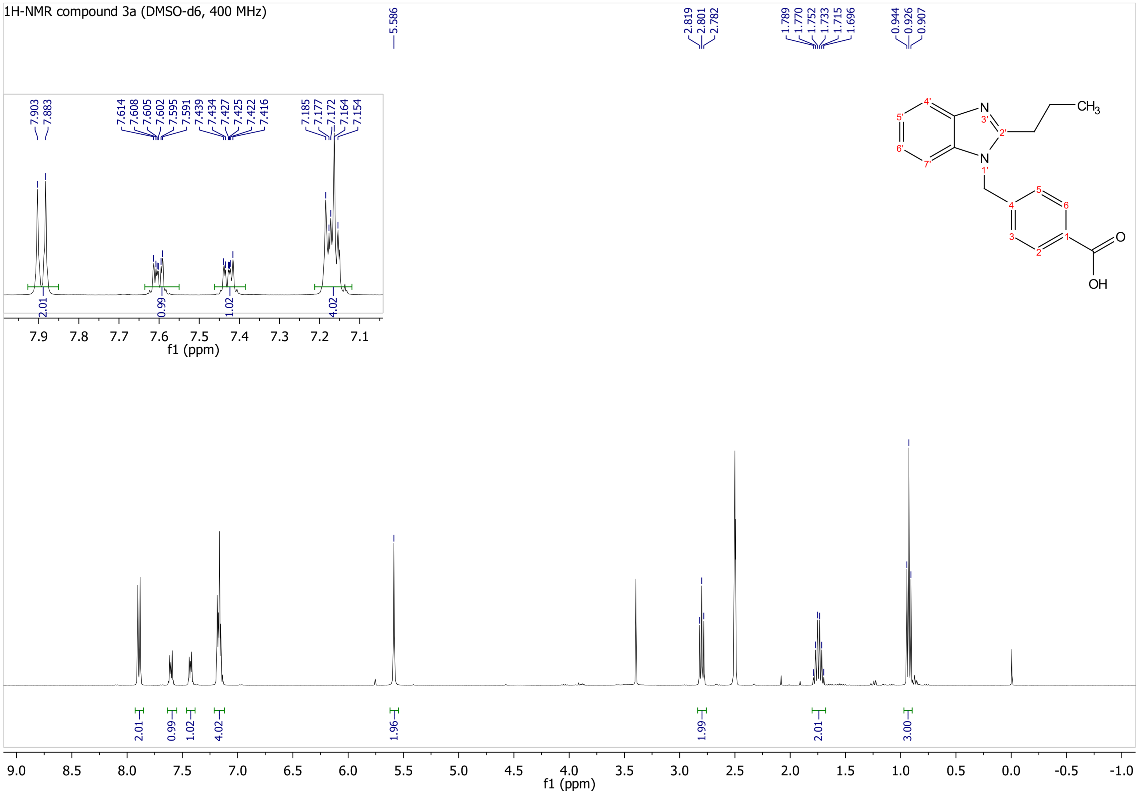

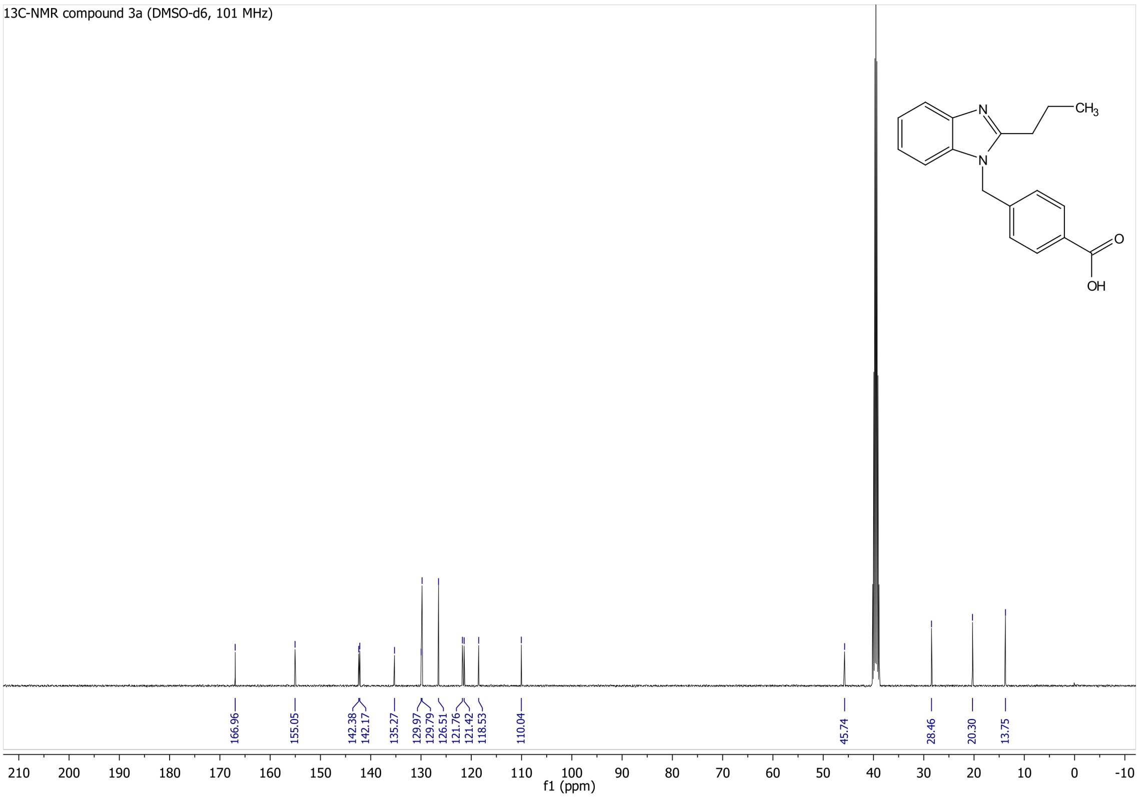

Figure S8: ^{1}H- and ^{13}C-NMR spectra of compound **3b**.

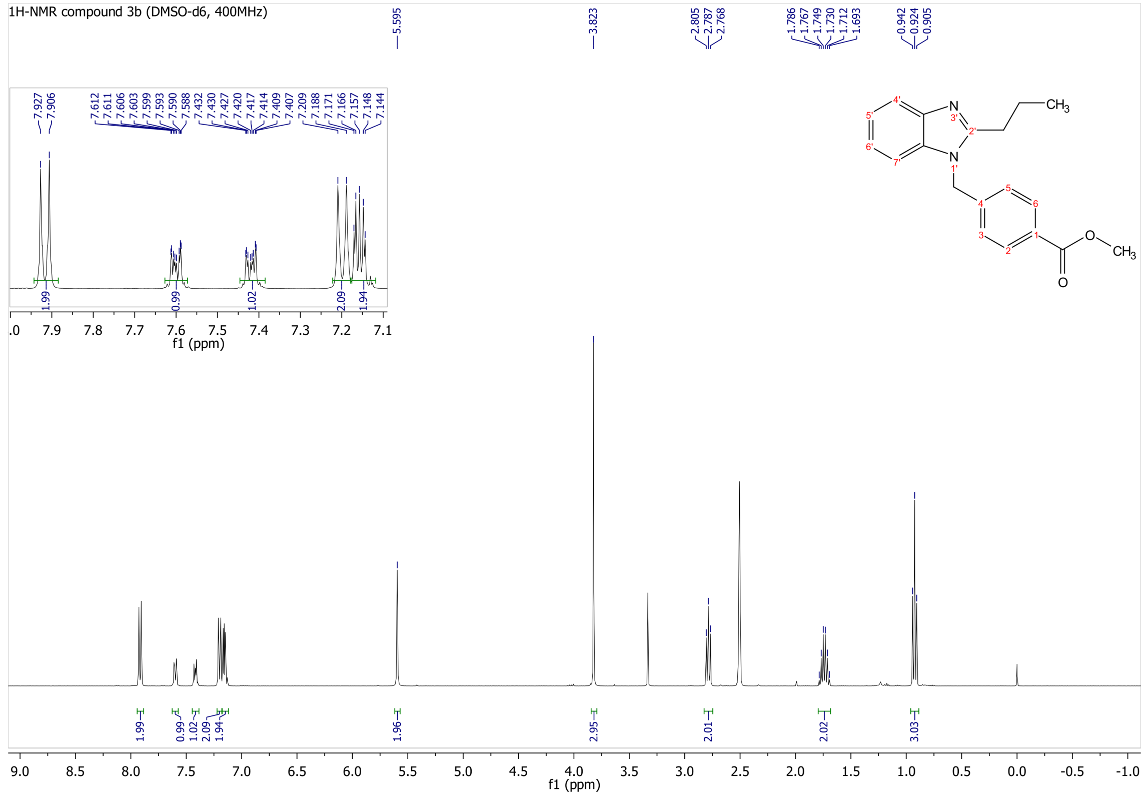

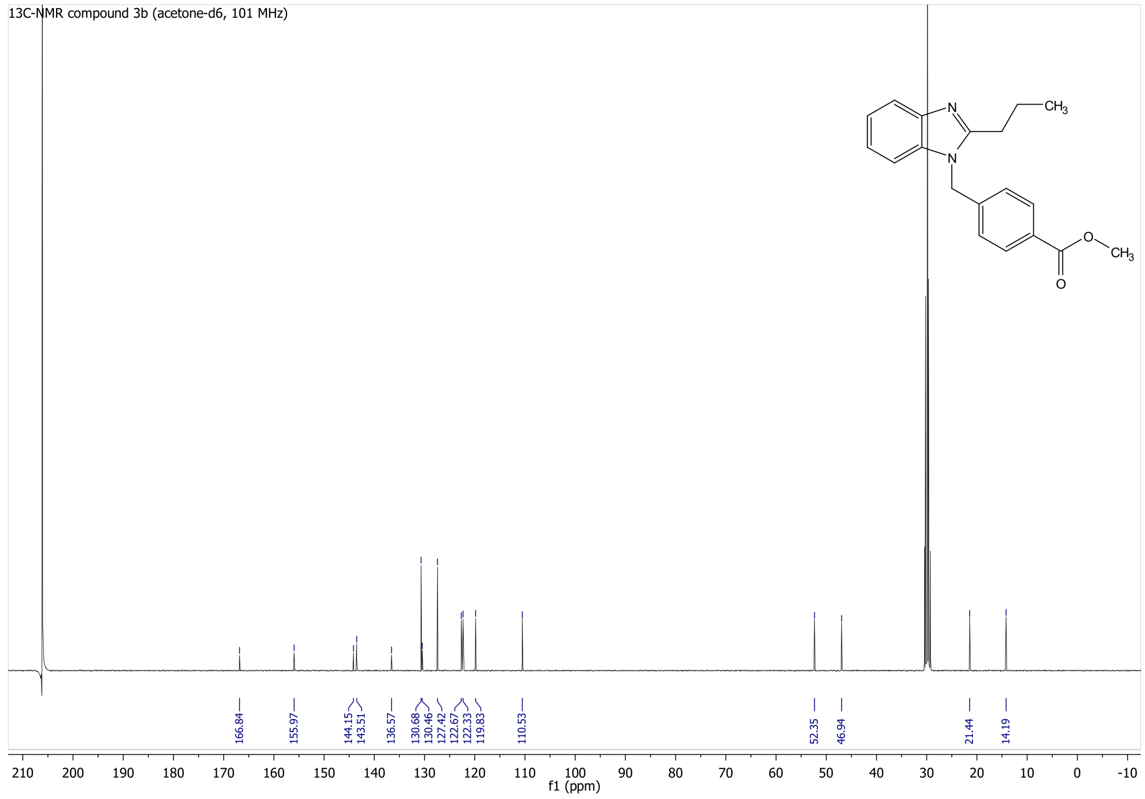

Figure S9: ^{1}H- and ^{13}C-NMR spectra of compound **3c**.

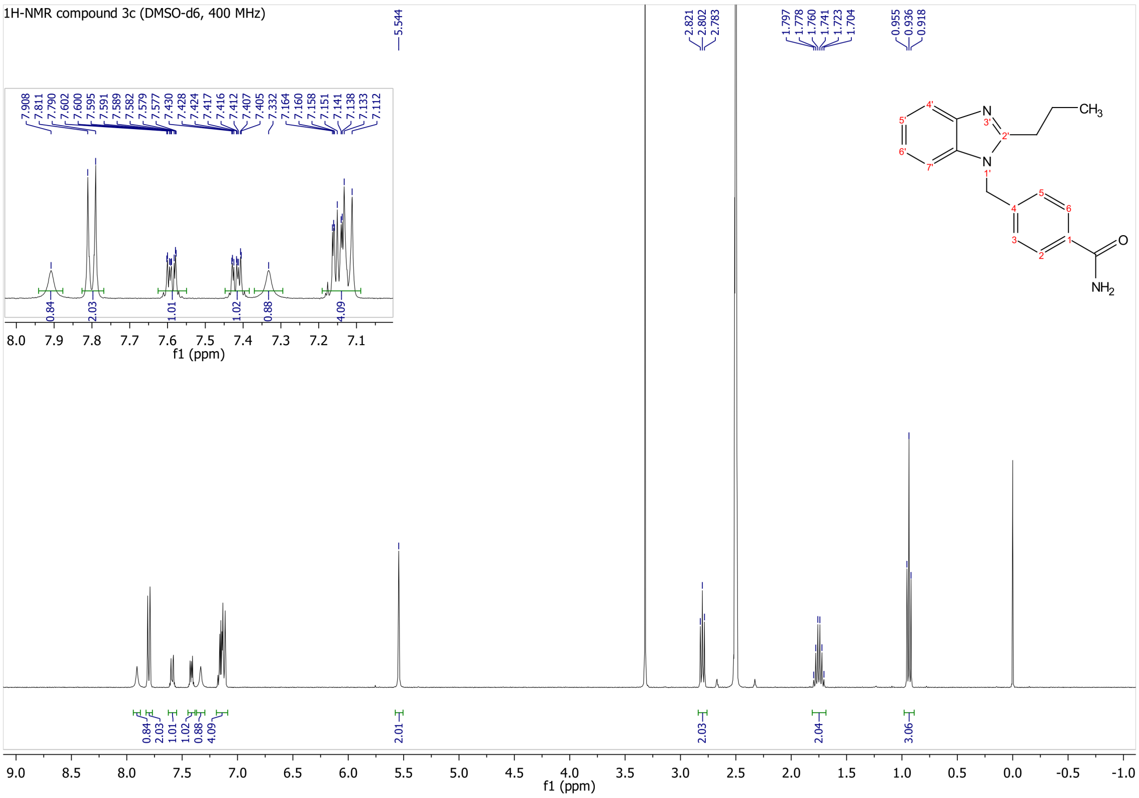

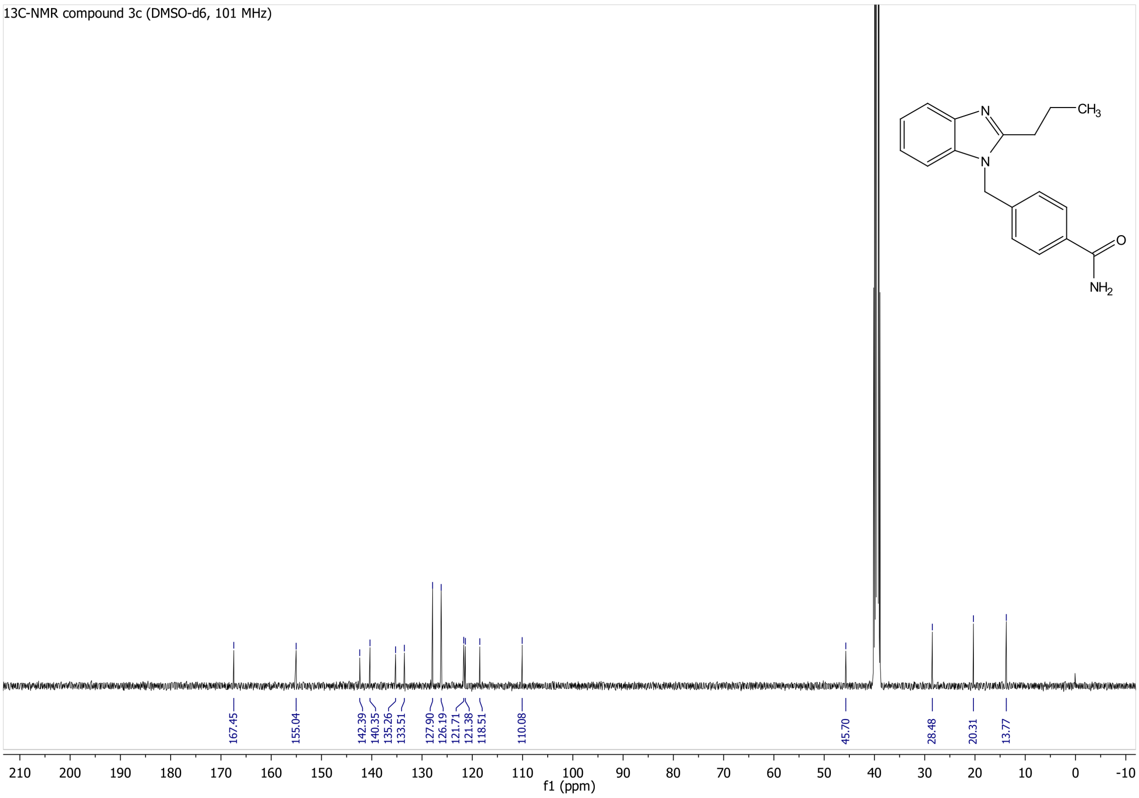

Figure S10: ^{1}H- and ^{13}C-NMR spectra of compound **4a**.

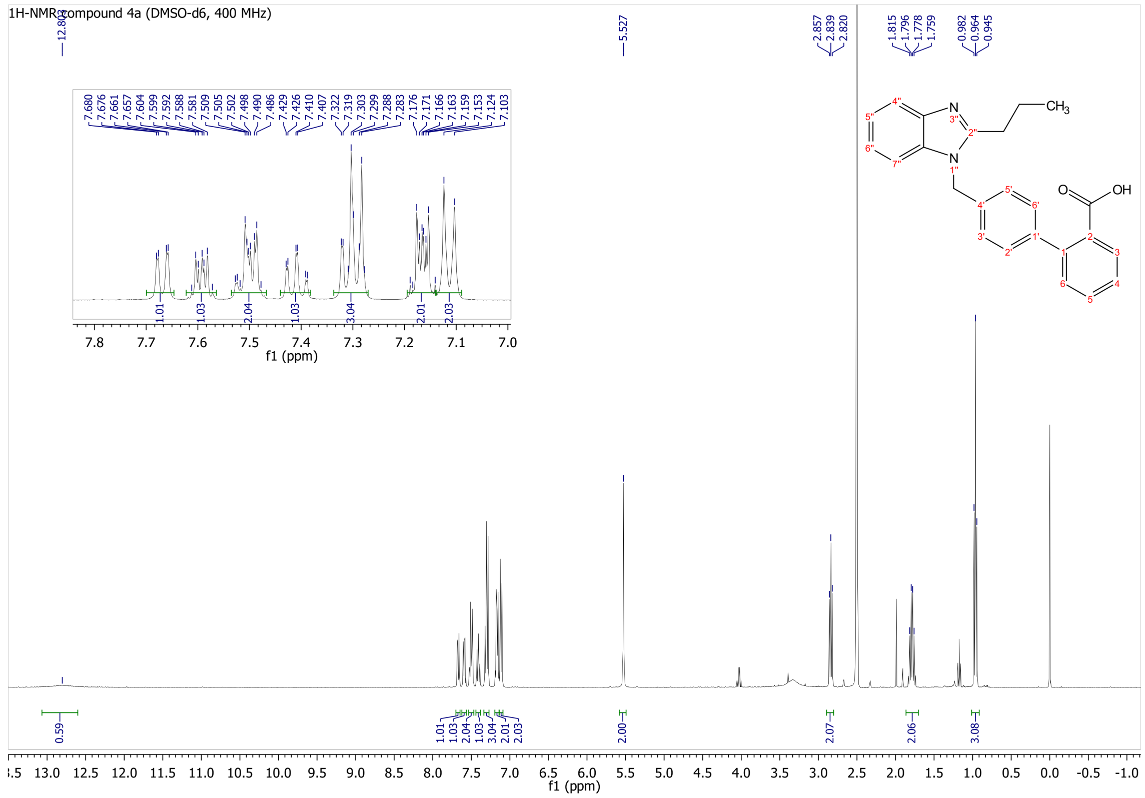

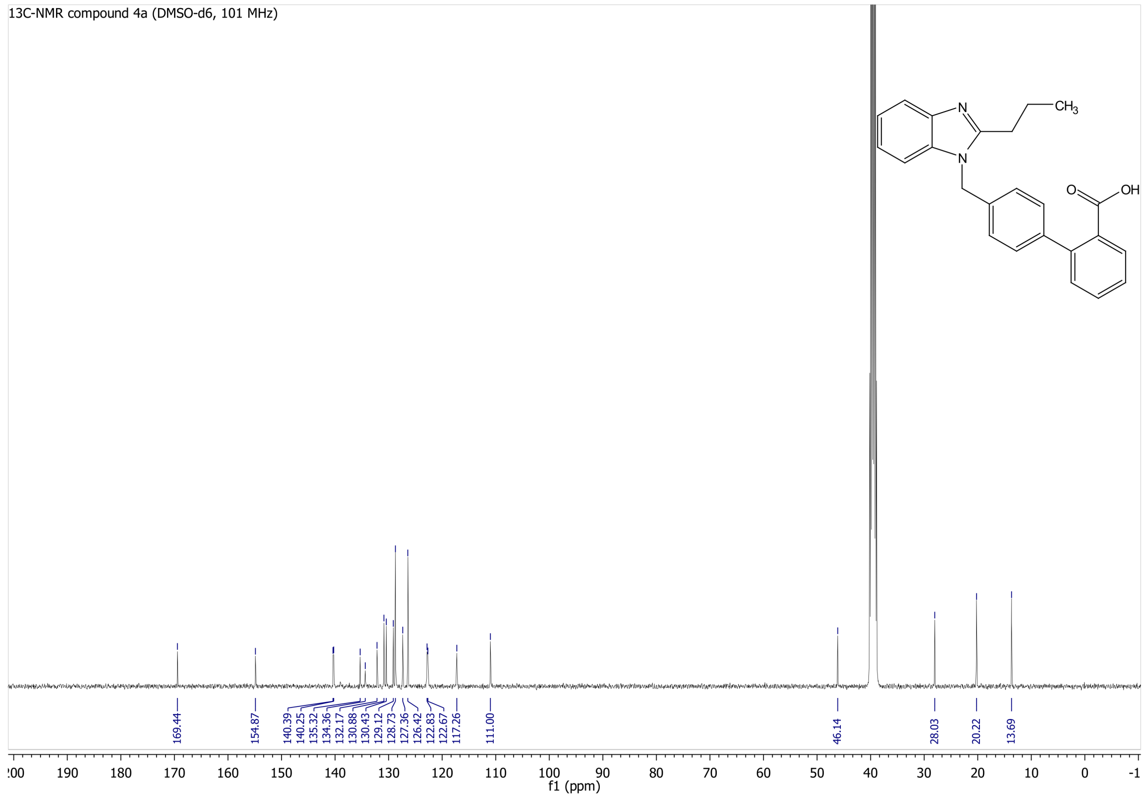

Figure S11: ^{1}H- and ^{13}C-NMR spectra of compound **4b**.

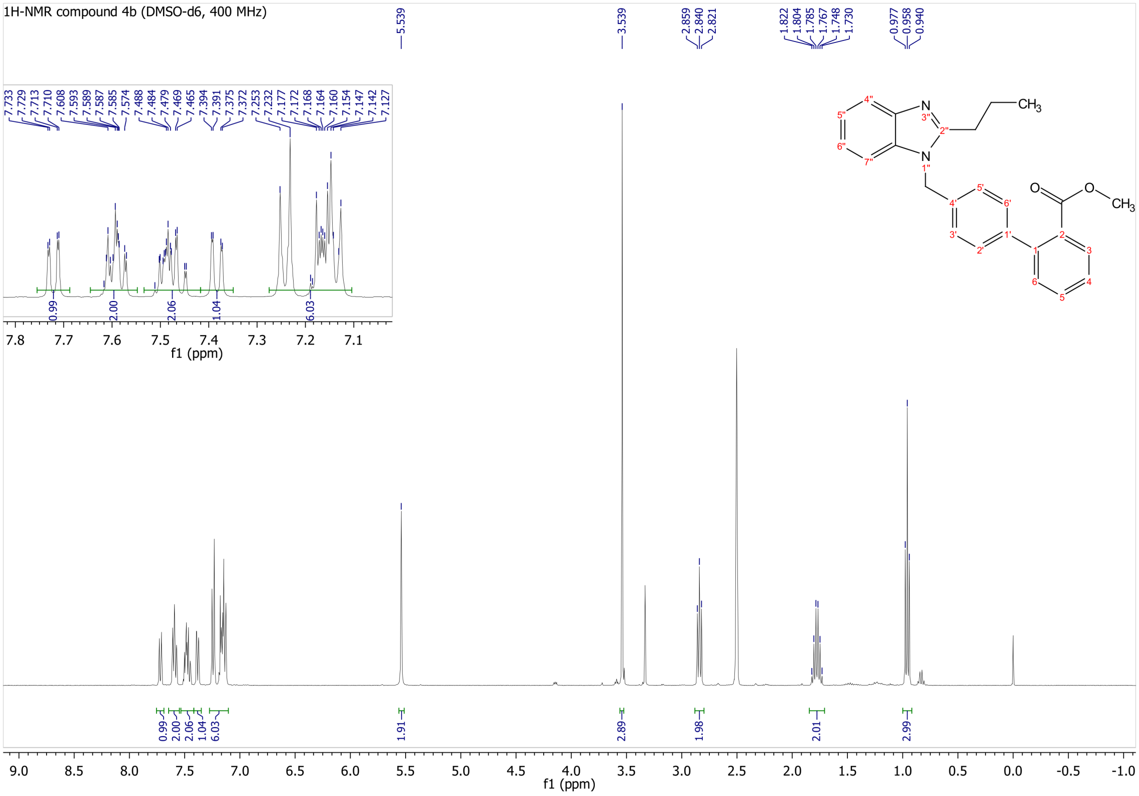

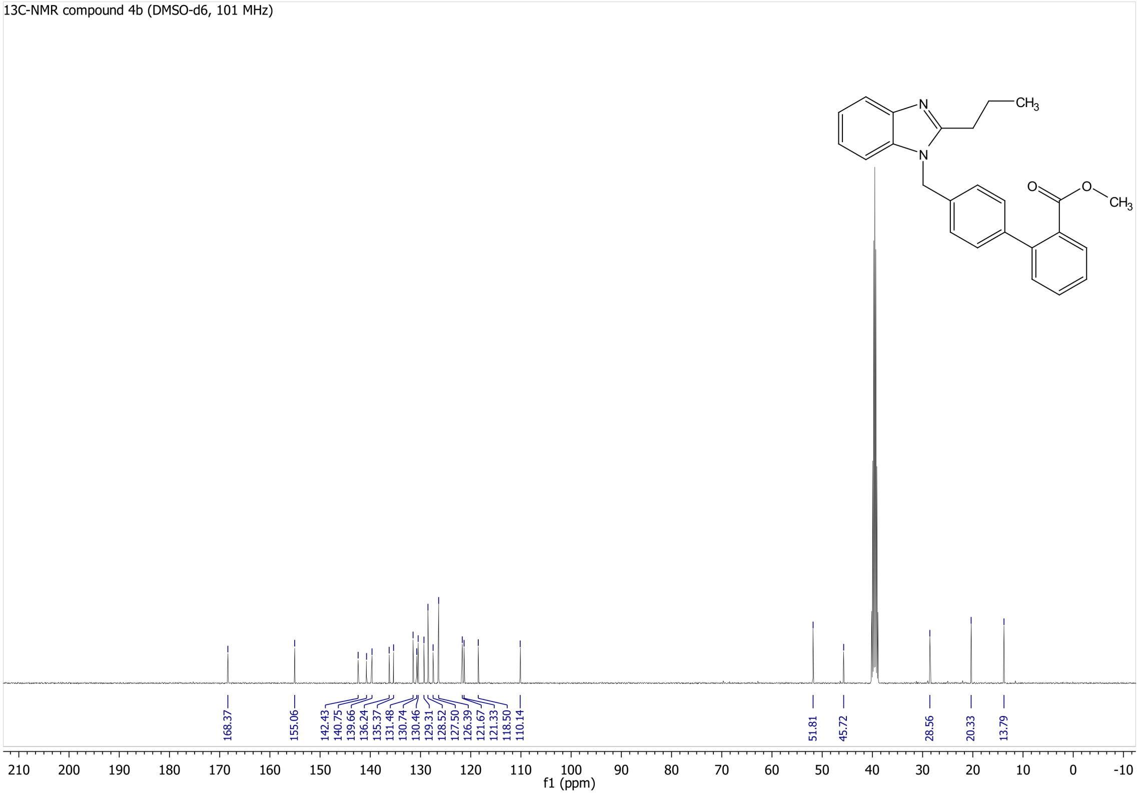

Figure S12: ^{1}H- and ^{13}C-NMR spectra of compound **4c**.

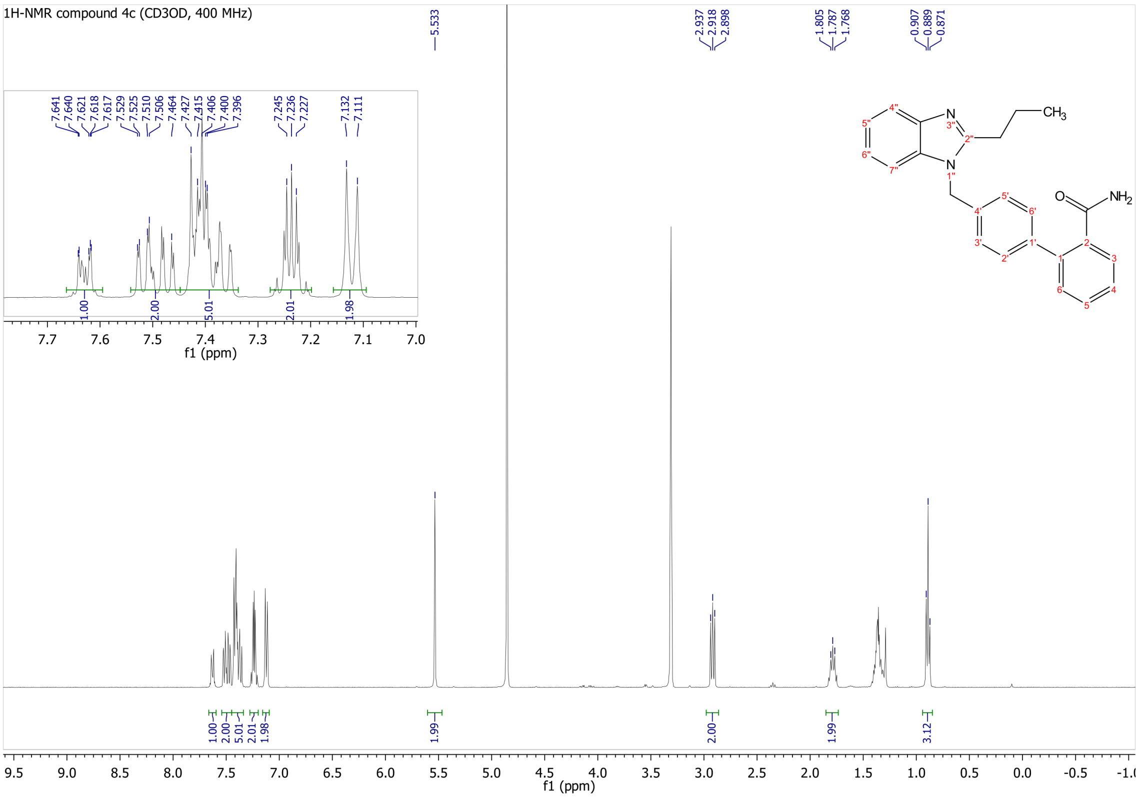

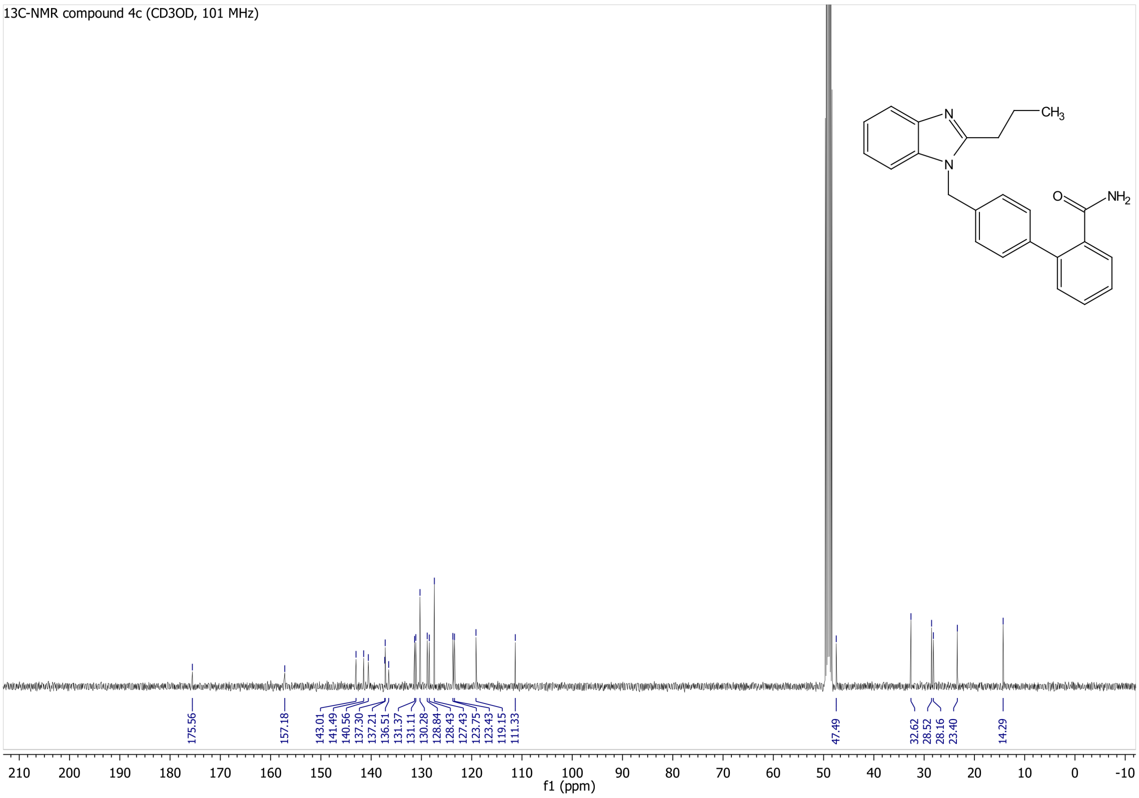

Figure S13: ^{1}H- and ^{13}C-NMR spectra of compound **5a**.

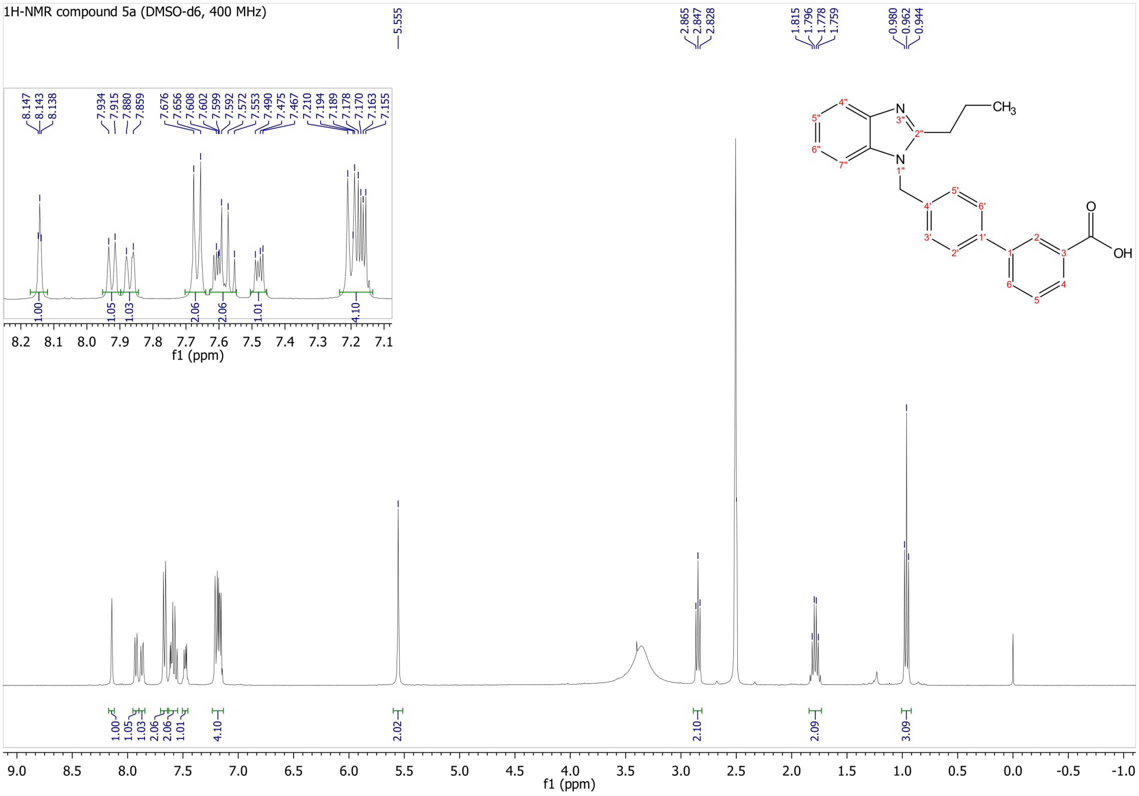

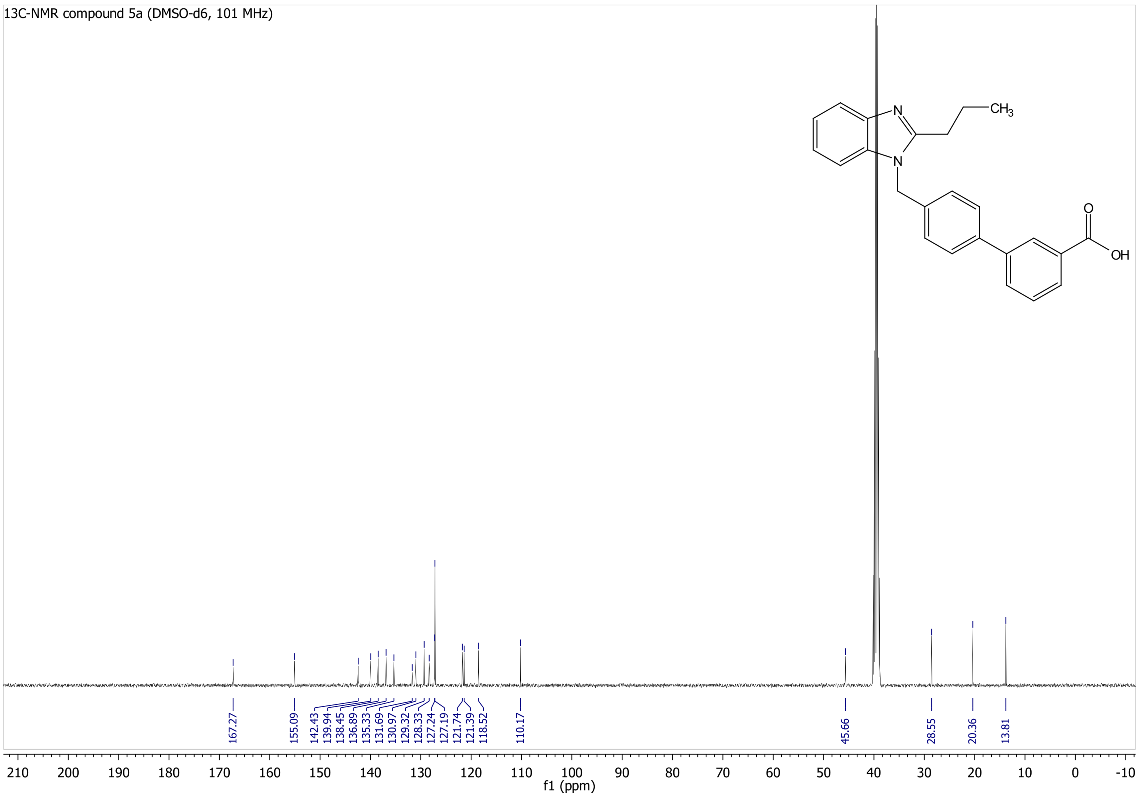

Figure S14: ^{1}H- and ^{13}C-NMR spectra of compound **5b**.

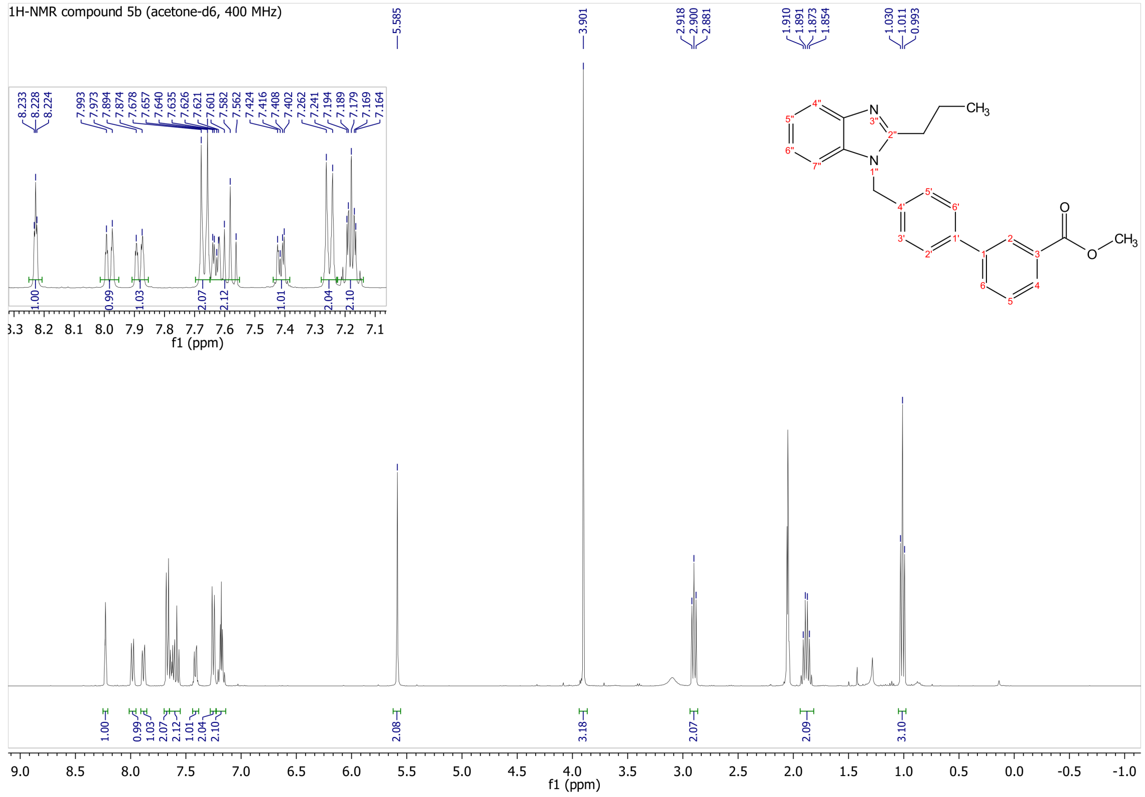

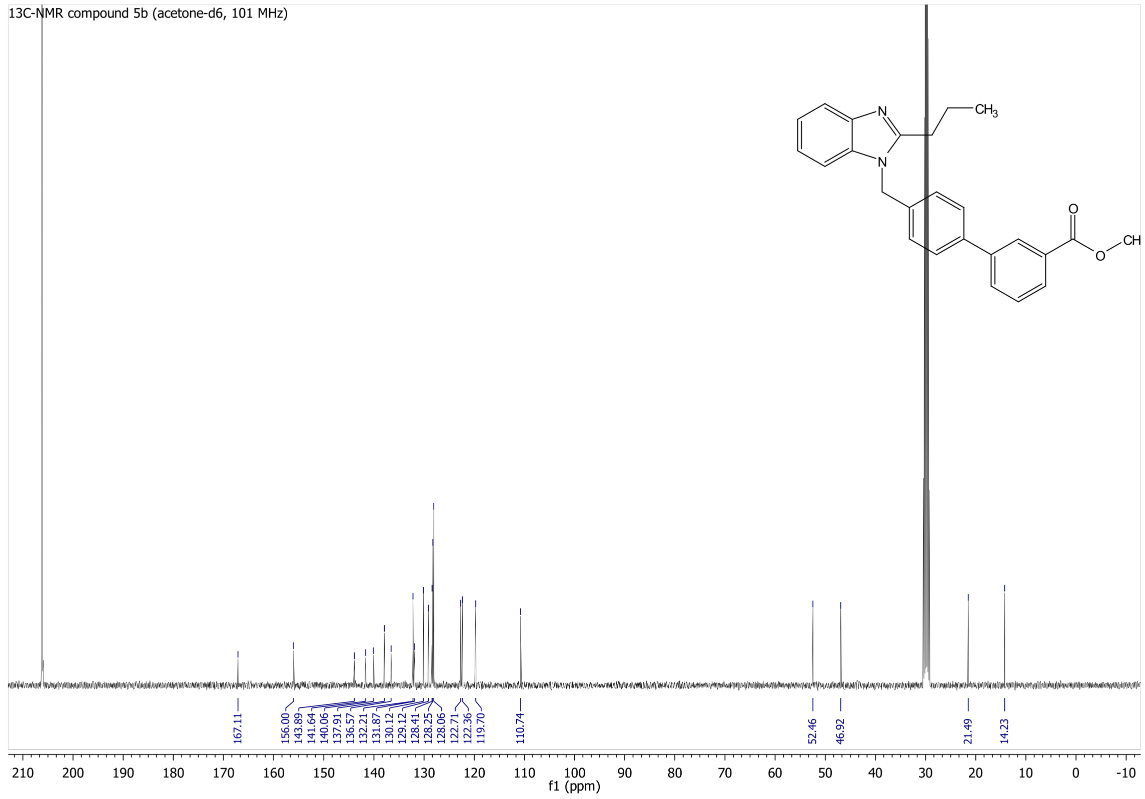

Figure S15: ^{1}H- and ^{13}C-NMR spectra of compound **5c**.

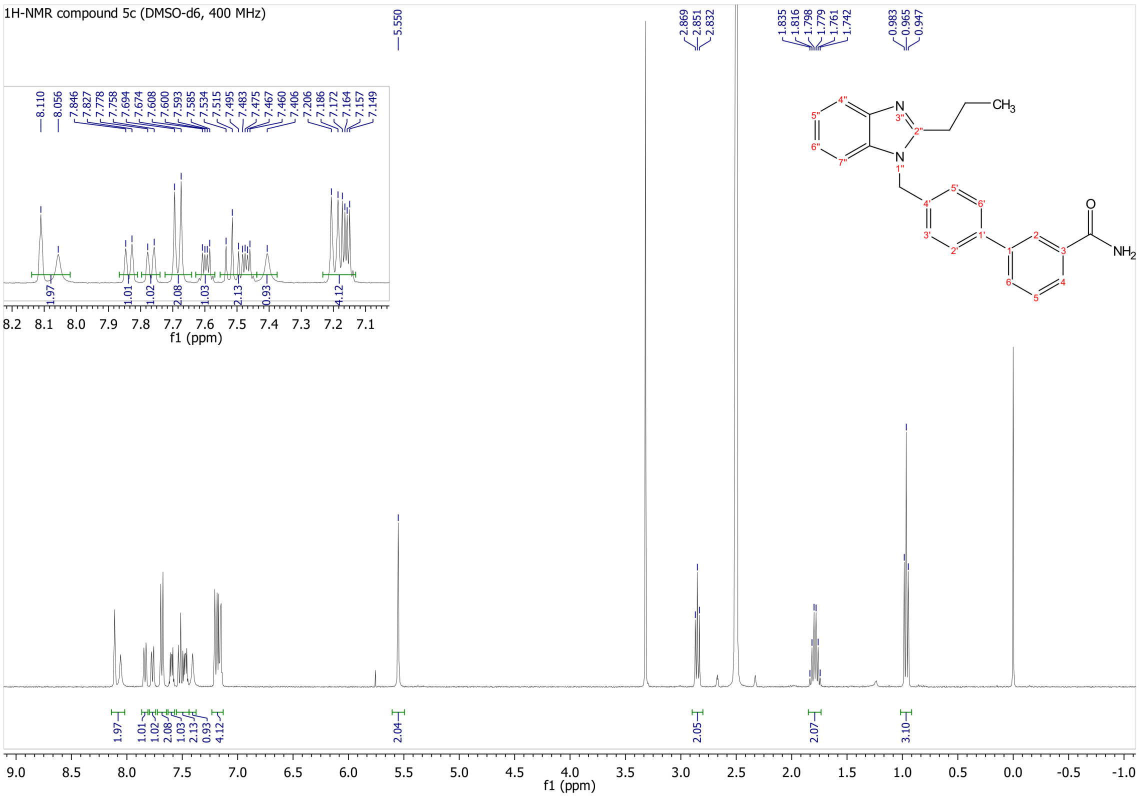

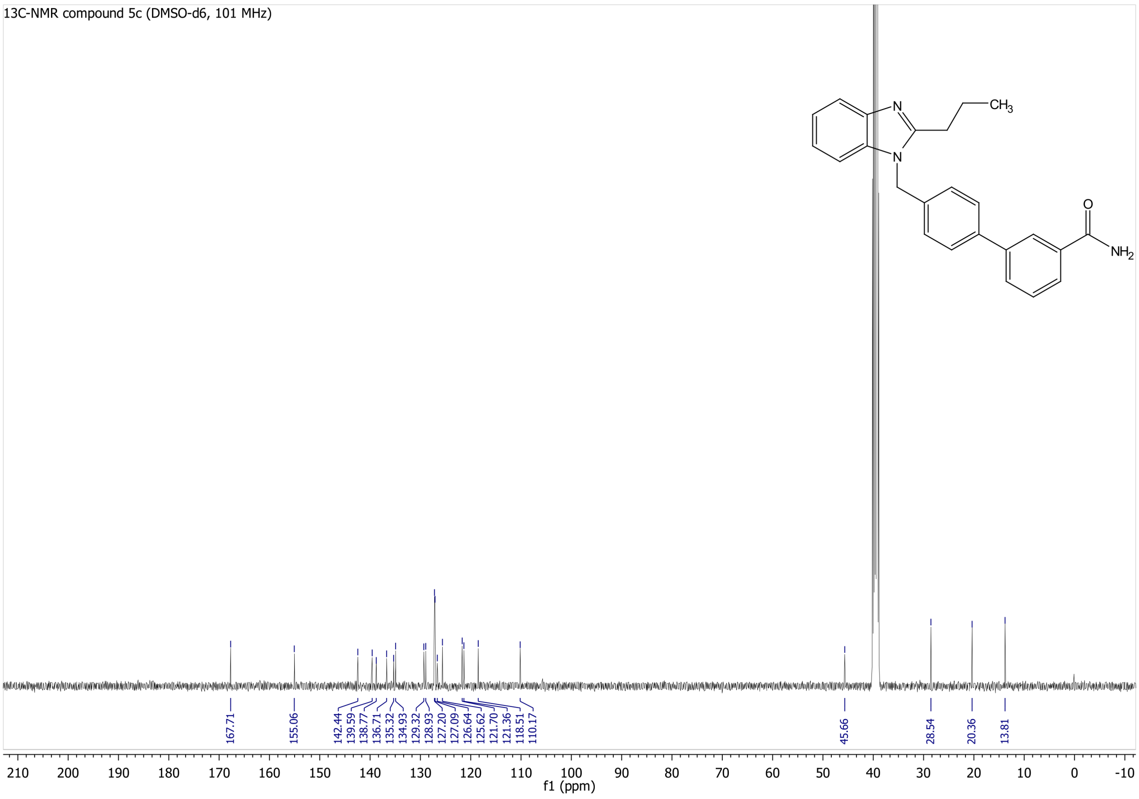

Figure S16: ^{1}H- and ^{13}C-NMR spectra of compound **6a**.

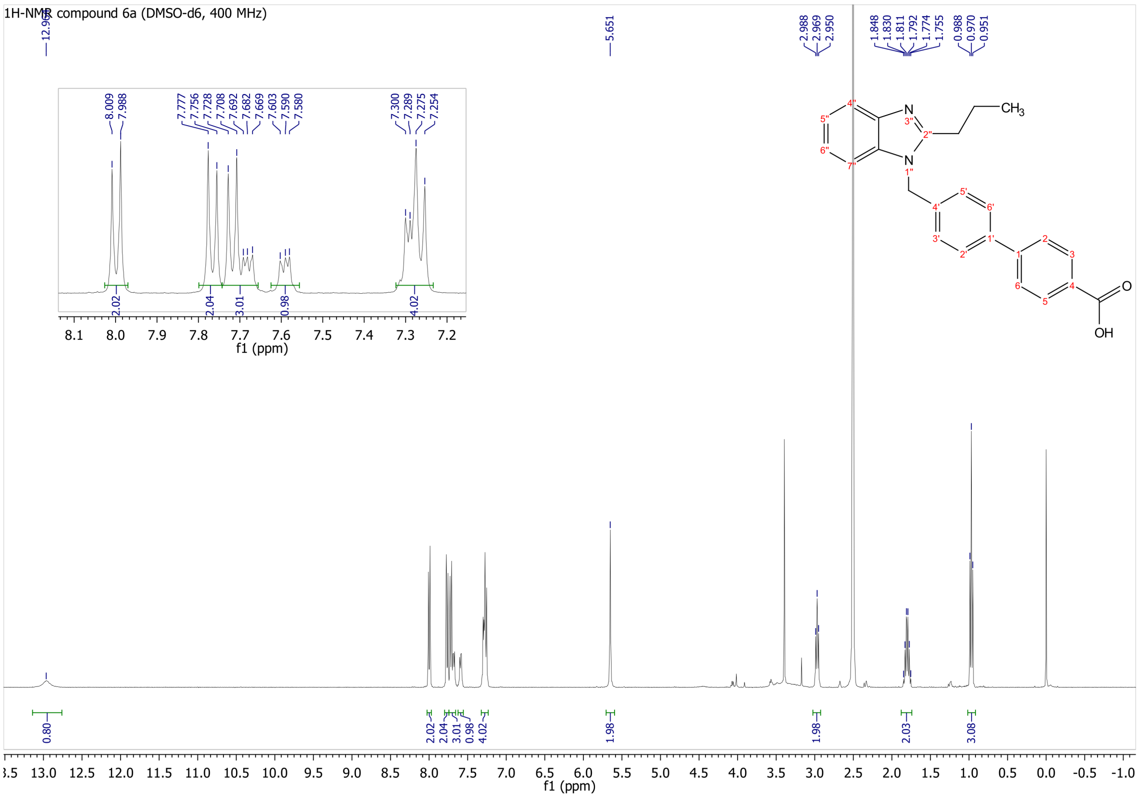

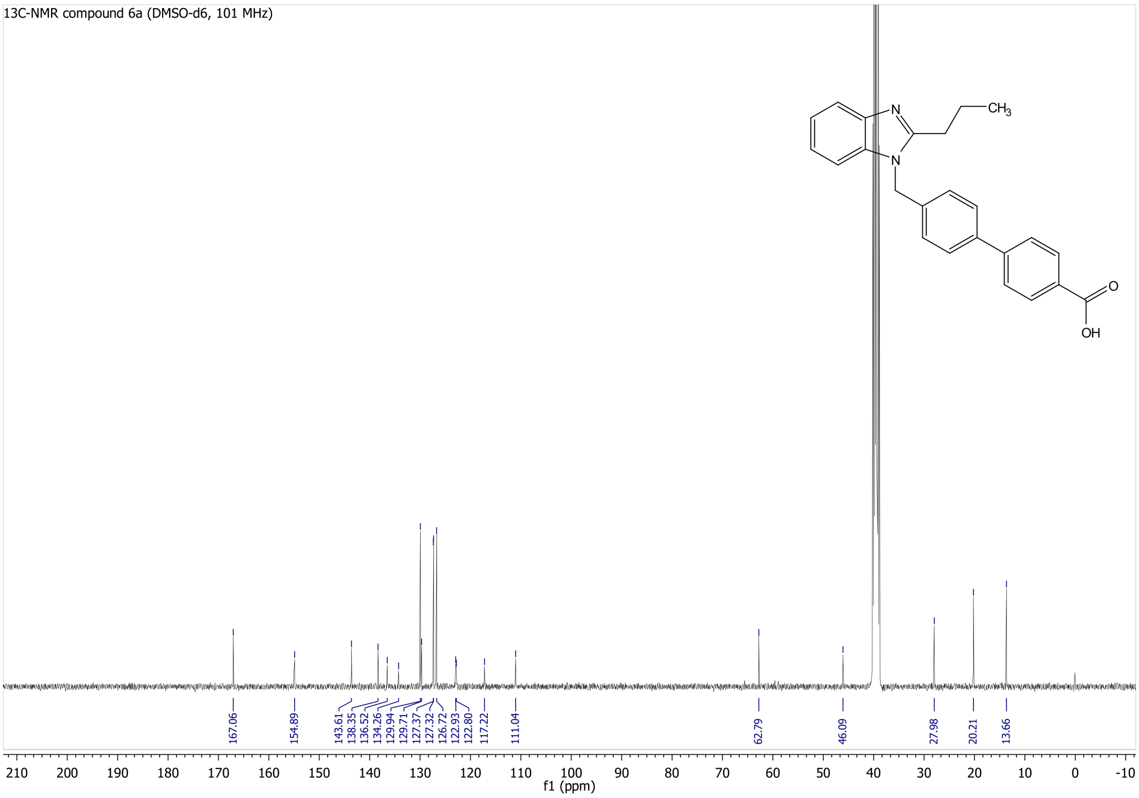

Figure S17: ^{1}H- and ^{13}C-NMR spectra of compound **6b**.

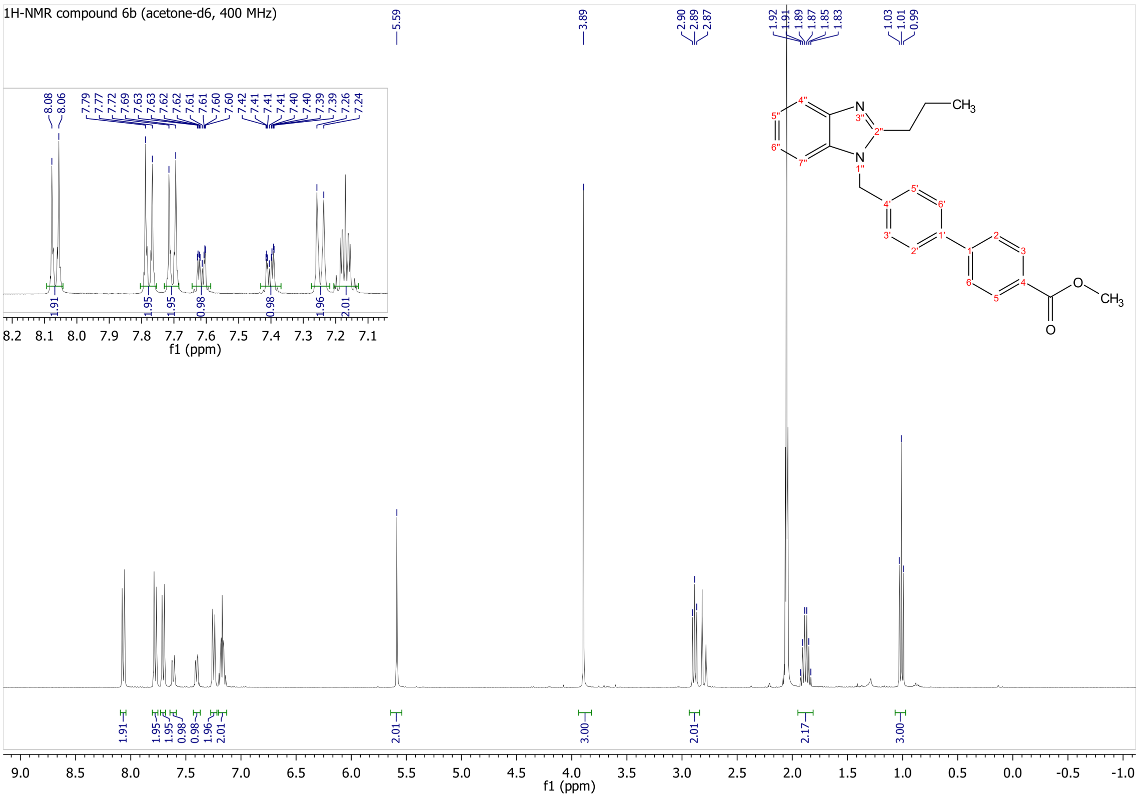

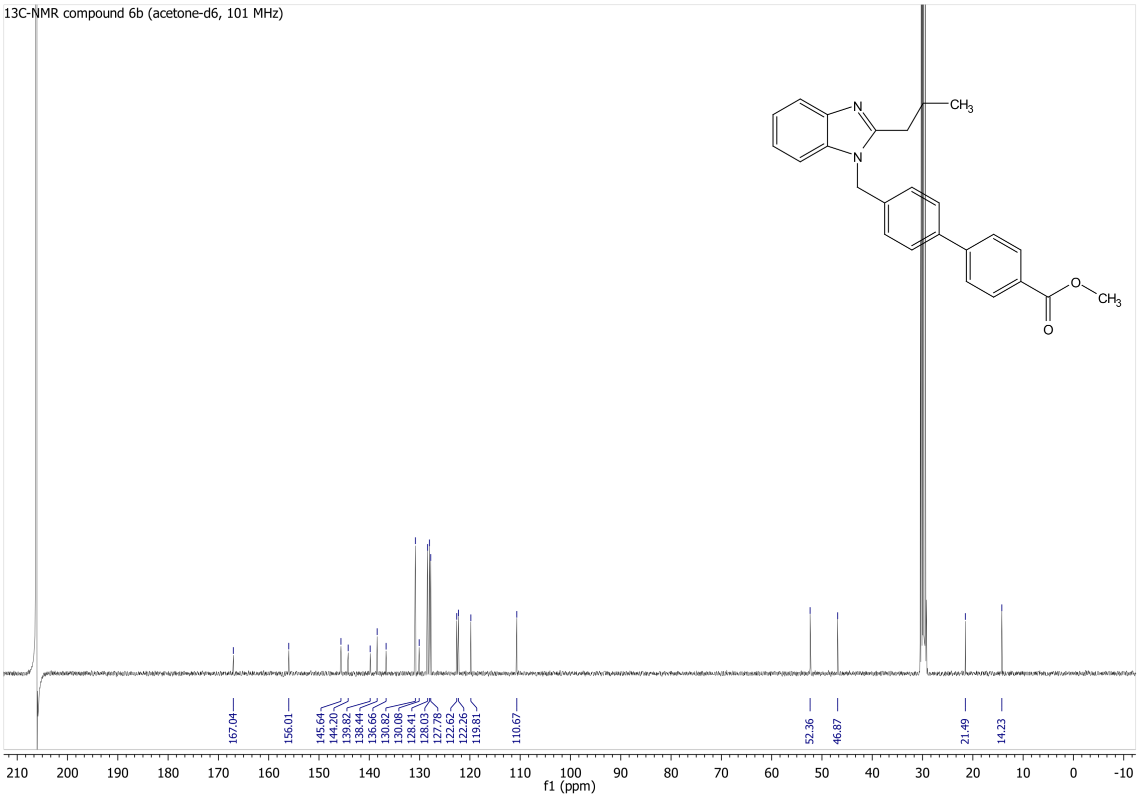

Figure S18: ^{1}H- and ^{13}C-NMR spectra of compound **6c**.

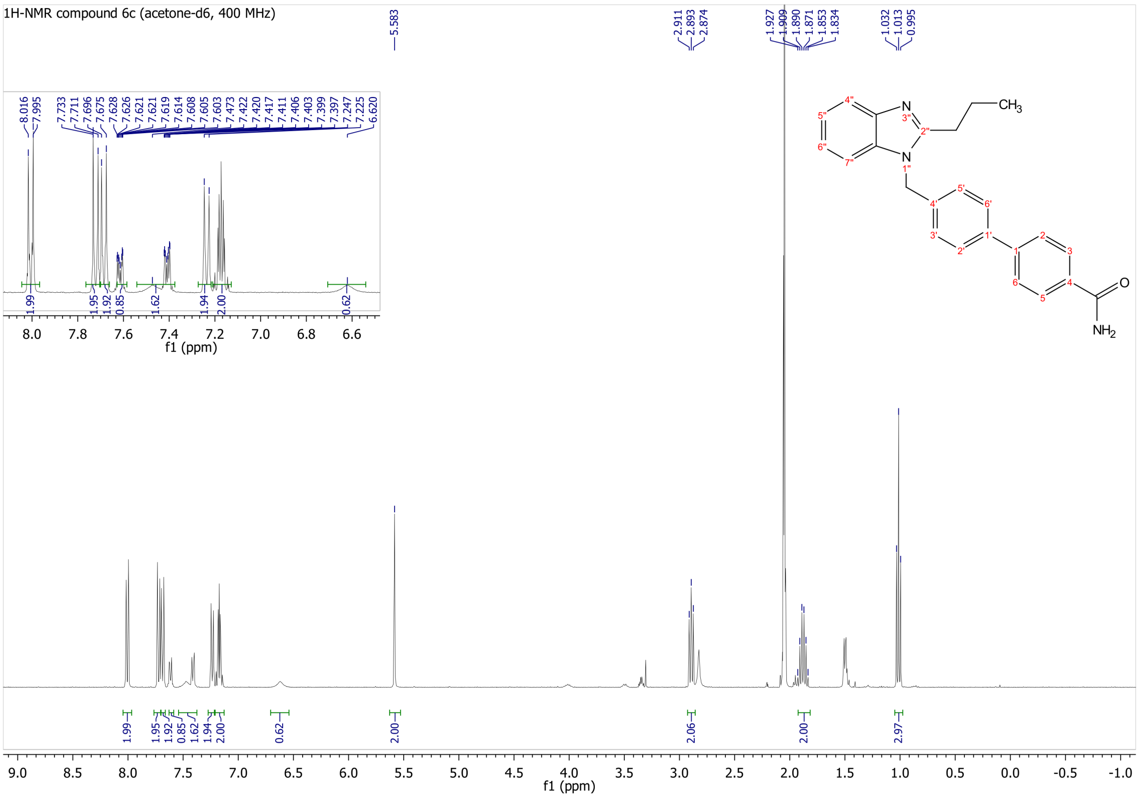

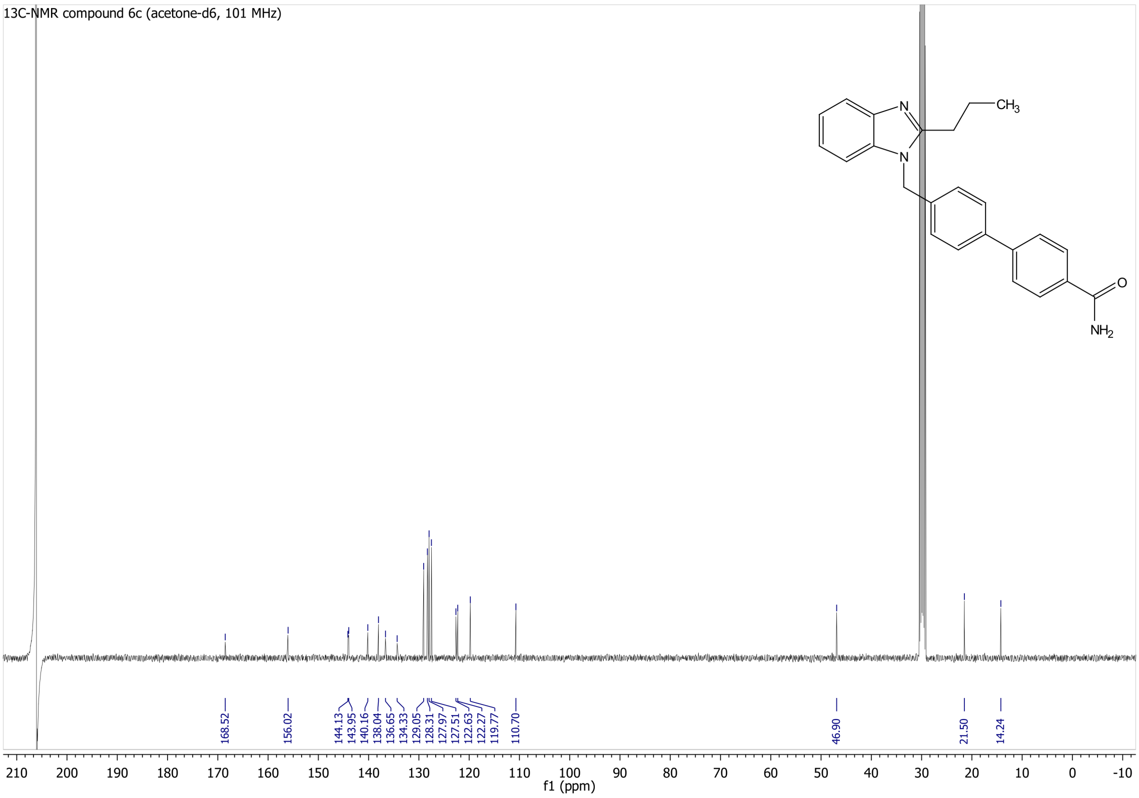

Figure S19: ^{1}H- and ^{13}C-NMR spectra of compound **7b**.

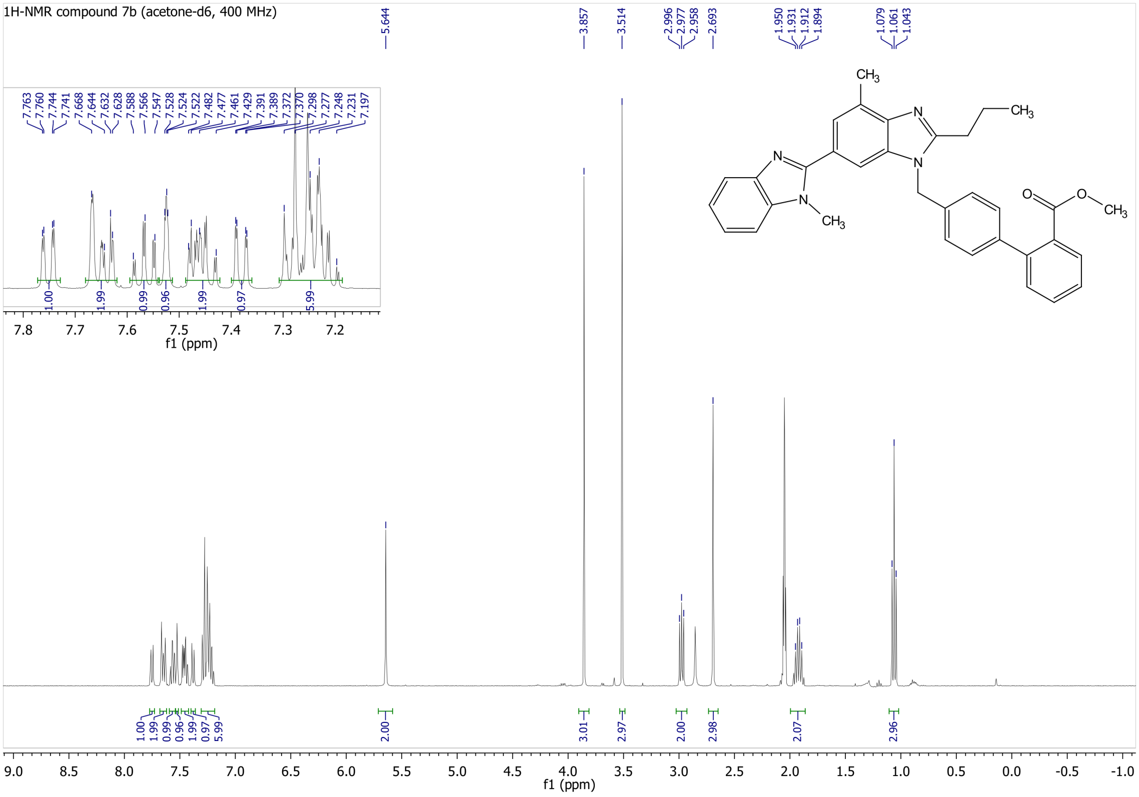

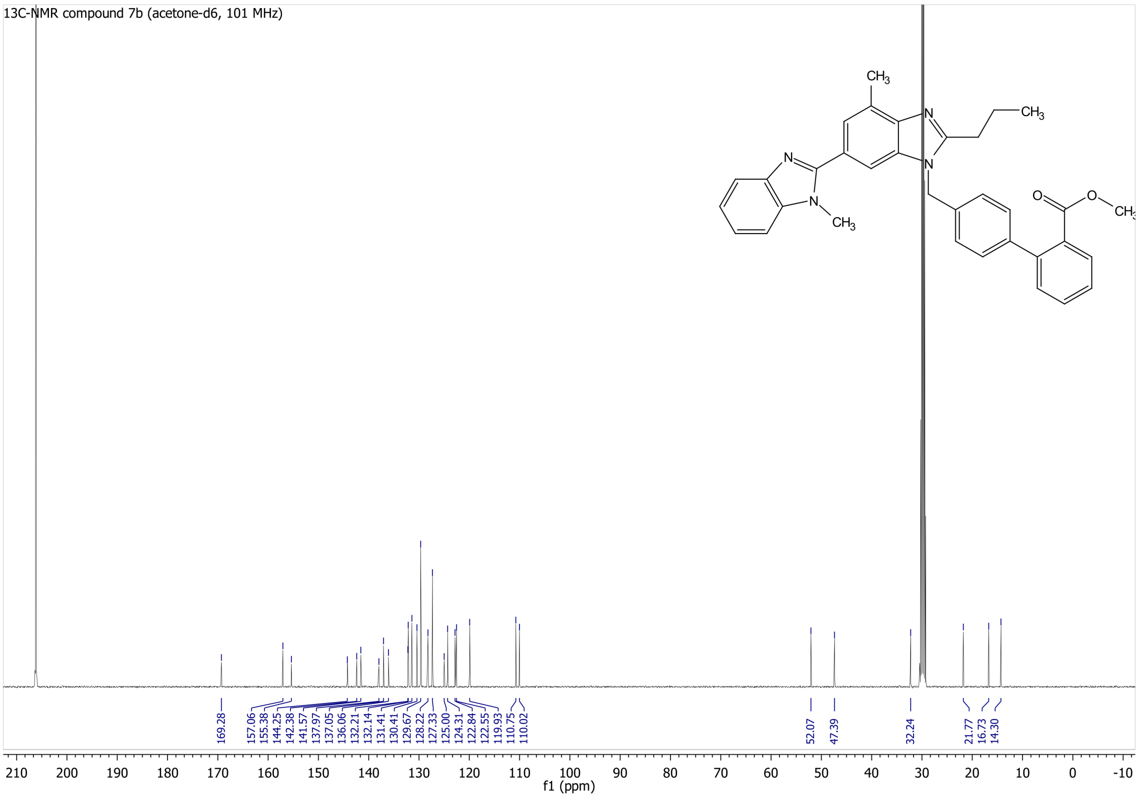

Figure S20: ^{1}H- and ^{13}C-NMR spectra of compound **7c**.

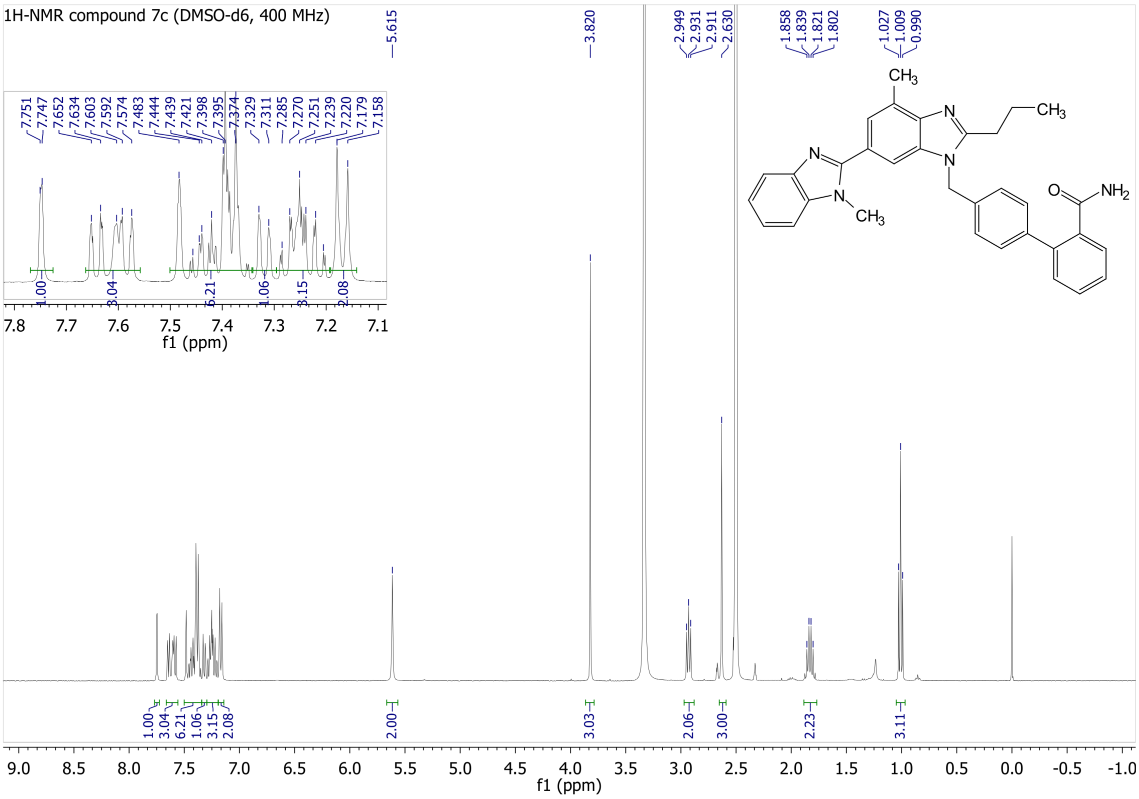

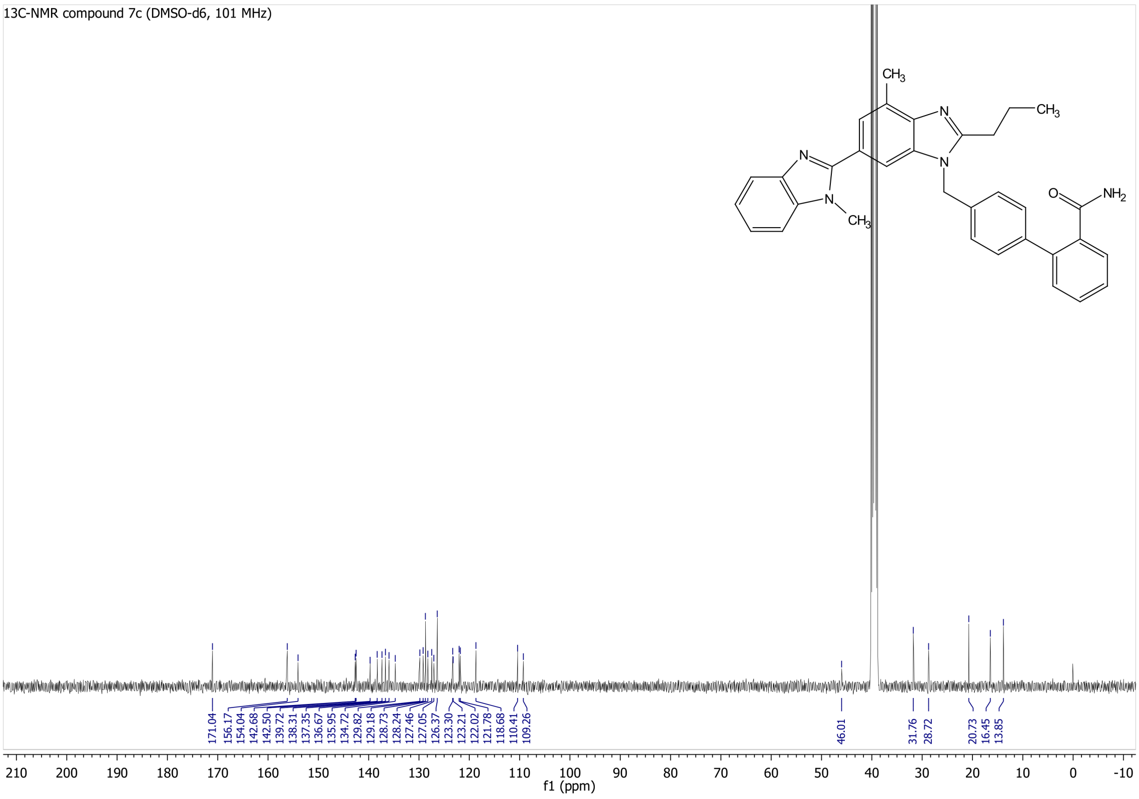

3. HPLC chromatograms

Figure S21: Chromatogram of compound **1a**.

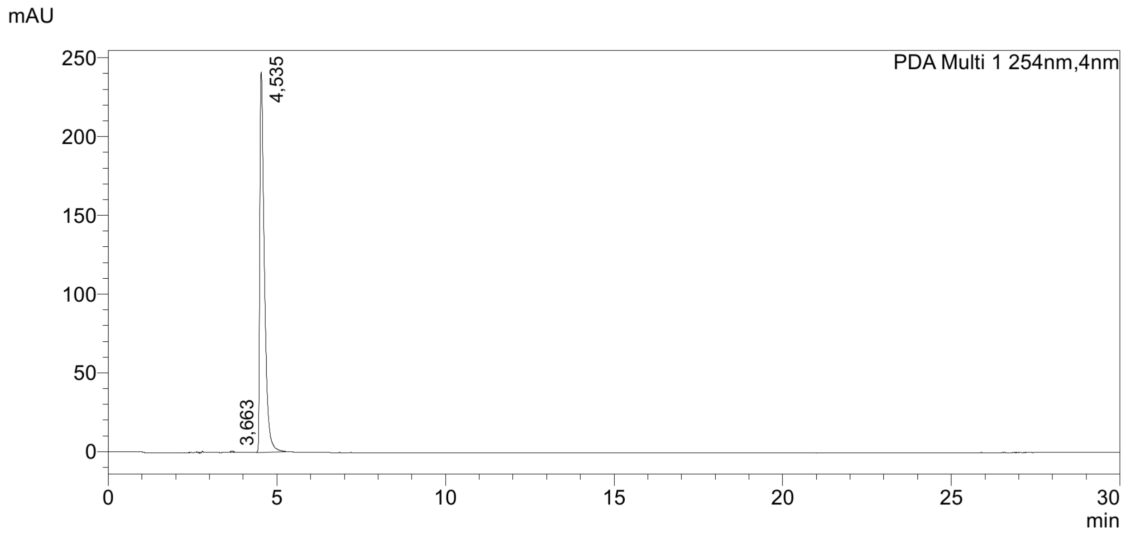

Compound 1a

PDA Ch1 254nm

Peak#	Ret. Time	Area	Height	Area%
1	3,663	6676	968	0,293
2	4,535	2268125	241392	99,707
Total		2274800	242360	100,000

Figure S22: Chromatogram of compound **1b**.

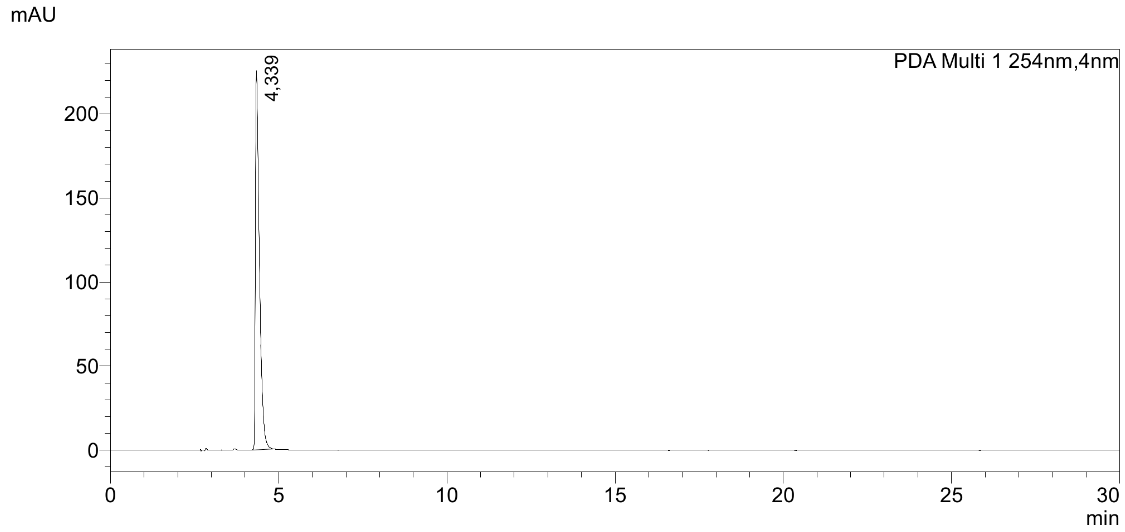

Compound 1b

PDA Ch1 254nm

Peak#	Ret. Time	Area	Height	Area%
1	4,339	1876865	224696	100,000
Total		1876865	224696	100,000

Figure S23: Chromatogram of compound **1c**.

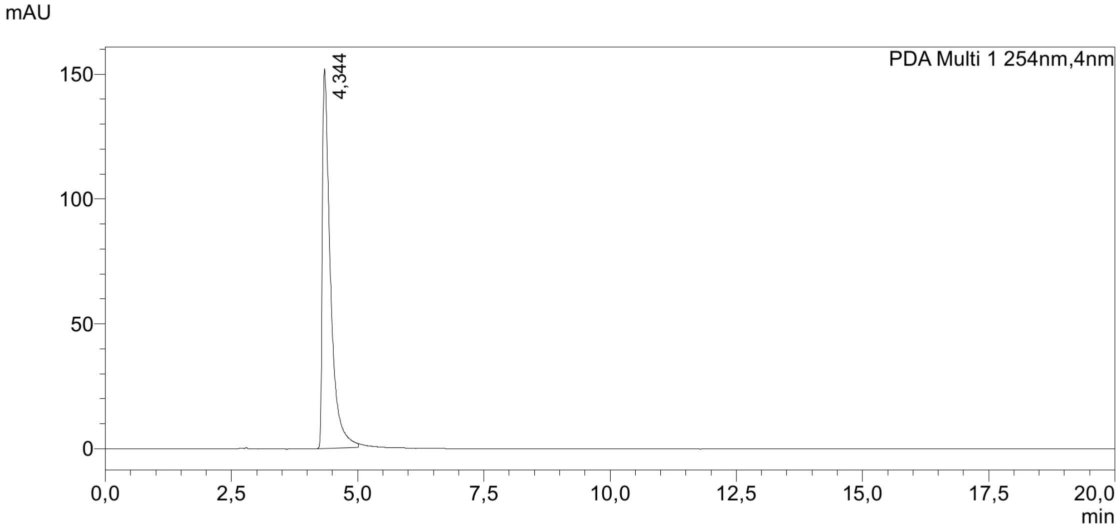

Compound 1c

PDA Ch1 254nm

Peak#	Ret. Time	Area	Height	Area%
1	4,344	1693987	152100	100,000
Total		1693987	152100	100,000

Figure S24: Chromatogram of compound **2a**.

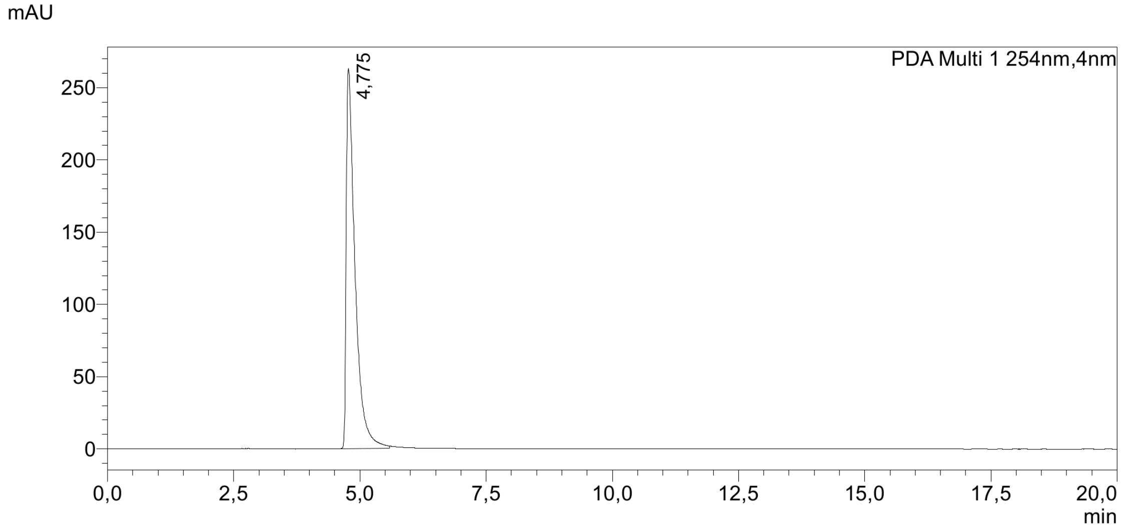

Compound 2a

PDA Ch1 254nm

Peak#	Ret. Time	Area	Height	Area%
1	4,775	3178665	262914	100,000
Total		3178665	262914	100,000

Figure S25: Chromatogram of compound **2b**.

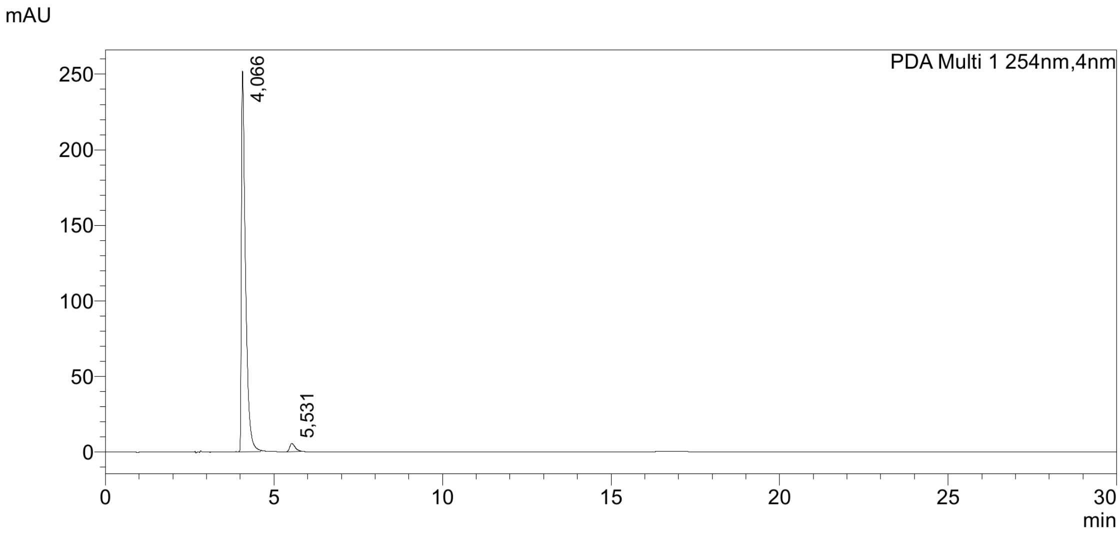

Compound 2b

PDA Ch1 254nm

Peak#	Ret. Time	Area	Height	Area%
1	4,066	2117173	250478	97,236
2	5,531	60177	5411	2,764
Total		2177350	255889	100,000

Figure S26: Chromatogram of compound **2c**.

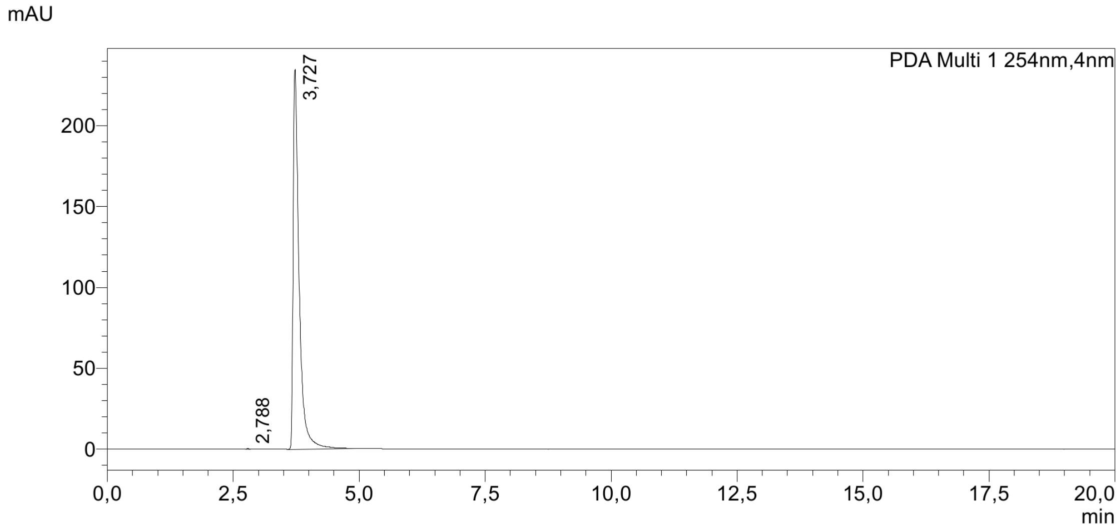

Compound 2c

PDA Ch1 254nm

Peak#	Ret. Time	Area	Height	Conc.	Unit	Mark	Name
1	2,788	1009	331	0,052			
2	3,727	1929574	234529	99,948		S	
Total		1930583	234860				

Figure S27: Chromatogram of compound **3a**.

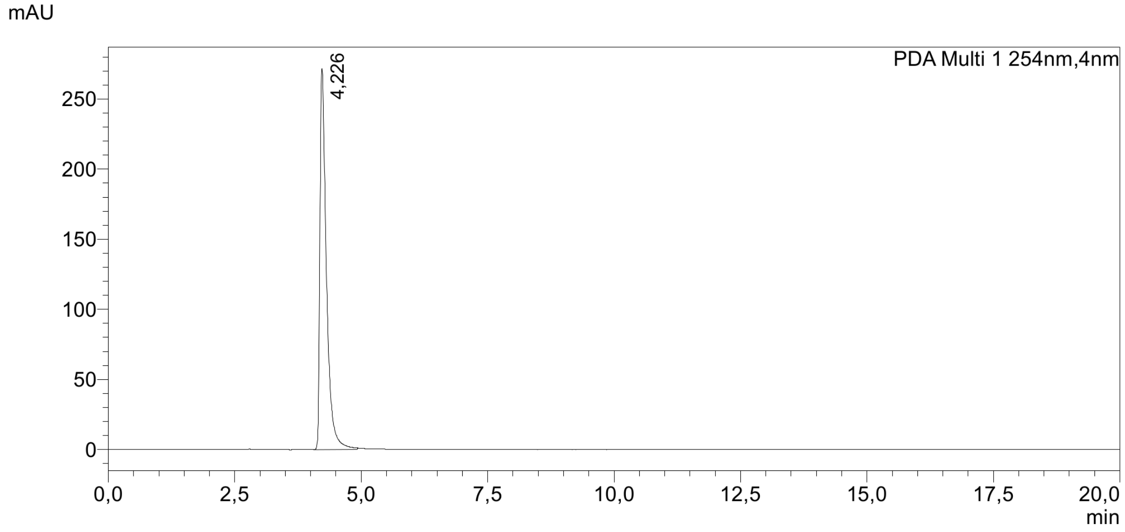

Compound 3a

PDA Ch1 254nm

Peak#	Ret. Time	Area	Height	Area%
1	4,226	2439859	270861	100,000
Total		2439859	270861	100,000

Figure S28: Chromatogram of compound **3b**.

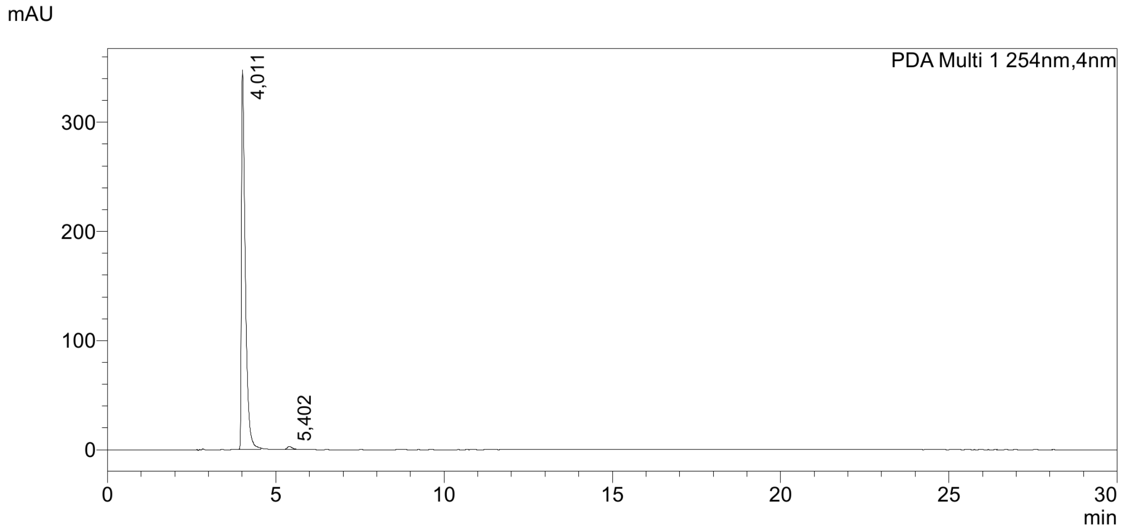

Compound 3b

PDA Ch1 254nm

Peak#	Ret. Time	Area	Height	Area%
1	4,011	2777507	346405	98,945
2	5,402	29616	2770	1,055
Total		2807123	349175	100,000

Figure S29: Chromatogram of compound **3c**.

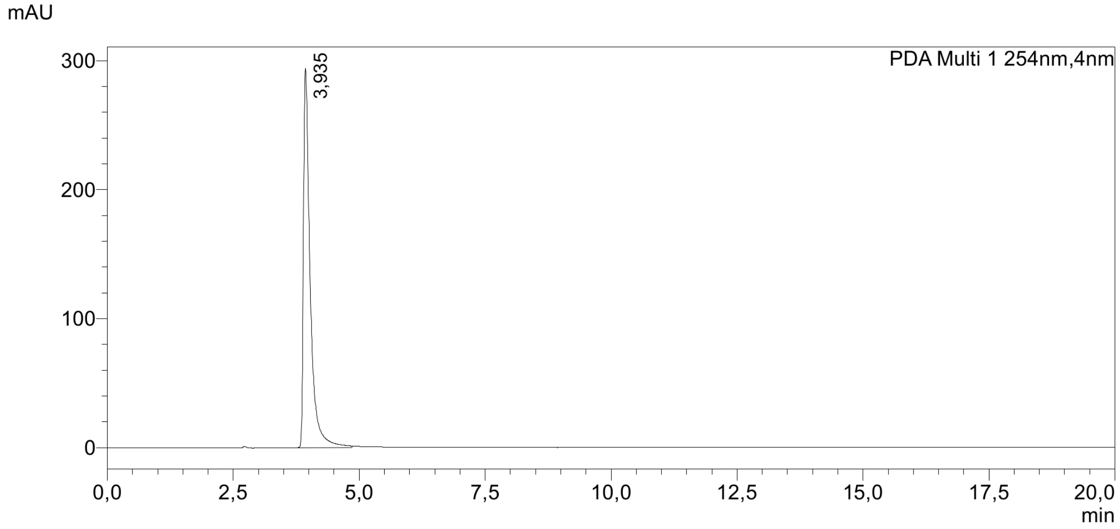

Compound 3c

PDA Ch1 254nm

Peak#	Ret. Time	Area	Height	Area%
1	3,935	2753195	293770	100,000
Total		2753195	293770	100,000

Figure S30: Chromatogram of compound **4a**.

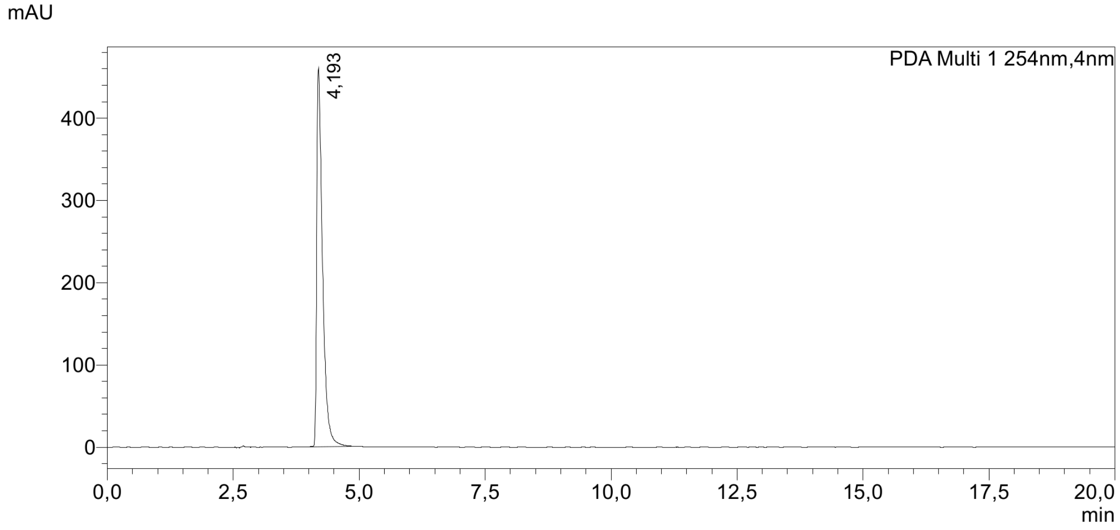

Compound 4a

PDA Ch1 254nm

Peak#	Ret. Time	Area	Height	Area%
1	4,193	3699173	458636	100,000
Total		3699173	458636	100,000

Figure S31: Chromatogram of compound **4b**.

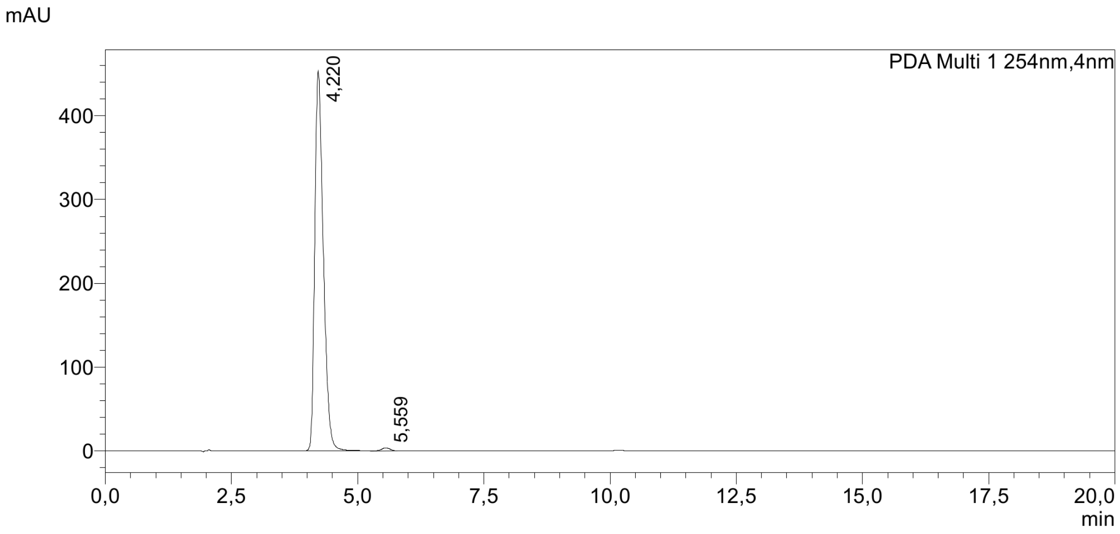

Compound 4b

PDA Ch1 254nm

Peak#	Ret. Time	Area	Height	Area%
1	4,220	5363128	452555	99,219
2	5,559	42189	3553	0,781
Total		5405317	456108	100,000

Figure S32: Chromatogram of compound **4c**.

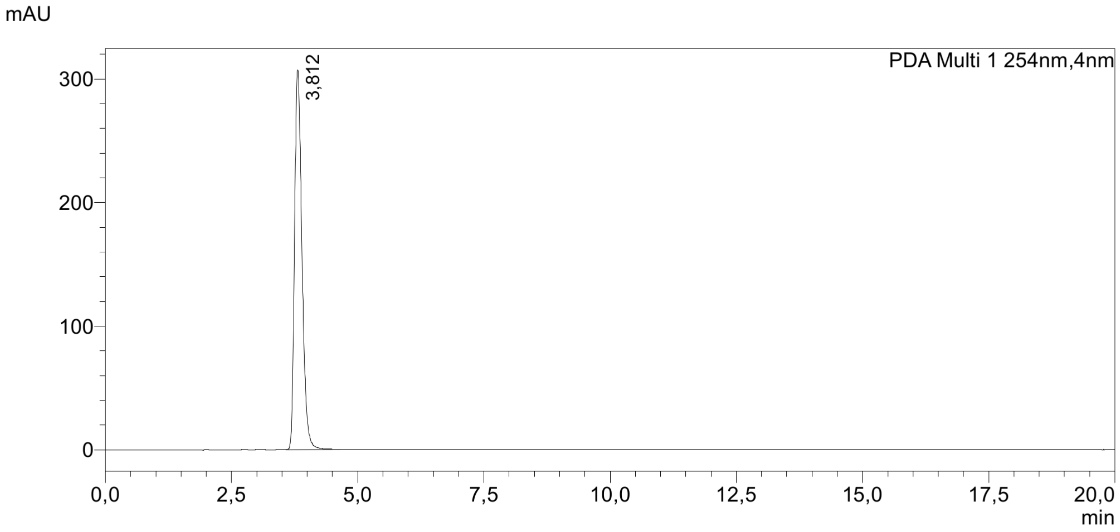

Compound 4c

PDA Ch1 254nm

Peak#	Ret. Time	Area	Height	Area%
1	3,812	3135290	306984	100,000
Total		3135290	306984	100,000

Figure S33: Chromatogram of compound **5a**.

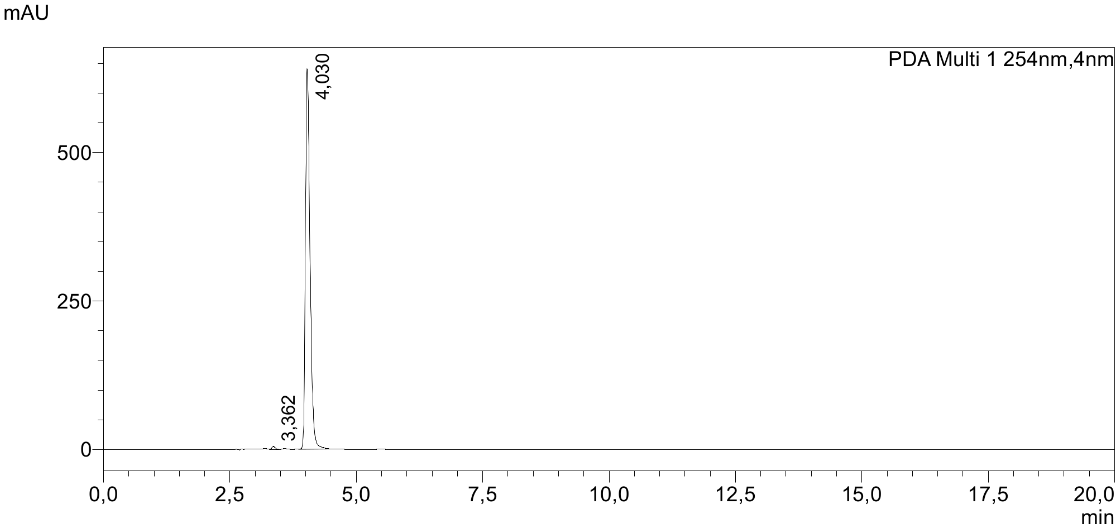

Compound 5a

PDA Ch1 254nm

Peak#	Ret. Time	Area	Height	Area%
1	3,362	21767	4800	0,535
2	4,030	4044598	639969	99,465
Total		4066366	644769	100,000

Figure S34: Chromatogram of compound **5b**.

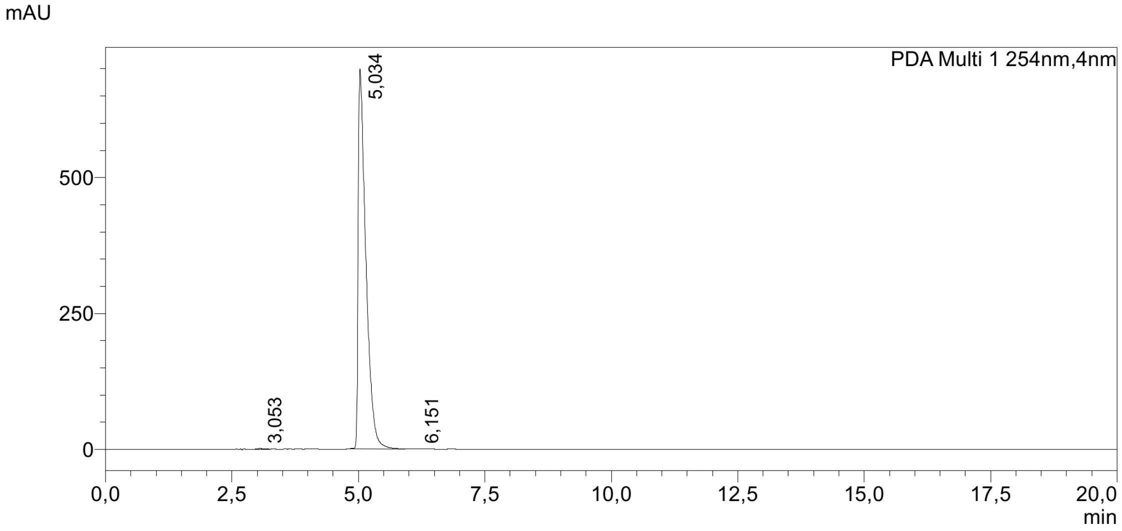

Compound 5b

PDA Ch1 254nm

Peak#	Ret. Time	Area	Height	Area%
1	3,053	9382	1380	0,126
2	5,034	7399098	696698	99,760
3	6,151	8386	563	0,113
Total		7416866	698641	100,000

Figure S35: Chromatogram of compound **5c**.

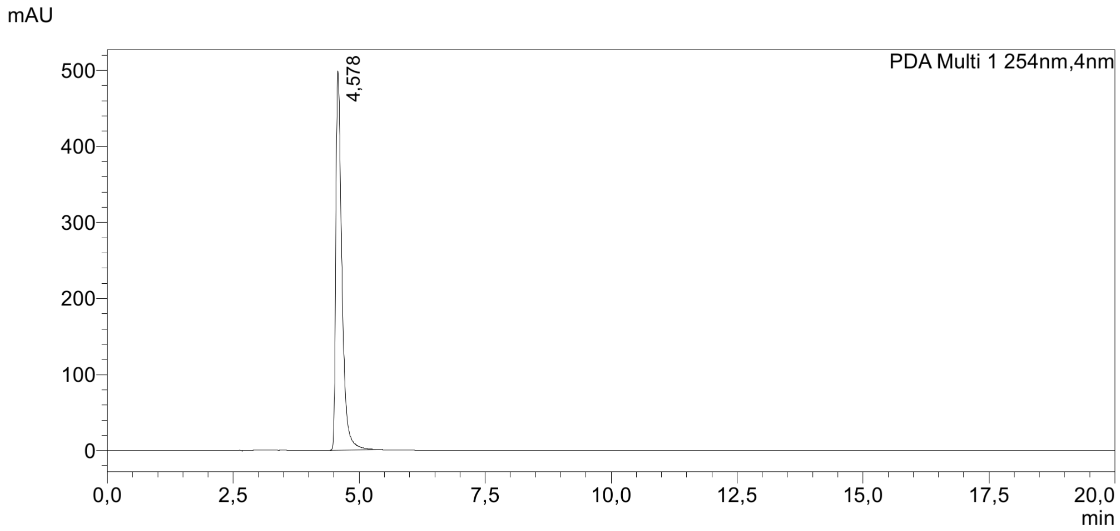

Compound 5c

PDA Ch1 254nm

Peak#	Ret. Time	Area	Height	Area%
1	4,578	4174484	496800	100,000
Total		4174484	496800	100,000

Figure S36: Chromatogram of compound **6a**.

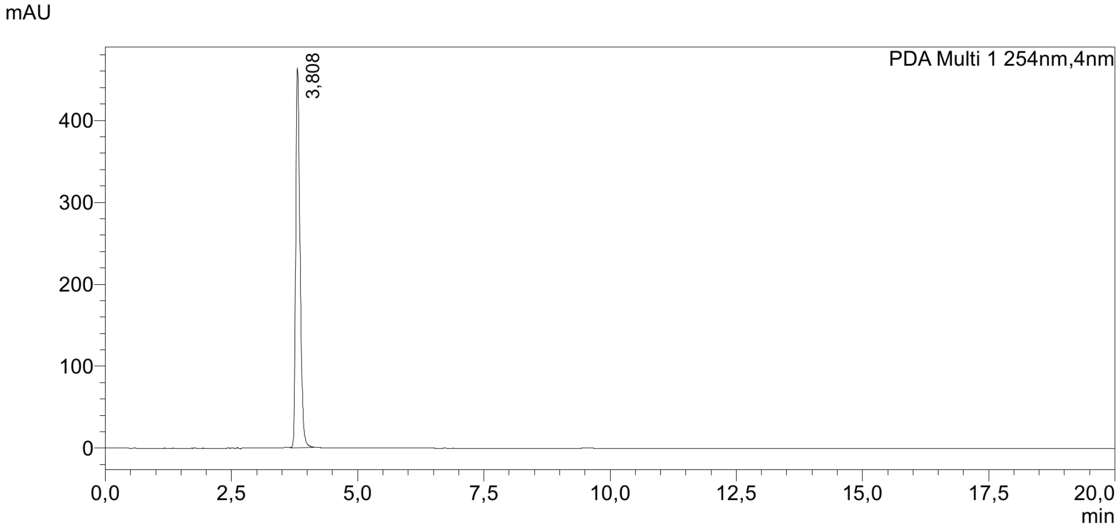

Compound 6a

PDA Ch1 254nm

Peak#	Ret. Time	Area	Height	Area%
1	3,808	2748758	461827	100,000
Total		2748758	461827	100,000

Figure S37: Chromatogram of compound **6b**.

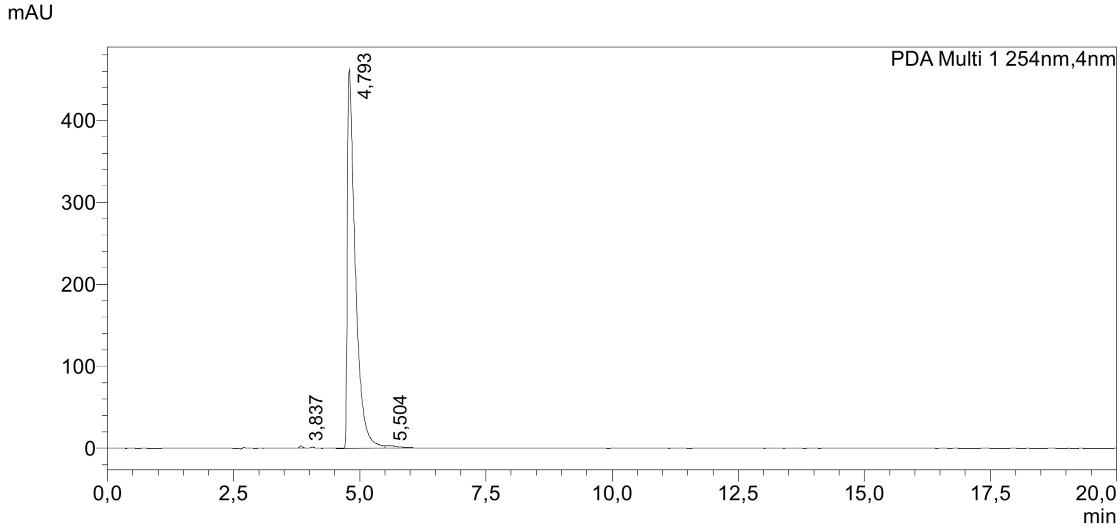

Compound 6b

PDA Ch1 254nm

Peak#	Ret. Time	Area	Height	Area%
1	3,837	9866	2465	0,193
2	4,793	5070561	462661	98,981
3	5,504	42353	2670	0,827
Total		5122779	467796	100,000

Figure S38: Chromatogram of compound **6c**.

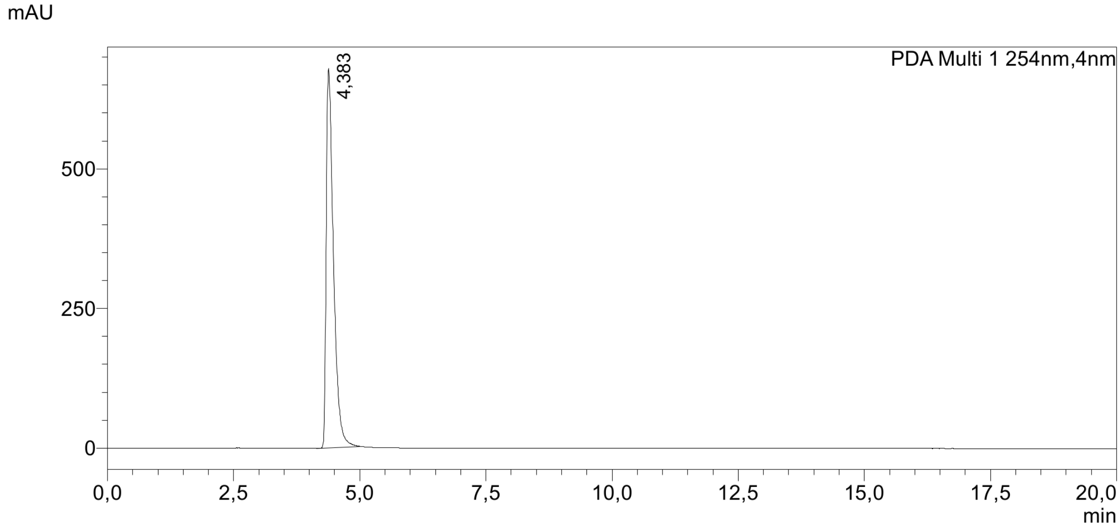

Compound 6c

PDA Ch1 254nm

Peak#	Ret. Time	Area	Height	Area%
1	4,383	6815091	678994	100,000
Total		6815091	678994	100,000

Figure S39: Chromatogram of compound **7b**.

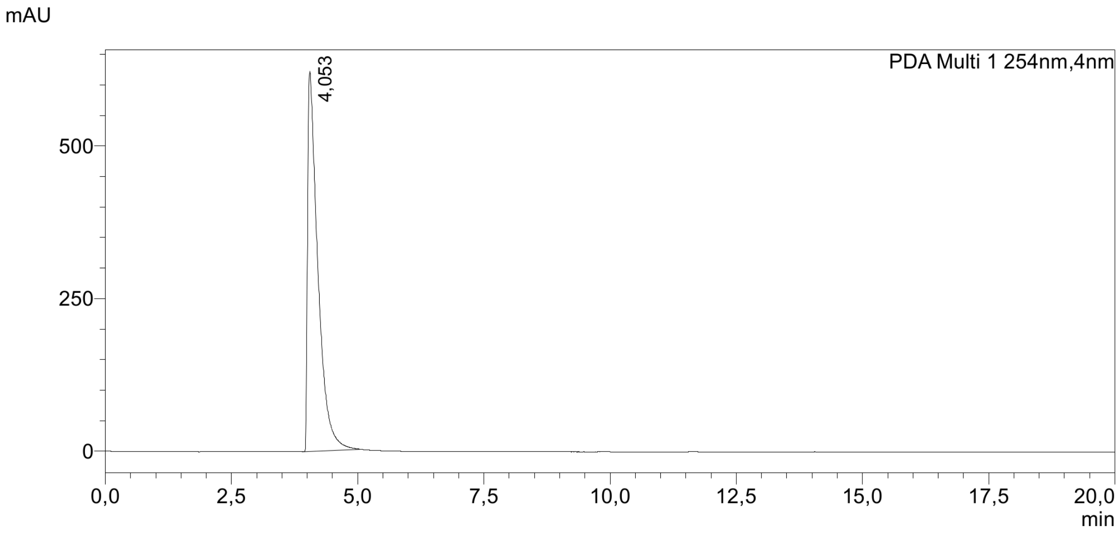

Compound 7b

PDA Ch1 254nm

Peak#	Ret. Time	Area	Height	Area%
1	4,053	8799854	621907	100,000
Total		8799854	621907	100,000

Figure S40: Chromatogram of compound **7c**.

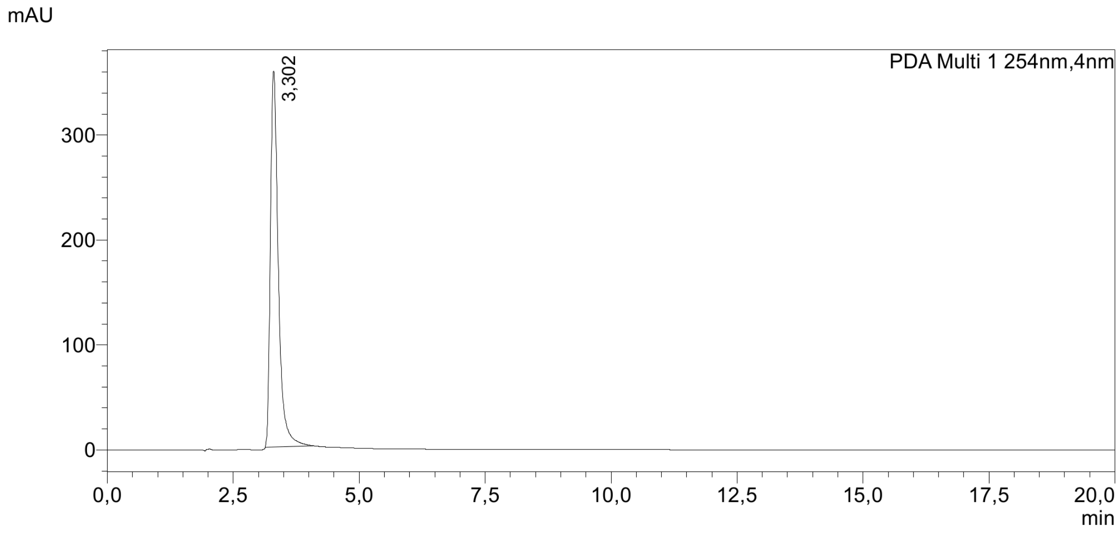

Compound 7c

PDA Ch1 254nm

Peak#	Ret. Time	Area	Height	Area%
1	3,302	3906253	358028	100,000
Total		3906253	358028	100,000

4. HRMS spectra

Figure S41: HRMS spectrum of compound **1a**.

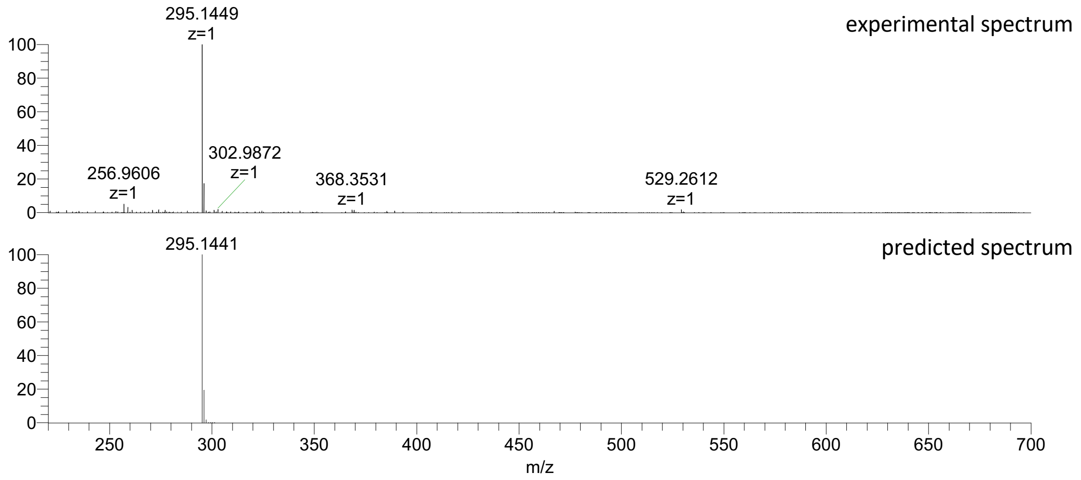

Figure S42: HRMS spectrum of compound **1b**.

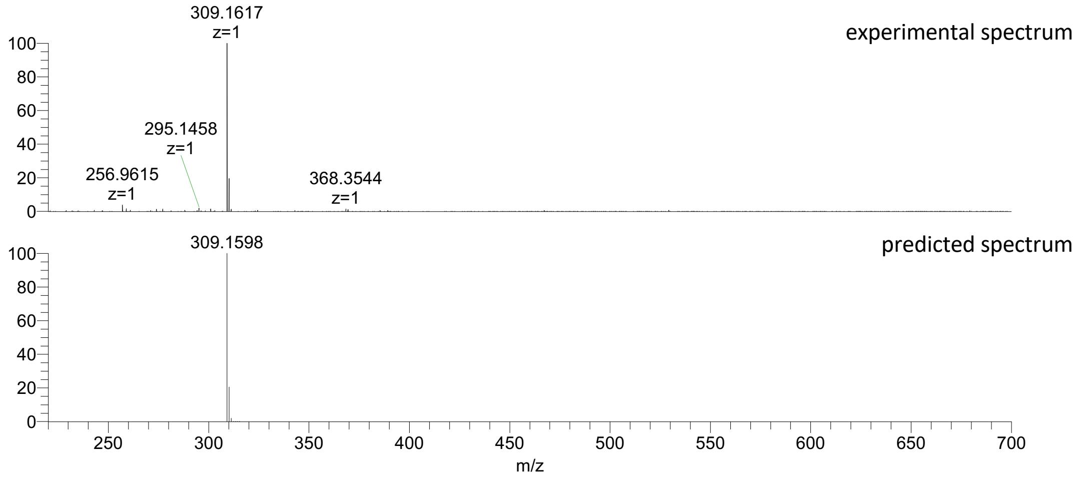

Figure S43: HRMS spectrum of compound **1c**.

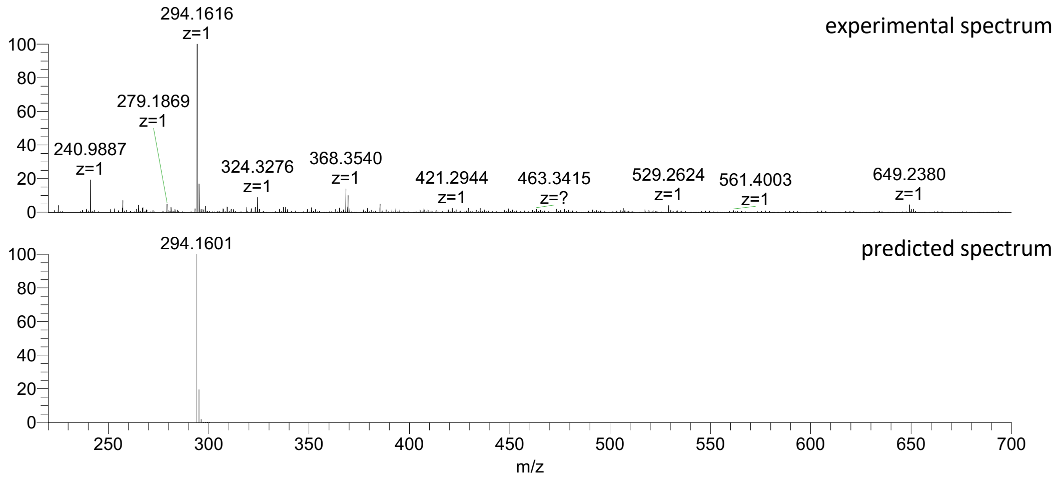

Figure S44: HRMS spectrum of compound **2a**.

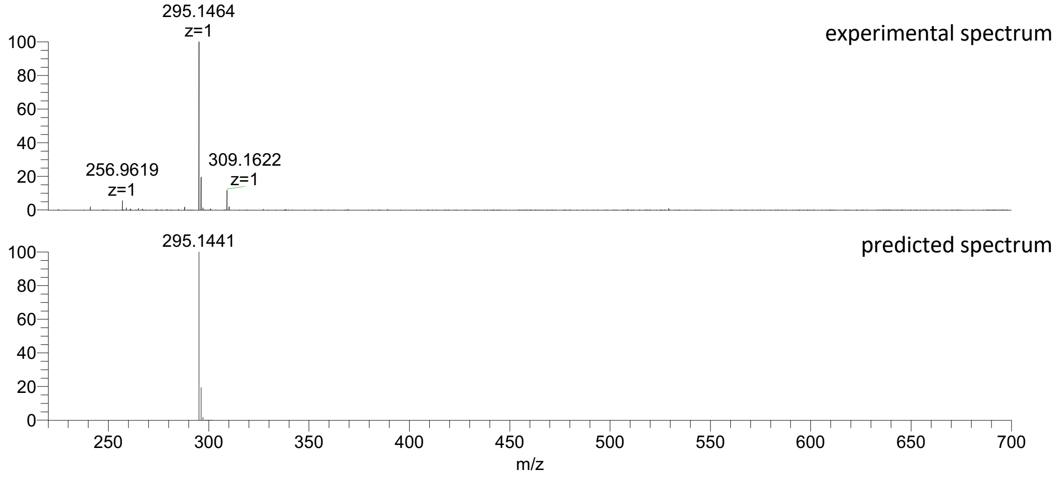

Figure S45: HRMS spectrum of compound **2b**.

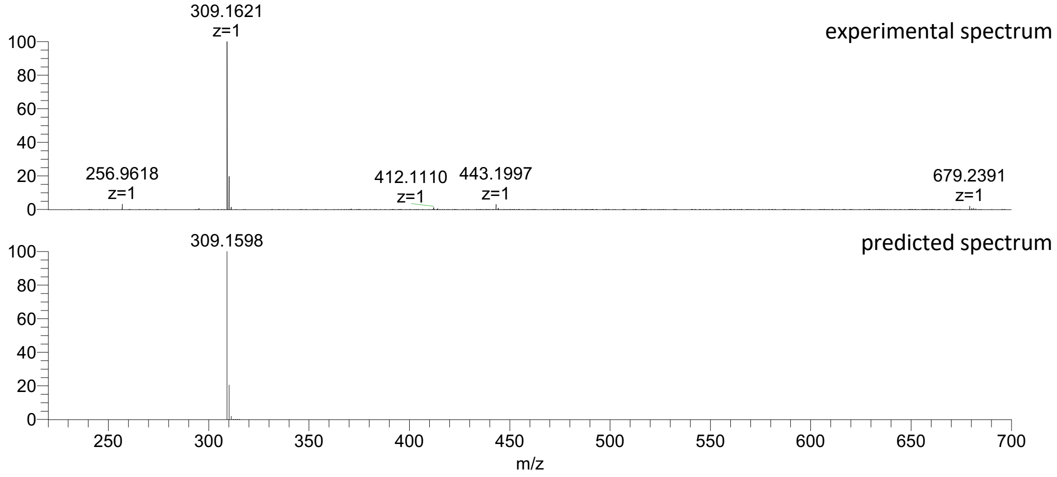

Figure S46: HRMS spectrum of compound **2c**.

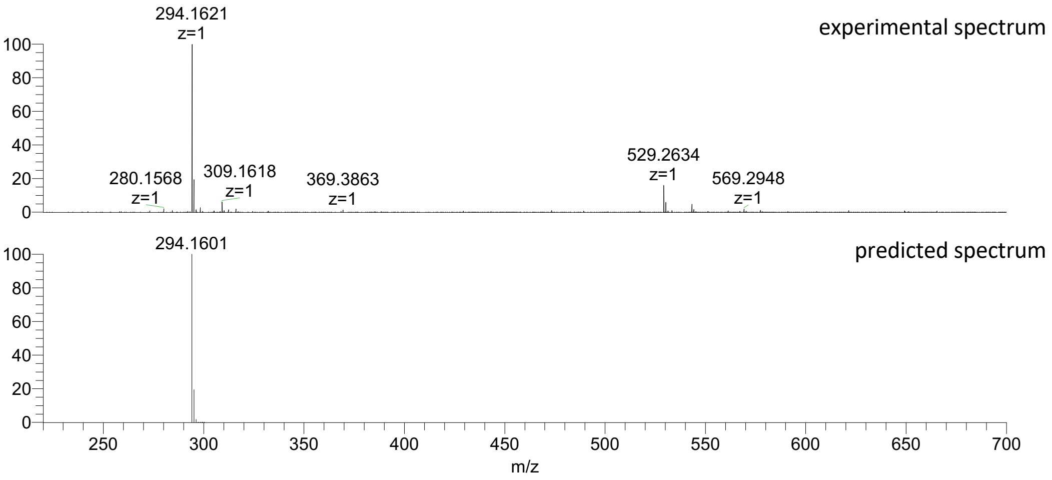

Figure S47: HRMS spectrum of compound **3a**.

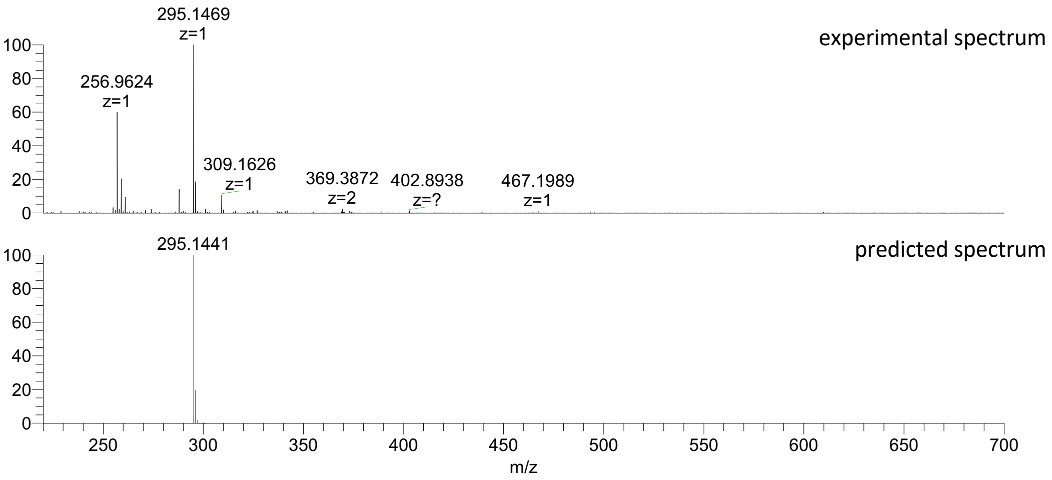

Figure S48: HRMS spectrum of compound **3b**.

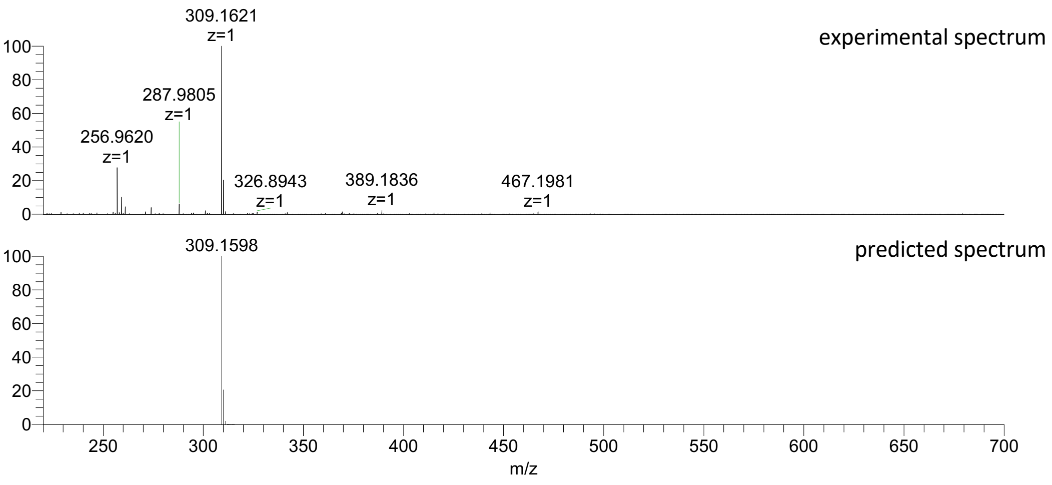

Figure S49: HRMS spectrum of compound **3c**.

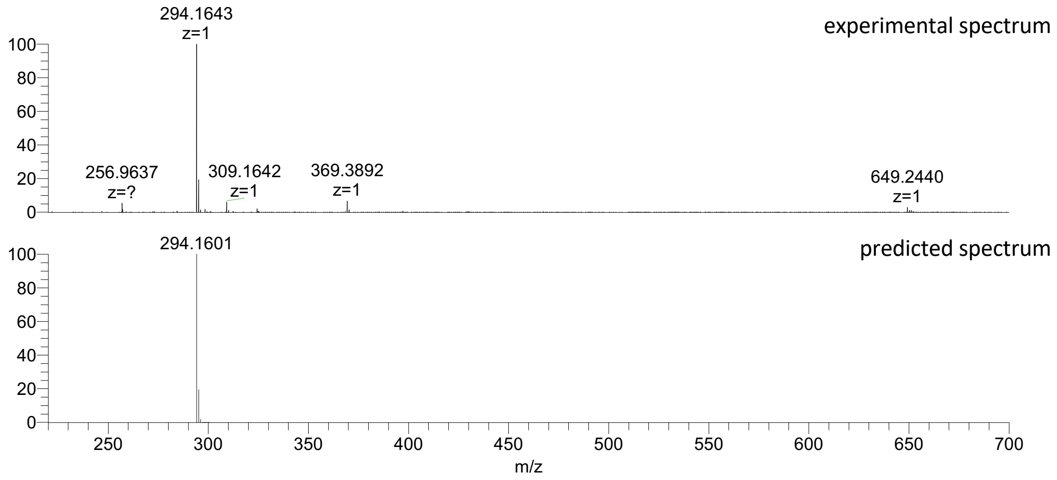

Figure S50: HRMS spectrum of compound **4a**.

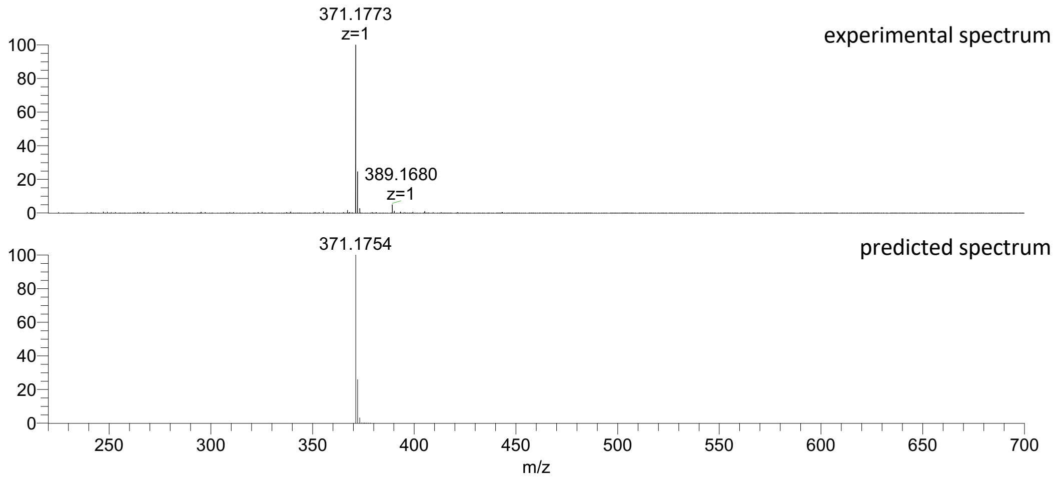

Figure S51: HRMS spectrum of compound **4b**.

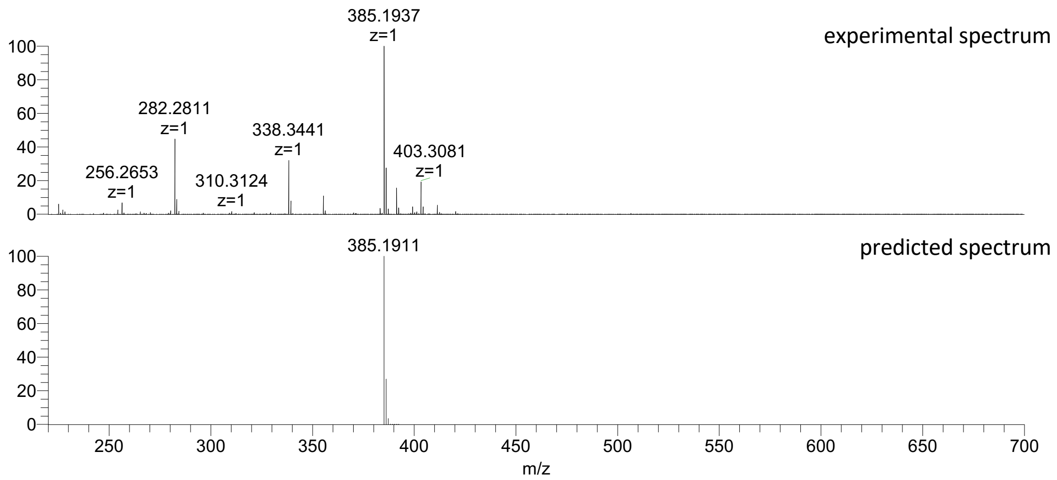

Figure S52: HRMS spectrum of compound **4c**.

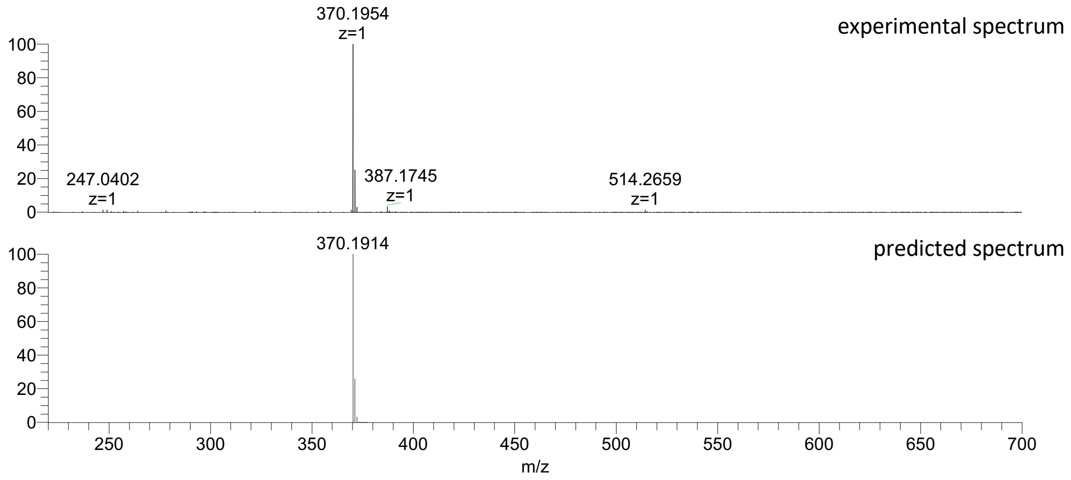

Figure S53: HRMS spectrum of compound **5a**.

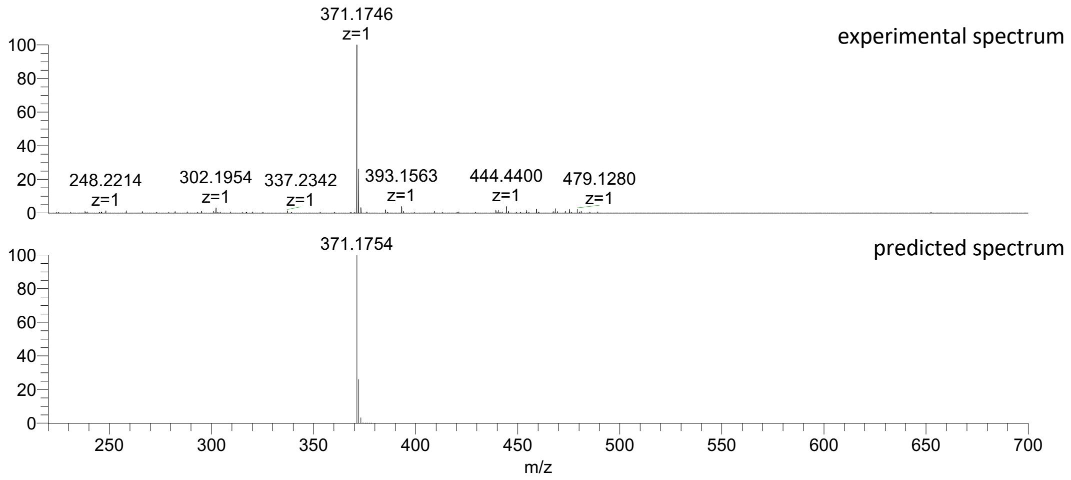

Figure S54: HRMS spectrum of compound **5b**.

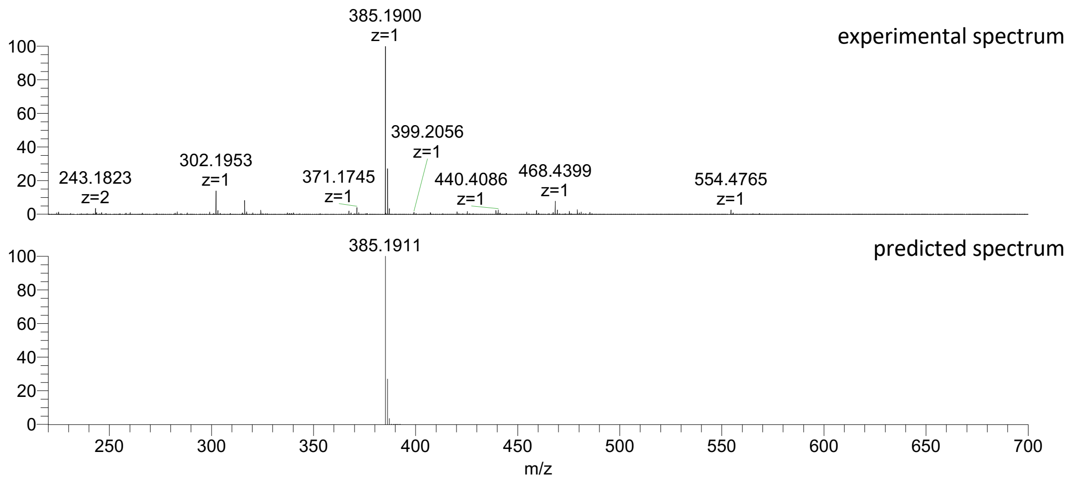

Figure S55: HRMS spectrum of compound **5c**.

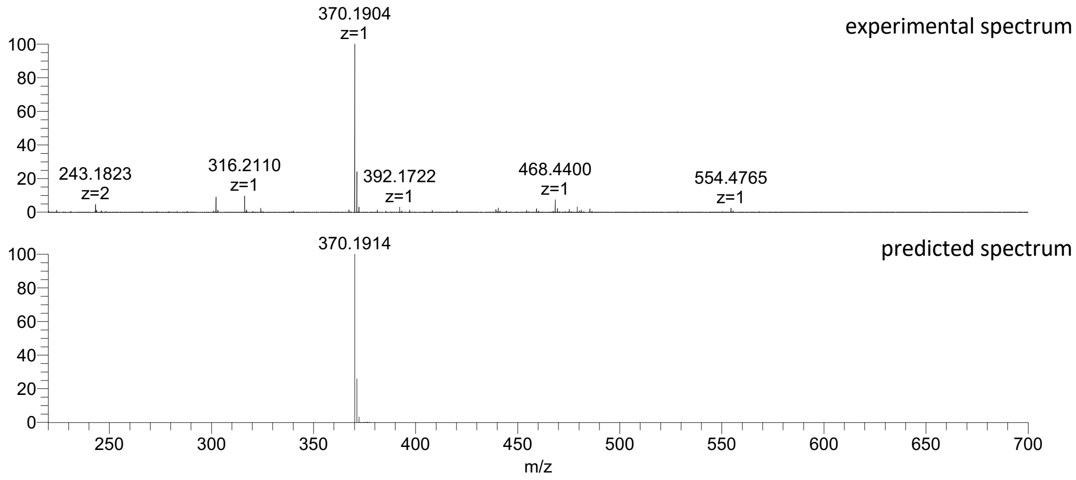

Figure S56: HRMS spectrum of compound **6a**.

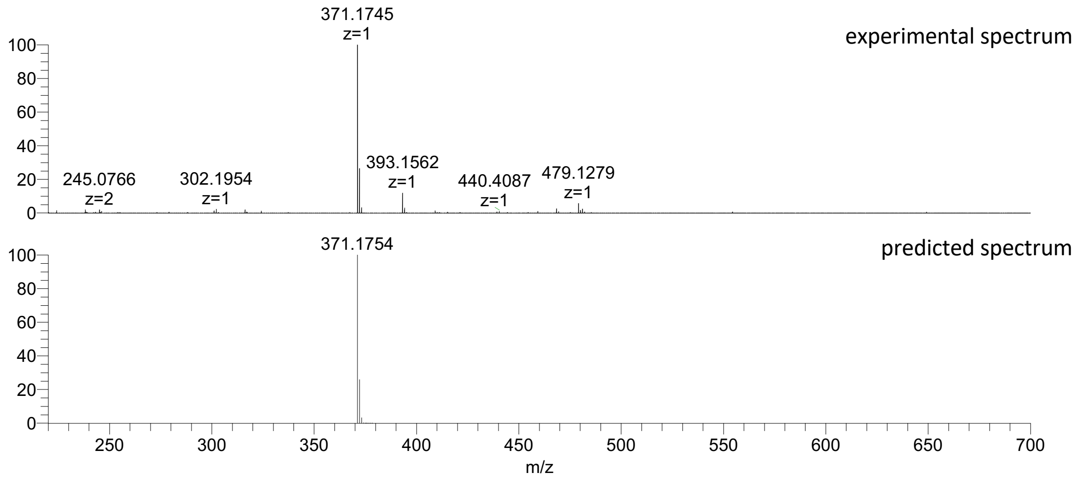

Figure S57: HRMS spectrum of compound **6b**.

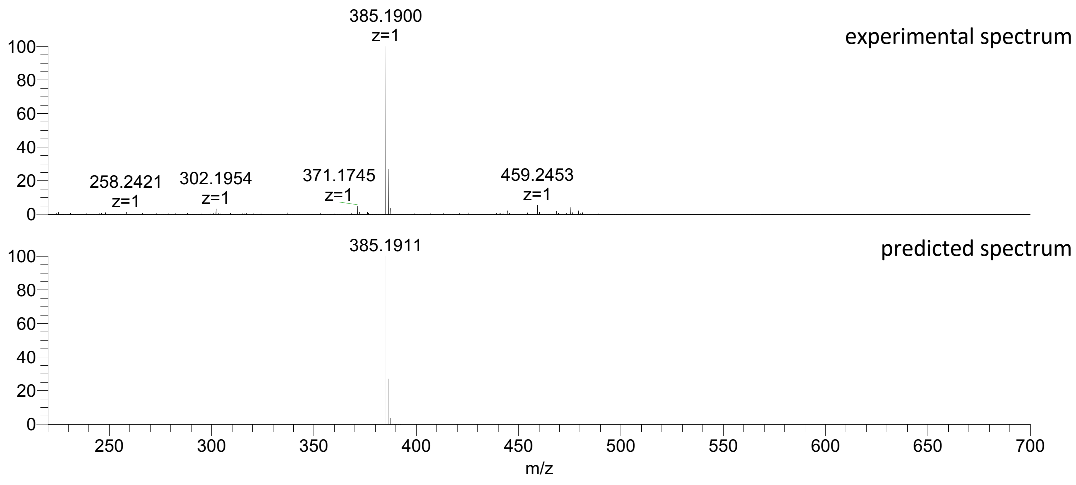

Figure S58: HRMS spectrum of compound **6c**.

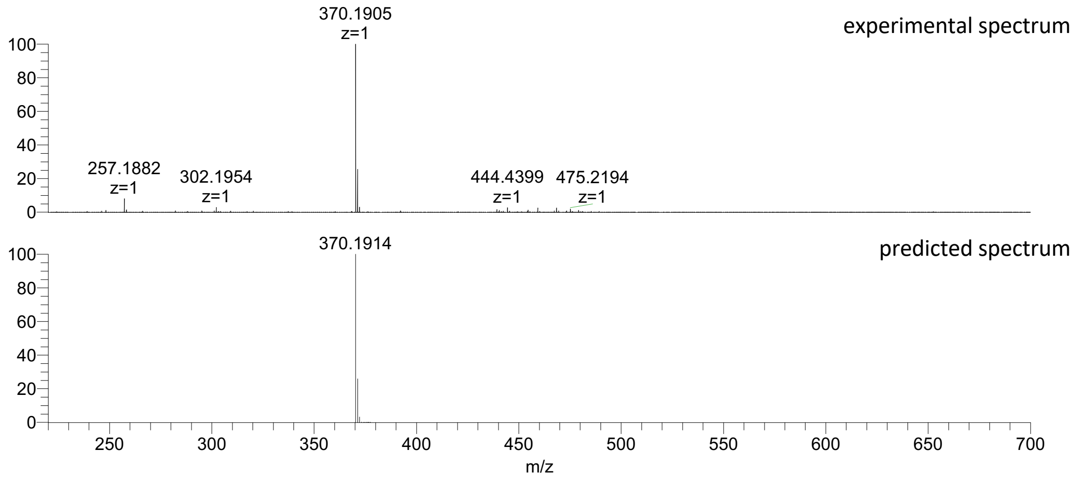

Figure S59: HRMS spectrum of compound **7b**.

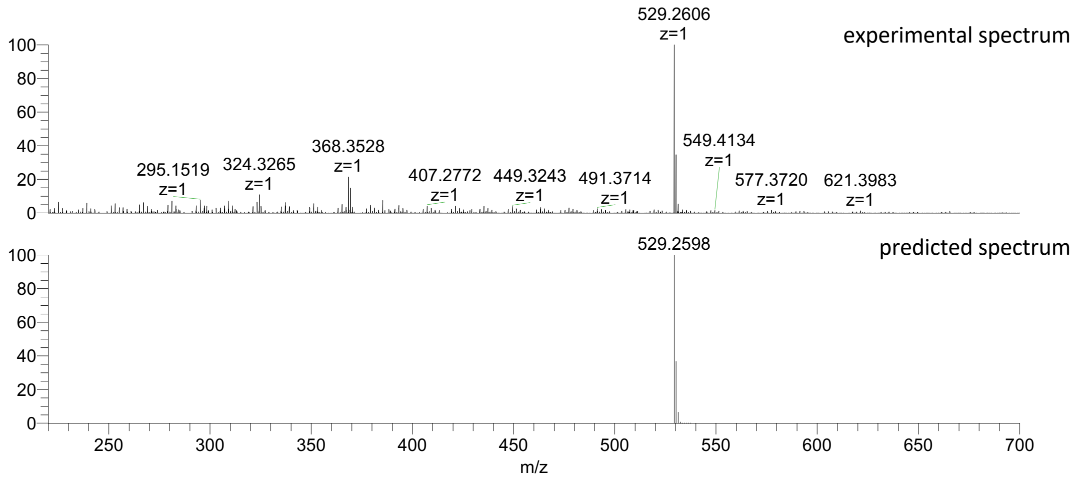

Figure S60: HRMS spectrum of compound **7c**.

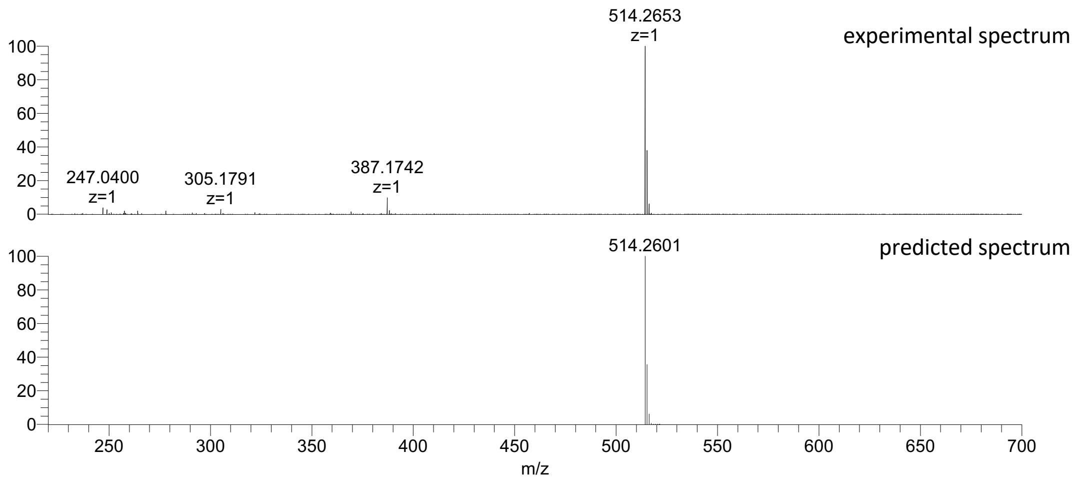

5. Biological data

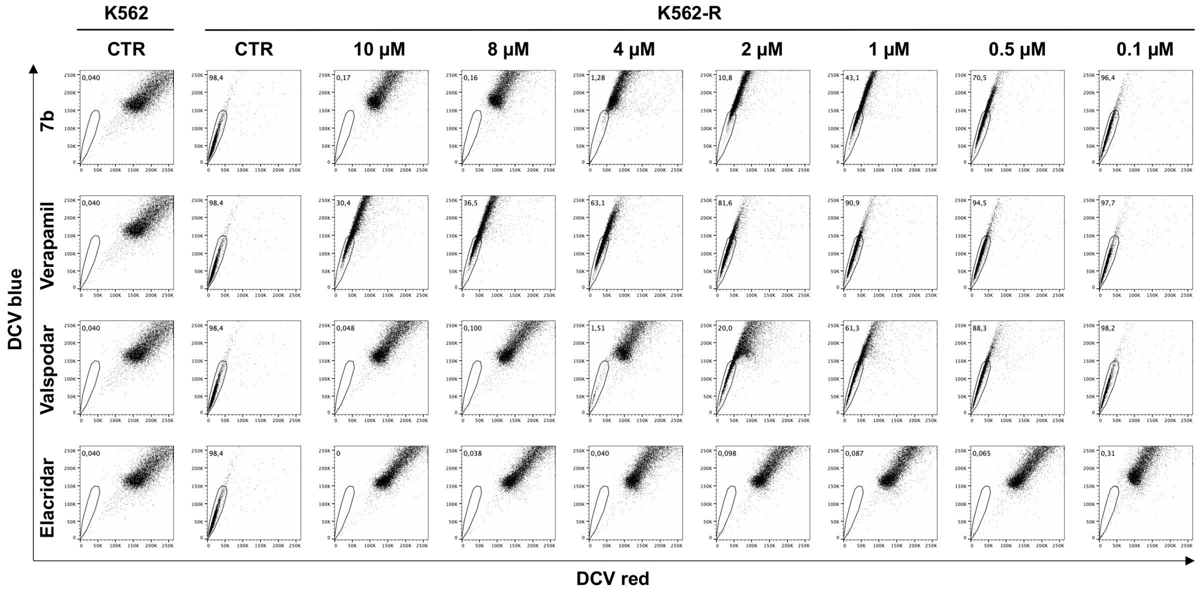

Figure S61: K562-R cells were stained with DCV and concurrently incubated with indicated concentrations of **7b** (telmi-ester), verapamil, valspodar, and elacridar. SP subsets were subsequently analyzed by flow cytometry. SP subsets are indicated by polygonal gates, and the percentage of cells within these gates is given.

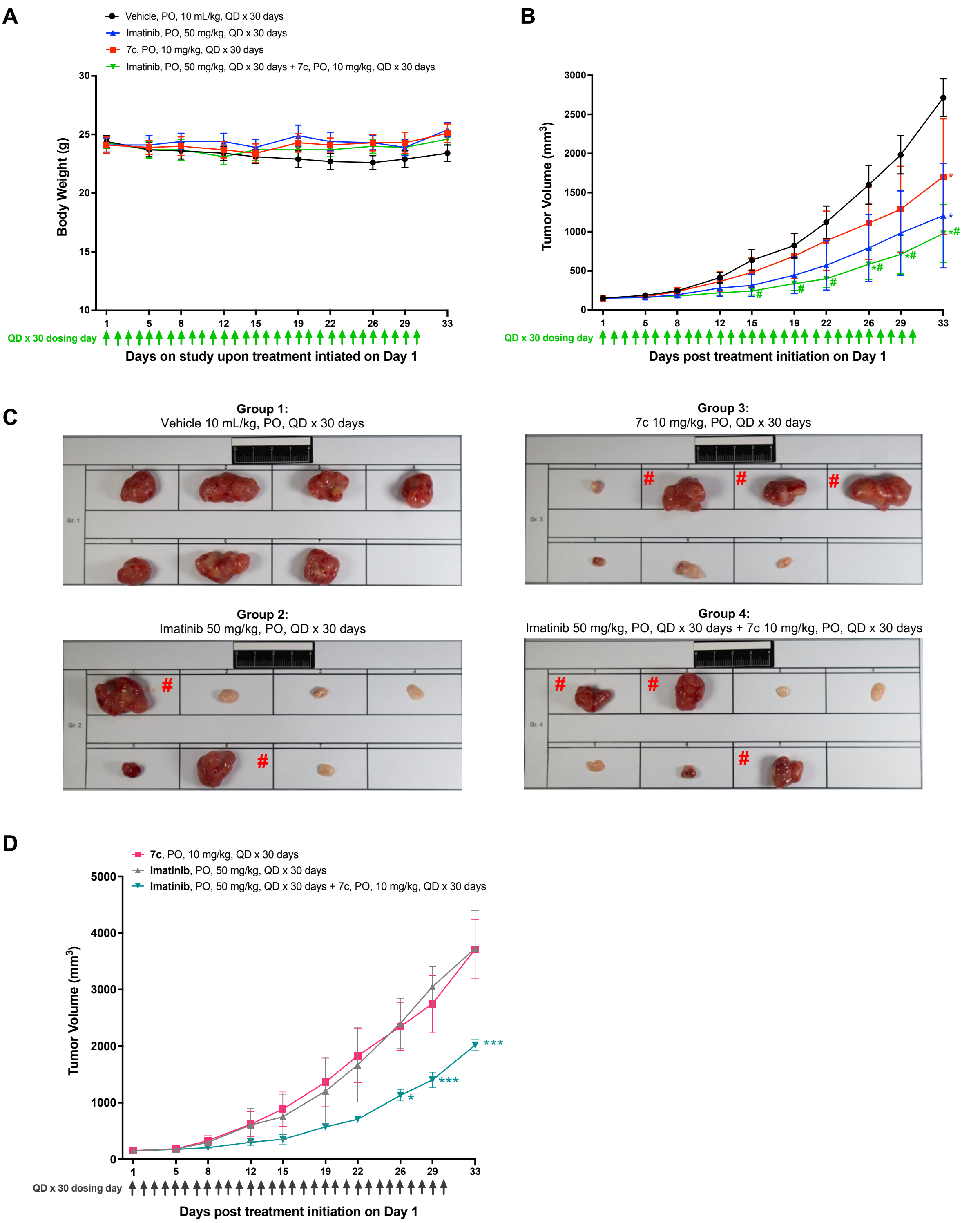

Figure S62: Efficacy evaluation of **7c** and imatinib in the human CML K562R xenograft model. Test animals, female NOD/SCID mice, were subcutaneously (SC) implanted with viable human CML K562R cells at 1 x 10^7 cells/mouse (0.2 mL/animal with 50% high concentration Matrigel) to the left flank. Treatment was initiated on day 1, when the mean tumor volume reached 148-149 mm^3, which was 14 days post-tumor cell implantation. The test articles, **7c** at 10 mg/kg and imatinib at 50 mg/kg, were orally (PO) administered once daily for 30 consecutive days (QD x 30 days) with a total of 30 dose administrations as monotherapy and combination therapy. Body weight was measured twice weekly from day 1 (treatment initiation) till the

study end date (day 33). **A)** Body weight change (%) over time (day). Error bars are SEM values. Two-way ANOVA followed by Tukey's multiple comparison test was conducted for statistical analysis. **B)** Tumor volume (mm^3) over time (day). Tumor growth, tumor volume by length x $(width)^2$ x 0.5, was measured twice weekly from day 1 (treatment initiation) till the study end date (day 33). Two-way ANOVA followed by Tukey's multiple comparison test was conducted for statistical analysis; *p<0.05 (reduction in mean tumor volume compared vehicle control). # indicates an anti-tumor activity compared to the vehicle control group as determined by %T/C (Treatment/Control) value ≤ 42%. A %T/C value ≤ 42% compared to that of the vehicle control group is considered significant anti-tumor activity according to NCI standards. Error bars are SEM values. **C)** On day 33, the tumor samples were harvested from all animals and photographed. Tumors from Groups 2 to 4 are marked with "#" to indicate cases of tumor progression. In contrast, every tumor in Group 1 (vehicle control) was classified as progressing tumor. **D)** Tumor growth, tumor volume by length x $(width)^2$ x 0.5, in a selected cohort of mice exhibiting tumor progression (see Figure S63C). Two-way ANOVA followed by Tukey's multiple comparison test was conducted for statistical analysis; *p<0.05, ***p<0.001 (reduction in mean tumor volume compared to **7c** monotherapy).

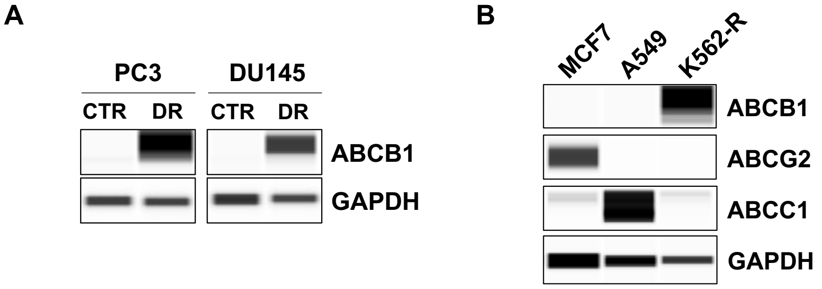

Figure S63: A) Jess Simple Western™ immunoassays determining the expression of ABCB1 in PCR, PC3-DR, DU145, and DU145-DR cells. GAPDH served as loading control. **B)** Jess Simple Western™ immunoassays determining the expression of ABCB1, ABCG2, and ABCC1 in MCF7, A549, and K562-R cells. GAPDH served as loading control.

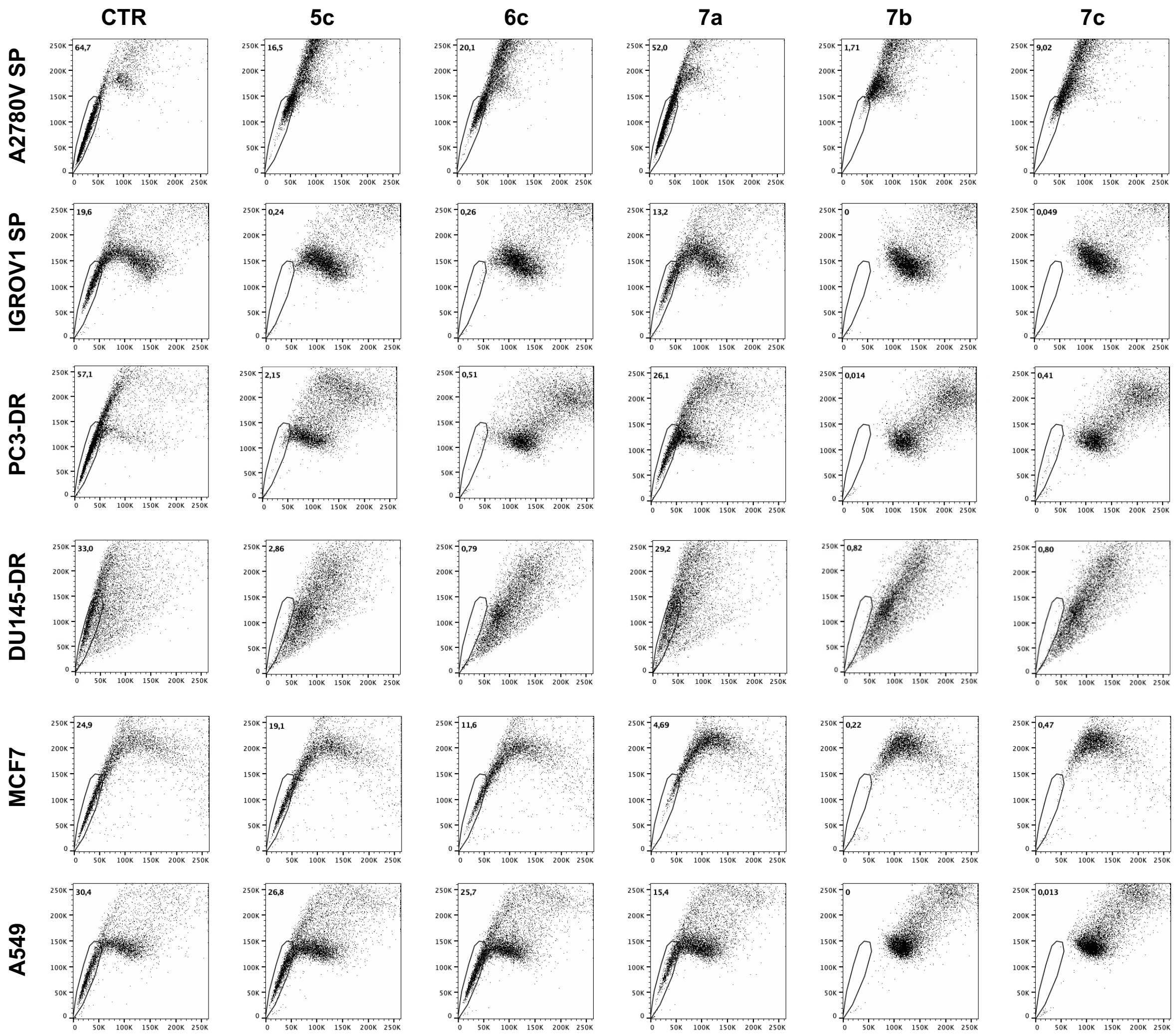

Figure S64: A2780V SP, IGROV-1 SP, PC3-DR, DU145-DR, MCF7, and A549 cells were stained with DCV and concurrently incubated with 10 µM of **5c**, **6c**, **7a** (telmi), **7b** (telmi-ester), or **7c** (telmi-amide). The subsequent analysis was carried out by flow cytometry. SP subsets are indicated by polygonal gates.
